# Inhibition of Autoimmune Chagas-Like Heart Disease by Bone Marrow Transplantation

**DOI:** 10.1371/journal.pntd.0003384

**Published:** 2014-12-18

**Authors:** Maria C. Guimaro, Rozeneide M. Alves, Ester Rose, Alessandro O. Sousa, Ana de Cássia Rosa, Mariana M. Hecht, Marcelo V. Sousa, Rafael R. Andrade, Tamires Vital, Jiří Plachy, Nadjar Nitz, Jiří Hejnar, Clever C. Gomes, Antonio R. L. Teixeira

**Affiliations:** 1 Chagas Disease Multidisciplinary Research Laboratory, Center for Research and Prevention of Neglected Diseases, Faculty of Medicine, University of Brasilia, Federal District, Brazil; 2 Laboratory of Protein Chemistry, Institute of Biology, University of Brasilia, Federal District, Brazil; 3 Laboratory of Viral and Cellular Genetics, Institute of Molecular Genetics, Czech Academy of Sciences, Prague, Czech Republic; Federal University of São Paulo, Brazil

## Abstract

**Background:**

Infection with the protozoan *Trypanosoma cruzi* manifests in mammals as Chagas heart disease. The treatment available for chagasic cardiomyopathy is unsatisfactory.

**Methods/Principal Findings:**

To study the disease pathology and its inhibition, we employed a syngeneic chicken model refractory to *T. cruzi* in which chickens hatched from *T. cruzi* inoculated eggs retained parasite kDNA (1.4 kb) minicircles. Southern blotting with *Eco*RI genomic DNA digests revealed main 18 and 20 kb bands by hybridization with a radiolabeled minicircle sequence. Breeding these chickens generated kDNA-mutated F1, F2, and F3 progeny. A *targeted-primer* TAIL-PCR (*tp*TAIL-PCR) technique was employed to detect the kDNA integrations. Histocompatible reporter heart grafts were used to detect ongoing inflammatory cardiomyopathy in kDNA-mutated chickens. Fluorochromes were used to label bone marrow CD3^+^, CD28^+^, and CD45^+^ precursors of the thymus-dependent CD8α^+^ and CD8β^+^ effector cells that expressed TCRγδ, vβ1 and vβ2 receptors, which infiltrated the adult hearts and the reporter heart grafts.

**Conclusions/Significance:**

Genome modifications in kDNA-mutated chickens can be associated with disruption of immune tolerance to compatible heart grafts and with rejection of the adult host's heart and reporter graft, as well as tissue destruction by effector lymphocytes. Autoimmune heart rejection was largely observed in chickens with kDNA mutations in retrotransposons and in coding genes with roles in cell structure, metabolism, growth, and differentiation. Moreover, killing the sick kDNA-mutated bone marrow cells with cytostatic and anti-folate drugs and transplanting healthy marrow cells inhibited heart rejection. We report here for the first time that healthy bone marrow cells inhibited heart pathology in kDNA^+^ chickens and thus prevented the genetically driven clinical manifestations of the disease.

## Author Summary

Chagas heart disease, stemming from infection by the protozoan parasite *Trypanosoma cruzi*, which is endemic in South America, is now present on five continents. The treatment now available for the clinically manifested heart disease is considered unsatisfactory. We postulate that manifestations of Chagas-like heart disease in a chicken model system refractory to *T. cruzi* infection might be prevented by killing the sick bone marrow cells of the recipient with anti-folate and cytostatic drugs, and transplanting healthy bone marrow cells from a syngeneic donor. We performed triplicate experiments in various groups of five chickens. Chickens hatched from *T. cruzi-*inoculated eggs, retaining the parasite mitochondrial kDNA in the genome, developed Chagas-like heart disease and died. The integration of *T. cruzi* kDNA minicircles into the chicken genome and the heritability of that integration were recorded, and the genetic underpinning of the autoimmune heart disease was demonstrated. Genome modifications in kDNA-mutated chickens led to disruption of immune tolerance and thus explained, for the first time, the rejection of the heart. Moreover, we found that killing the sick kDNA-mutated bone marrow cells with cytostatic and anti-folate drugs and transplanting healthy marrow cells inhibited heart rejection. These results suggest that bone marrow transplantation could be used to prevent Chagas heart failure.

## Introduction

Epidemiological methods have been used to evaluate the incidence and distribution of endemic Chagas disease, to associate epiphenomenal clinical manifestations with the recognized pathology of the disease. Acute *T. cruzi* infections often resolve spontaneously, but the individual remains chronically infected for his or her lifetime, even in the absence of clinical manifestations [1]. Approximately 18 million people on five continents harbor often-asymptomatic chronic *T. cruzi* infection [1], [2]. In humans, approximately 30% of the individuals infected with *T. cruzi* develop chronic Chagas heart disease (94.5%) and/or megacolon and megaesophagus (5.5%) [3]. Additionally, Chagas disease can present with complex clinical manifestations, such as myositis and weakness, and peripheral nervous system involvement, which translates into a neuroendocrine syndrome [4]–[6]. The relationship between cryptic *T. cruzi* infection and late manifestations of Chagas heart disease, three or more decades after parasite acquisition, has been debated [1]. Two main theories have been proposed for the pathogenesis of Chagas disease. The parasite persistence theory suggests that the mechanical disruption of the parasitized cells by the parasite weakens the heart and initiates heart failure [3, 7, and 8]. The autoimmune theory suggests that the pathogenesis of Chagas disease, which is usually seen decades after acute infection, when the parasite is not in close proximity, might be associated with the rejection of target cells by competent effector lymphocytes [9], [10].

The foundation of immunology is based on the premise that the immune system evolved primarily to protect against pathogens, foreign cells, and inorganic and organic toxic substances. The body maintains homeostasis through immunologic surveillance [11]. In this regard, immunologic surveillance is permissive to the continuous elimination of aging cells in the body, with minimal rejection under physiological conditions [12]–[14]. However, the phenomenon of autoimmunity occurs when the immune system recognizes components of a healthy individual's body [15] due to a breakdown in immune tolerance to the body's own constituents [16]. The disruption of immune tolerance and the resultant exacerbation of anti-self immune reactions and noxious rejection of target tissue define autoimmune disease [17].

More than 100 autoimmune diseases can be accurately diagnosed on the basis of symptoms that are validated by pathology results revealing multifaceted, unresolved inflammation [18], [19]. However, the etiology of almost all autoimmune diseases is unknown, thus precluding effective treatment to block the as-yet-undisclosed factors that can trigger hypothetical antigenic mimicry, whereby the immune system destroys its own tissue [20], [21]. Although the pathology of autoimmune diseases has long been thought, the exact event that triggers the onset of inflammation is controversial. The relationship between infectious agents and autoimmunity has been considered a first signal for the induction of autoimmunity by molecular mimicry, yet its origin has not been recognized. The triggers of auto immune diseases under complex circumstances associate genetic susceptibility and exposure to uncertain environmental factors capable of activating the first signal-specific immune system reaction pathways [21], [22]. Additionally, a second signal non-specific reaction includes several factors, such as adjuvant effect, bystander activation of auto reactive T cells; and modified immunogenic self-antigens can lead to autoimmunity against self-antigens, resulting from random mutations and protein synthesis errors, which can be recognized as foreign by B and T lymphocytes [23], [24]. Accordingly, the self-antigen might interfere with peripheral immune tolerance during an infection that calls out bystander activation of T and B cells with specificity for mutated self-antigen, which leads to epitope spreading, production of immune competent cells reactive against self-antigens or against putative super antigens, and enhanced presentation of self-antigens, cytokines release post-immune cell activation, and necrobiosis following apoptosis, and general inflammation [24].

Recent studies have focused on the relationships between autoimmune diseases and environmental factors, searching for endogenous intracellular factors in tissue and exogenous triggers on the cell surface [25]. Identifying candidate environmental factors has been difficult due to an unpredictably long period of time between the onset of infection and the appearance of recognizable clinical autoimmune disease. Furthermore, latency-prone microbes can remain within their hosts for decades without triggering autoimmune disease in the majority of patients. The identification of internal and external autoimmune factors from conception to death requires exposome and infectome approaches, focusing on identifying candidate infectious agents from environmental exposure that can trigger autoimmune diseases [26]. These approaches have implicated 16 bacterial species, three viruses, two protozoa, one helminth, and one fungus as inducers of autoimmune diseases [27]–[29]. Although an autoimmune disease can begin before the onset of overt symptoms, it is believed that subclinical manifestations, which might have occurred for an undefined period of time, could improve our understanding of late-phase ailments, as well as frequent remissions and relapses. A familial predisposition to autoimmune diseases has been recognized, and studies in homozygotic twins have suggested the involvement of certain genes in the pathogenesis of this category of human diseases [30], [31].

The role of antibodies for self-tissue antigens has been proposed to explain the onset of mechanisms whereby infectious agents may induce sensible alterations in the immune responses, disruption of immune tolerance, and clinical manifestations of autoimmune disease. Although antibody production increases with aging, its pathological effect translating clinical manifestation was difficult to determine [32]–[34]. In a non-human primate model the development of autoantibody response in healthy baboons was shown, gradually increasing concentrations of antinuclear antibodies, whole-cell-antibodies, and natural autoantibodies from youth to old age [34]. In the primate model, the immunoglobulin levels and cytokines that promote immune deregulation remained unchanged, and thus the autoantibodies were produced in absence of clinical and pathological manifestations [33]. A body of evidence has been produced that favors a role of cardiac myosin autoantibodies in patients with dilated cardiomyopathy (DCM), as IgG3 reactivity correlated with myocardial dysfunction [35]. Also, it has been proposed that myocarditis often produced by viral infection may develop into autoimmune inflammatory cardiomyopathy and heart failure [36]. The significant role of myosin IgG autoantibodies, which reacts with the beta-adrenergic receptor and triggers cAMP-dependent protein kinase A signaling in heart cells, was suggested [36]. The cross-reactive autoantibodies against human cardiac myosin and the beta-adrenergic receptor in the heart may play a mechanistic role in the pathogenesis of DCM [36]. Additionally, subclass specific autoantibodies against myosin has been considered pro-inflammatory moieties, and it has been postulated that pro-inflammatory IgG3 antibodies may play a role in the autoimmune mechanisms of injury in DCM patients [37]. Viral myocarditis and valvulitis are often associated with autoimmunity because anti-heart antibodies have been identified in the sera of patients with this ailment, as well as in low titers in healthy individuals [38]. Furthermore, the induction of experimental autoimmune myocarditis (EAM) and of CVB3 myocarditis in several mouse strains and in the Lewis rat requires emulsification of cardiac myosin and CVB3, separately, with adjuvant for elicitation of a second signal pro-inflammatory response, but the clinical implications of such anti-self antibodies and its prognostic significance has not been fully understood [39]. Chagas disease has been considered a clinical condition potentially fatal due to an inflammatory autoimmune cardiomyopathy [40]. In this regard, the molecular mimicry between the cardiac myosin heavy chain and *T. cruzi* protein B13 has suggested that the Chagas heart lesions may be triggered by parasitic antigen-specific effectors T cells [41]. Interestingly, a putative role played by autoantibodies in human Chagas heart disease was controversial [42], because autoantibodies produced against troponin and myosin were not essential for cardiac inflammation [43]–[47]. It was further shown that cardiac damage induced by immunization with heat-killed *T. cruzi* was not antibody mediated [48]. Attempts to produce the autoimmune lesions of Chagas heart disease by immunization with *T. cruzi* antigens B13, Cha, cruzipain, and 45-kDa calreticulin required adjuvant in order to produce inflammatory infiltrates in the heart [49]–[54].

Some animal model experiments have been proposed to complement hypothetical autoimmunity mechanisms [20]. To substantiate these mechanisms, experimental animal models were described for Hashimoto's thyroiditis [55], experimental acute encephalitis (EAC) [56], and CVB3 myocarditis [57], whereby lesions were produced, respectively, by injections of thyroglobulin, myelin basic proteins (MBP), or CVB3 antigens, each mixed with adjuvant [56]–[57]. The presence of antibodies against myosin has been recorded for several inflammatory myocardiopathies and for DCM. Furthermore, attempts to reproduce the autoimmune lesions of Chagas disease by injections of myosin, and of wild or recombinant *T. cruzi* antigens mixed with Freund's adjuvant revealed small lymphocyte infiltrates in the heart and other bodily tissues [58]–[61]. Interestingly, the attempts of passive transfer the autoimmune phenomenon by injections of lymphocytes from immunized animals to naive recipients produced localized inflammatory reactions in the absence of specific clinical symptoms and of gross lesions [1], [58]–[64]. Yet, it has been proposed that the origin of autoimmune diseases can be associated with somatic mutations [17]. In the case of Chagas disease, it has been described that somatic mutations, resulting from the *T. cruzi* kDNA minicircle sequences that integrate into the genomes of humans, rabbits, and chickens can lead to severe autoimmune disease manifestations and gross pathology [10], [65]–[66]. Moreover, in the absence of clinical manifestations of autoimmune disease associated with autoantibodies [42], [45]–[47], we hypothesize that Chagas disease can be an antigen-independent autoimmune phenomenon, whose first signal is provided by somatic mutations driven by the overreactivity of cytotoxic T lymphocytes, which infiltrates and reject target tissues in the parasite-free transkingdom chicken model system.

The eukaryotic protozoa in the order Kinetoplastida and family Trypanosomatidae include the highly diversified *T. cruzi* [66]–[68]. Insect-borne primary infections result from the contamination of skin wounds and mucosal surfaces with *T. cruzi* forms. Upon entry into the host's histiocytes, the trypomastigotes congregate and transform into amastigotes, which are replicative forms that de-differentiate and reinitiate their life cycles in non-phagocyte host cells [1], [67]–[71]. Electron microscopy analysis of *T. cruzi* forms has revealed two organelles containing DNA, namely nuclear DNA (nDNA) and the symbiotic mitochondrial kinetoplast DNA (kDNA) [71]. The incidences of nuclear and mitochondrial phylogenetic incongruences indicate that widespread genetic recombination continues to influence the structural diversity of the *T. cruzi* population [70]. The kDNA constitutes approximately 25% of the total cellular DNA and is organized into a network of maxicircles and minicircles. There are a few dozen maxicircles of 40 kb and approximately 20,000 *T. cruzi* minicircles averaging 1.4 kb, which translate guide RNA (gRNA) to edit the maxicircle genes [68]–[70]. The *T. cruzi* kDNA minicircle encloses four variable regions (VRs), interspersed by conserved regions (CRs); each region encodes constant sequence blocks – CSB1, CSB2, and CSB3 – in which cytosine- and adenine-rich (CA-rich) motifs represent specific sites for the initiation of replication, transcription, recombination, and lateral DNA transfer [65]–[67]. The *T. cruzi* mitochondrial symbiont kinetoplast network organization, which signals the replication and/or latency of parasitic forms living free in the mammalian cytoplasm, favors host–parasite coevolution, horizontal gene transfer and genetic diversity [65]–[71].

Studies that were conducted in human chagasic families revealed instances of naturally occurring *T. cruzi* mitochondrial kDNA minicircles integrated mainly into retrotransposable long interspersed nuclear element (LINE-1) loci on various chromosomes [10], [65]–[67]. The kDNA integration events documented the contemporary transfer of eukaryotic DNA to the human genome and its subsequent inheritance by descendants, suggesting that the disease pathogenesis intermingles new concepts of evolutionary biology and medicine [10], [65]–[67].

Vertebrate genomes are largely composed of highly repetitive viral sequences, and these molecules have been used to decipher the evolution of the genome architecture over millions of years [71]–[78]. Approximately 50% of the human genome consists of class 1 and 2 transposable elements (TEs) of viral origin [79]–[82]. The class 1 TEs include the autonomous LINEs, which are retrotransposons that encode endogenous machinery for accomplishing reverse transcription [77]–[81] and mobilization of the DNA copy to different distant sites in the genome [66, 67, 76, 81, and 83]. The human genome contains 400,000 truncated copies of retrotransposons, including 400 active autonomous LINEs, which are homologs of CR1 repeats in the chicken genome [84], [85]. Coincidentally, a variety of repetitive elements act as open gates for the integration of exogenous DNA in TEs. These gates are natural tools for further mobilization, recombination, hitchhiking and shuffling [62, 65, 80, and 81]. Interestingly, the mitochondrial minicircle kDNA sequences from *T. cruzi* can integrate into LINE-1 with CSB-mediated microhomology, and through the target site, reverse transcription can copy the kDNA minicircles that hitchhike to second- and third-party coding regions, which later appear recombined and shuffled at various chromosomes [62, 65, and 81].

The previous demonstration of the lateral kDNA transfer (LkDT) of minicircle sequences from T. cruzi to vertebrate animals [10], [65]–[67], [81]–[87] suggested the concept of genotype modifications as the main driving force for autoimmunity, and therefore, a possible relationship between insertional mutagenesis and the pathogenesis of human Chagas disease was considered [1], [10], [65]–[67], [81]–[87]. Based on our previous demonstration of gross Chagas heart pathology in kDNA-mutated outbred chickens, a growing body of evidence has suggested a relationship between LkDT and Chagas disease [1], [10], [65]–[67]. This hypothesis was bolstered by findings from a chicken model system that is refractory to the infection and that eliminates any contamination with DNA from cryptic T. cruzi infections [10], [65]. The infection of fertilized eggs prior to incubation promptly reached the embryonic stem cells, and the minicircle kDNA sequences were retained in the chicken genome, but the parasites were all eliminated at an early stage of immune system development. Therefore, later effects were caused by insertional mutagenesis [10], [65]–[67]. A dearth of research has examined the innate and immune effector systems' roles in normal embryo development or during congenital infections [88]–[90]. The innate chicken non-immune effector system is neither developed nor functional in the embryo, and the cellular aspects of the immune response mature sequentially in the fetus [65–67, and 88]. The lack of a functional immune system makes the embryo highly susceptible to invading microbes, which may lead to abortion, natal death, or perinatal death, but the chick's survival and development often occur in the presence of immune tolerance [1], [10], [65]–[67], [88]–[90].

The parasite-free chicks that hatch from T. cruzi-inoculated eggs nonetheless transfer the kDNA minicircles retained within their genomes to their progeny via breeding. The kDNA-mutated chickens (kDNA+) reject the heart, exhibiting lysis of the myocardium fibers by immune effector cells. Notably, the kDNA mutations are vertically transferred to progeny that subsequently develop Chagas-like heart disease [10], [65]–[67].

This study was based on our hypothesis that the genetically driven autoimmune rejection of heart tissue in Chagas-like disease [10], [65]–[67] can be prevented by replacing sick (kDNA+) bone marrow cells (BMCs) with healthy cells from naive control (NC) chicken marrow cells. Accordingly, we used the Prague syngeneic lines CB (B^12^/B^12^) and CC (B^4^/B^4^) to avoid some graft rejections that can occur in partially inbred or even outbred chickens [91]–[95]. We then introduced genetic modifications into the genomes of the syngeneic chickens by the inoculation of *T. cruzi* into fertilized eggs. The chickens that hatched from the *T. cruzi*-infected eggs retained the parasite mitochondrial kDNA in their genomes and rejected the parasite-free hearts. Parental breeding generated kDNA+ descendants, and heart disease was documented in these progeny. These results confirm and extend our findings in the outbred chicken model system [10], [65]–[67]. Moreover, this study focused on the hallmark manifestation of Chagas heart disease and revealed that healthy bone marrow transplantation inhibited heart pathology in the kDNA+ chickens and thus prevented the pathology-driven clinical manifestations of the disease.

## Materials and Methods

### Gallus gallus

The fertile eggs of the Prague congenic lines CB (B^12^/B^12^) and CC (B^4/^B^4^) were a donation from the Institute of Molecular Genetics, Academy of Sciences of the Czech Republic. Chicks that hatched and grew to adulthood were bred to generate flocks at the Laboratory Animal Facility of the University of Brasilia. The chicken house was maintained at an average temperature of 24°C, under positive-pressure filtered air and exhaust.

To prevent contamination, only authorized personnel entered the chicken house, and these personnel wore boots, apron, gown, and mask when handling the flock. The chickens were fed commercial chow supplemented with essential amino acids and oyster shell as the calcium source. The CB and CC chickens were housed in cages located in separate aisles, and the eggs fertilized through artificial insemination were incubated at 37°C for 21 days to hatch chicks that reached sexual maturity at eight months of age.

### Ethics statement

The protocols (UnBdoc #35714/2008) used in these studies were approved by the Institutional Ethical Committee in Animal Research - CEUA, Institute of Biology, University of Brasilia, in accordance with the International Animal Welfare Act (7 U.S.C. 2131 et. seq.), applicable guidelines and policies, and the U.S. government's Principles for the Utilization and Care of Vertebrate Animals Used in Testing, Research and Training.

### Growth of parasites and inoculation of *Trypanosoma cruzi* into chicken eggs

The trypomastigote and epimastigote forms of the *T. cruzi* Berenice stock were grown as described elsewhere [10], [65]–[67]. Fertile eggs laid by chickens with haplotypes CB (B^12^/B^12^) and CC (B^4^/B^4^) were inoculated with 100 *T. cruzi* trypomastigote forms in 10 µl of culture medium through a 1-mm-diameter aperture made in the shell at the air chamber [66]. An equal number of mock control eggs (mock) were inoculated with 10 µl of culture medium. The holes in the shell were sealed with adhesive tape, and the eggs were immediately transferred to incubation chambers maintained at 37°C with 65% humidity, with gentle rolling for 1 min every 30 min. The embryo development was monitored periodically, and DNA from the mononuclear cells of chicks that were hatched from naive control (NC) chickens that had never been under exposure to *T. cruzi* and from infected chicken eggs was used to assess the parasite kDNA integration [10], [64]–[67].

### Immunization of chickens with formalin-killed *T. cruzi* epimastigotes

Ten months-old NC chickens were used in the immunization procedures. The *T. cruzi* epimastigotes grown in liver-infusion tryptose medium were harvested in the exponential growth phase. The cells collected by centrifugation at 1000 g×20 min were washed in PBS and suspended in 4% (w/v) paraformaldehyde (Sigma-Aldrich). A total of 10×10^7^ formalin-killed epimastigotes suspended in 1 ml of PBS, pH 7.4, was injected subcutaneously in the tights of 10 pre-immune NC chickens. Three subcutaneous injections were administered one week apart and blood for immune serum was collected one week after the last injection. Also, sera collected from pre-immune and from immune NC chickens were stored in glycerol (1∶1 v/v) at –20°C.

### Nucleic acid analysis by PCR and Southern blot

DNA was extracted from peripheral blood mononuclear cells of kDNA+ chickens, NC chickens, and mock control chickens hatched from eggs inoculated with culture medium only. DNA was extracted also from somatic cells of the heart, kidney, skeletal muscle, large bowel, liver, and spleen from kDNA+ and from NC chickens. The *T. cruzi* epimastigotes' mitochondrial kDNA was extracted as described [10], [66].

The samplings of genomic DNA were used as templates for PCR amplification with the specific *T. cruzi* nDNA primers Tcz1 and Tcz2 [96] and the kDNA primers s35 and s36 [97]. The amplification reaction was run with 200 ng template DNA under the following conditions: 0.2 µM of each primer, 2.5 U *Taq* DNA polymerase, 0.2 mM dNTP, and 2 mM MgCl_2_ in a 25 µL final volume. Triplicate amplification reactions were performed using the recommended temperatures for nDNA (95°C for 5 min; 30 cycles of 95°C for 30 s, 68°C for 1 min, and 72°C for 1 min; and final extension at 72°C for 5 min) and kDNA primers (95°C for 5 min; 35 cycles of 95°C for 30s, 62°C for 1 min, and 72°C for 1 min; and final extension at 72°C for 5 min). The amplicons were resolved in 1.3% agarose gel, transferred to a positively charged nylon membrane (GE Life Sciences) by the alkaline method, and subsequently hybridized with specific [α-^32^P] dATP-labeled probes using a Random Primer Labeling Kit (Invitrogen).

Southern hybridizations were performed with *Eco*RI digests of DNA samples from NC and from kDNA+ chicken DNA. The positive control was the wild-type kDNA purified from *T. cruzi*. The *Eco*RI enzyme made a single cut in the kDNA minicircles to generate a linear fragment that was detected as a 1.4 kb band. The protocols for Southern hybridization are described elsewhere [10], [66].

### The tpTAIL-PCR, cloning and sequencing of the host DNA-kDNA chimeras

The *tp*TAIL-PCR was employed as described [10], [67]. The chicken primers annealing to a specific locus were obtained by the alignment of Genbank chimera sequence FN599618 within the locus NW_001471673.1 on chromosome 3 in the *Gallus gallus* genome [10]. The primers used in these studies with their respective annealing temperatures are shown in S1 Table. The kDNA primers were used in separate combinations with 0.04 µM of the CC1 to CC6 primers set, and three cycles of amplifications were run, as described elsewhere [10]. Clones selected by hybridization with a radio labeled kDNA probe were sequenced commercially. The *tp*TAIL-PCR was validated in a mix of 300 pg of kDNA from *T. cruzi* with 200 ng of DNA from control birds never exposed to kDNA [10].

The chimera-specific genes were selected, and primers were obtained for the metal transporter *CNNM2* (chromosome 6, NW_003763812.1), the mitochondrial NADP-dependent malic enzyme (*NADPME*) (chromosome 1, locus NW_003763650.1), and the dystrophin gene (chromosome 1, locus NW_001471534.2) chimera sequence (FN598991). The primer sets for dystrophin and *NADPME* were used in combination with the kDNA primers in a nested PCR, and the dilutions from the previous amplifications were maintained [10].

### Preparation of antigens

The hearts from kDNA+ and from naive control chickens (NC) were excised at the base of the large vessels and washed in three changes of cold PBS, pH 7.4 to eliminate the blood cells. The myocardium was cut into small (2 to 3 mm) fragments, which were suspended in hypotonic lysis buffer (0.1 mM HEPES, pH 7.9 at 4°C, 1,5 mM MgCl_2_ and 10mM KCl) with protease inhibitors (0.2 mM leupeptin, 0.5 mM TLCK and 0.5 mM DTT) [61]. The fragments were teased with a blade, and the cell clumps were mechanically disrupted in a glass tube with a teflon pestle accelerated at 14,000 rpm on ice. After six disruption cycles of 30 seconds separated by 5 min intervals, the crushed material was centrifuged at 5,000 rpm for 15 min at 4°C. The pellet was discarded, and the supernatant was centrifuged at 14,000 rpm for 10 min. The *T. cruzi* soluble antigens were obtained from 10×10^7^/ml epimastigotes forms [98]. The cells were centrifuged at 1000×g for 20 min, at 4°C, and the pellet was suspended in lysis buffer and centrifuged at 10,000×g for 10 min. The soluble proteins in the supernatant from the last centrifugation were obtained [98]. The heart and the *T. cruzi* soluble fractions were stored at −80°C until use.

The *T. cruzi* epimastigotes (10×10^7^/ml) in 10 ml of medium were centrifuged at 1000×g for 20 min, and the pellet was washed in three changes of cold PBS. The pellet was suspended in lysis buffer, and the soluble proteins in the supernatant from the last centrifugation were obtained, as described [99]. The heart and the *T. cruzi* soluble fractions were stored at −80°C until use.

### Enzyme-linked immunosorbent assay (ELISA)

We sensitized 96-well flat-bottom microplates with 1 µg of protein in 50 µL PBS/well [99] for each soluble fraction: i) kDNA+ chicken heart; ii) kDNA- chicken heart; and iii) *T. cruzi* epimastigote antigen. The antigen in the well was incubated for 18 h at 4°C in a moist chamber. ELISA was performed as described [99]. Briefly, the alkaline phosphatase-conjugate (Sigma) anti-chicken immunoglobulin were used at 1∶1000 dilution in PBS, pH 7.4 containing 2% nonfat milk. The color reaction was developed by the substrate p-nitrophenyl phosphate (pNPP, 50 µL/well) dissolved in diethanolamine buffer, pH 7.9 (Sigma). Test and control serum assays were run in triplicate, and the mean ODs ± standard deviation were recorded. A serum dilution yielding absorbance 0.150 or above was considered to be a positive reaction. This cut-off determined in standard serum samples was used to separate positive from negative controls. The ELISA test for the human *T. cruzi* antibody was conducted as described [99].

### Heart protein extraction and 2DE identification

Heart tissue was cut into 1 mm pieces, washed with PBS containing a protease inhibitor cocktail (5 mM EDTA, 100 µM PMSF, 100 µM TLCK, 100 µM TPCK, 1 µM pepstatin A, 100 µM leupeptin) at 4°C, and centrifuged three times at 10,000 rpm with supernatant discarding. Lysis buffer (7 M urea, 2 M thiourea, 2% Triton X-100, 1% DTT and the protease inhibitor cocktail) was then added in the proportion of 6 mL to 1 g of tissue, followed by vortexing for 2 min and sonication in a refrigerated bath for 2 min three times. The material was left at 4°C for 20 min and finally centrifuged at 14,000 rpm for 15 min. The supernatant (heart extract) was submitted to protein quantitation using the PlusOne 2D Quant Kit (GE Life Sciences). The protein profiles of heart extracts (100 µg) were analyzed by 2DE [100]. Peptide mass fingerprinting [63] was used for protein identification via an Autoflex II mass spectrometer (Bruker Daltonics) and the Mascot software (Matrix Science).

### Labeling chicken immune cells with fluorochromes

**Figure 1:**
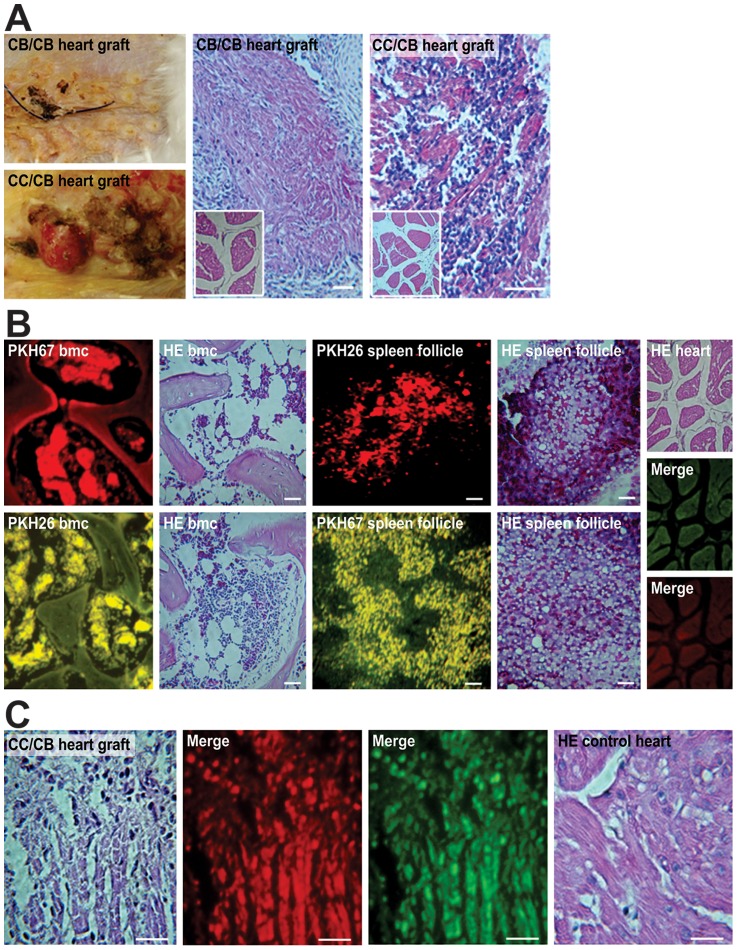
Immune tolerance and its disruption during the rejection of incompatible tissue grafts. A) The acceptance of compatible heart grafts from syngeneic chickens. Left, healed surgical skin incision and reporter heart acceptance (top) and skin ulcer and reporter heart rejection (bottom). The compatible healthy myocardium was cuffed by loose fibrous tissue (middle) at 17 days. The incompatible heart exhibited extensive necrosis (right) at 14 days. Inset, control heart prior to grafting. B) Fluorochrome-labeled immune cells in congenic Prague chickens. Top row, kDNA- normal BMC and spleen lymphoid follicle histology. Bottom row, kDNA+ BMC and splenic lymphoid follicles exhibiting hypercellularity. Right column, normal histology of one-day-old chick myocardium. Staining: H-E, PkH26, and PkH67. C) Rejection of incompatible heart grafts in congenic chickens. The donor CC graft was acutely rejected by the recipient CB immune cells within 11 days. Left, H-E; middle, red and green labels. Right, one-day-old heart graft displaying normal histology. Bars, 10 µm. Three independent experiments were performed.

The NCs and the kDNA^+^ B^12^/B^12^ chickens were used. Twenty milliliters of venous blood collected in 20 units of heparin was layered on top of Ficoll-Hypaque (GE Healthcare) and centrifuged at 3500×*g* for 30 min at room temperature. The plasma in the supernatant was removed and centrifuged at 4000×*g* for 10 min to obtain the platelet-poor plasma fraction. The white blood cells on the interface were collected and washed thrice in PBS, pH 7.4, by centrifugation at 1000×*g* for 10 min. A total of 10×10^6^ mononuclear cells were stained with 1×10^−6^ dilution of the fluorochrome (10 µL PKH +10 mL Sigma diluents C). After incubation for 10 min at room temperature, the cells were washed five times in PBS. The pellet of the last centrifugation was suspended in the platelet-poor plasma fraction. For *in vivo* labeling of BMCs from kDNA+ and NC chickens, a series of injections of 1×10^−6^ M of fluorochrome PKH26 red dye (1st and 3rd doses) and PKH67 green dye (2nd and 4th doses) were administered two weeks apart in the wing vein of 3-month-old chickens. Sections of bone marrow, spleen, and heart of adult birds were processed for histopathology, and the distribution of the labeled immune cells in the tissue was recorded. The standardization procedure showed the staining of BMC and of lymphoid spleen cells with the fluorochromes PKH26 red and PKH67green. The bone, muscle, and connective tissues revealed UV fluorescence, often seen in unstained sections. The double labeling allowed confirmation of specific immune cells staining, and merge image collection (Fig. 1).

### Tissue grafting

Skin grafts [16] were performed between birds with CB (B^12^/B^12^) haplotypes and between birds with CC (B^4/^B^4^) haplotypes to show the integrity of the tolerance mechanism in the absence of graft rejection. Skin grafts were also performed between chickens with different haplotypes (from CB to CC and from CC to CB) to show the active recognition of non-self mechanism in the presence of incompatible MHC-BL genes. The grafts were inspected daily for evaluation of the surgical incision site and removed at day 11, 14, or 17 post-implant.

The tolerance mechanism regulated by the MHC-BL genes was further assessed through the reporter heart graft experiment. One-day-old chicks hatched from healthy NC or from kDNA+ chicken donors received a graft of the NC or kDNA+ adult chicken receptor. The chick's heart was excised and placed into a Petri dish with cold PBS, pH 7.4. The heart's blood was completely washed after longitudinal sections were cut and two changes of cold PBS were made [100]–[102]. One half of the donor heart was grafted in the subcutaneous tissue at the interscapular region of the receptor chicken, at the site where feathers were plucked under anesthesia. The skin wound was closed with stitches, and the wound was inspected daily. At day 11, 14, or 17 post-graft, the heart implant was excised, fixed in formalin, embedded in paraffin, and cut into 4-µm-thick sections, which were hematoxylin and eosin (H-E)-stained for histopathology or used unstained for immunohistochemistry study. The second half of the donor heart was fixed in formalin and processed for histopathology and immunohistochemistry study.

The reporter heart grafting was conducted in kDNA+ and in healthy NCs of CB chickens with or without fluorochrome labeling, in order to phenotype the immune cells involved in the pattern of graft rejection or non-rejection.

### Transfer of BMCs to recipients

The quantitation of the number of blast cells in BMC aspirates from chicken femur bone was determined accordingly with morphologic criteria [103]–[105]. The cells were collected in 5 IU sodium heparin, and 10 µl of the aspirate was immediately smeared on two glass slides, fixed in methanol and H-E-stained. The microscopic exam revealed total count 14.1±2.4×10^4^ blasts/ml, and 3±1 ml of BMC aspirate was injected in recipient chickens through an ulnar vein. Groups of eight-month-old congenic chickens of the Prague line received folate inhibitor (Myleran, 14 mg/kg diluted in 10 ml PBS) through a cannula *per os* and cytostatic methotrexate (150 mg/kg) through injections in the wing vein [106]–[109]. Two days after the ablation of the bone marrow with the drugs, the chickens received marrow cells aspirates from the femoral bone of NC or kDNA+ congenic chickens. The NC and kDNA+ groups of chickens that underwent bone marrow ablation were employed in the transfer of BMCs through injection into the femurs of chickens in separate groups: i) kDNA^+^ chickens that received bone marrow aspirates from NC; ii) healthy NCs that received bone marrow aspirates from kDNA+ chickens; iii) healthy NCs that received BMC aspirates from NC; and iv) mock control NC chickens that did not undergo ablation but received BMCs from counterpart controls. Thirty days after bone marrow transplantation, the chickens had a one-day-old kDNA+ chick's heart implanted in the subcutaneous pouch at the interscapular region. The graft heart was removed for histological analysis at set times, and 4-µm-thick histological sections were made unstained for microscopic examination under UV filters, or H-E-stained for bright field microscopy.

### Pathology

The pathology was examined in the tissue of F0 chickens that died in the course of the study and in the tissue of F1, F2 and F3 progeny sacrificed at 10 to 12 months of age. The sections of the heart, skeletal muscle, large bowel, liver, kidney, and spleen tissues were fixed in 10% buffered formalin, embedded in paraffin, sectioned at 4 µm thick in a rotation microtome, and stained with H-E for histopathological study. The unstained tissues from chickens that had been injected with the fluorochromes were examined under an Olympus BX51 under UV light with filters for the red dye PKH26 (551 and 567nm) and for the green dye PKH67 (490 and 502 nm), and images were collected simultaneously.

### Immunohistochemistry

The tissues from NC, from kDNA+ chickens, and from chick's heart graft haves, which were fixed in formalin and embedded in paraffin, were cut in a microtome. The unstained sections were subjected to three consecutive baths in xylene and hydration in an alcohol gradient (100%, 90%, 80% and 70%). Non-specific binding was blocked with 10% nonfat milk powder in PBS, pH 7.4 for 45 min at 37°C in a moist chamber. The tissue sections rinsed in PBS were incubated with a biotinylated mouse anti-chicken monoclonal antibody (Mab) for 2 h at 37°C. After three washes in PBS, the slides were incubated for 30 min with a Cy3-conjugated mouse anti-biotin Mab (Sigma). After incubation, the slides washed twice in PBS were counterstained with Harris hematoxylin for 2 min, dehydrated in alcohol, treated with xylene and mounted with a glass cover. Quality control was ensured by omission of the primary antibody for the control and for the test exam. The reading was performed under an Olympus BX51 microscope under UV under suitable filter wavelengths for DAPI, FITC and CY3. The photograph was captured by an Olympus DP76-U-TV0-63XC micro camera equipped with the cellSens Dimension image analysis program.

We used eight primary mouse Mabs against chicken immune system cell surface proteins (Southern Biotechnology) used at 1/20 dilution, as described in the manufacturer's protocol: 1) anti-CD8-α specific for 34-kDa alpha-chain T cells, which is expressed in 80% of thymocytes, 15% of peripheral blood mononuclear cells and 50% of spleen cells; 2) anti-CD8-β, expressed in approximately 45% of peripheral blood mononuclear cells and 50% of spleen cells; 3) anti-TCRγδ specific for T CD8^+^ γδ cells; 4) anti-TCRvβ1, which reacts with approximately 40–50% of peripheral blood mononuclear cells and 40% of splenocytes; 5) anti-TCRvβ2, which reacts with approximately 9% of thymocytes, 15–20% of peripheral blood mononuclear cells and 13% of splenocytes; 6) anti-CD4, which is a glycoprotein expressed on approximately 70% of thymocytes, 10% of spleen cells and 45% of peripheral blood lymphocytes; 7) anti-CD28; and 8) anti-CD45, which is a transmembrane glycoprotein present on the surface of T and B cells. We also used CY3-labeled anti-biotin, at 1/200 dilution, and **a**nnexin V-FITC° to produce enhanced fluorescence signals with high photostability [110]–[112] for the rapid detection of apoptosis in living cells via fluorescence microscopy (Sigma-Aldrich, USA). The annexins bind to phosphatidyl serine, which flips to the outer leaflet of the phospholipid bylayer membrane on apoptotic cells, and allows its identification. The greenish cytoplasm of apoptotic cells was identified by the annexin V-FITC antibody, whereas the nucleus was stained red by the propidium iodide.

### Semi-quantitative analysis of the inflammatory pathology

The mononuclear inflammatory cells that infiltrates the heart, skeletal muscle, large bowel, liver, kidney, and spleen of chickens that died in the course of the study, as well as in the F1, F2 and F3 progeny sacrificed at 10 to12 months of age, were recorded. The intensity of the inflammatory infiltrate in the tissues was evaluated by the minimal rejection unit (MRU), which defines the lysis of single target host cell by the immune lymphocytes [10]. The quantity of MRUs in four 2×1 cm sections indicated the intensity of the inflammatory infiltrates (+ MRU to ++++ diffuse MRUs). The H-E-stained sections of the myocardium from F0 that died in the course of the experiments and of the progeny (F1 to F3) that were sacrificed at 10-to-12 months of age were examined under a bright field microscope, and the intensity of the inflammatory lesions was estimated.

### Bioinformatic analyses

The chimera sequences were subjected to searches in BLASTn and BLASTx (http://www.ncbi.nlm.nih.gov/genome/seq/BlastGen/BlastGen.cgi?taxid=9031). The *T. cruzi* sequences were analyzed at the *Database Others (Nucleotide collection)* and *Somewhat similar sequences*. The chicken DNA sequence was subjected to *Database Gallus gallus genome reference* and *Somewhat similar* analyses. CLUSTALW alignments were performed, and statistical significance (p<0.001) was determined for the scores (e-values) recorded (Tables S2 and S3). The chimeras' repetitive DNA sequences were identified by the CENSOR-GIRI software (http://girinst.org/censor/index.php). The KISS search tool was employed to identify potential gRNAs in the kDNA sequences as well as a work bench for RNA editing analysis in kinetoplastids with the aid of WU-Blastn-modified-matrix http://www.plosntds.org/article/info%3Adoi%2F10.1371%2Fjournal.pntd.0001000 - pntd.0001000-Lopez1(http://www.biomedcentral.com/content/supplementary/1471-2164-8-133-s1.fas). Student's *t* test was used to detect differences between heart/body weight indexes, and the Kolmogorov-Smirnov test was used to compare mortality ratios between groups of chickens hatched from *T. cruzi*-inoculated eggs and from mock controls. The results were expressed as the means ± standard deviation. p<0.05 was considered statistically significant. This analysis included NCs and kDNA+ chickens that underwent natural death. The survival time (months) and the size (weight) of the heart of each chicken were recorded. The heart weight (g) and the chicken body weight (kg) were used to calculate the heart weight index: HWI  =  heart weight (g)/body weight (kg).

## Results

### Graft rejection/survival in the experimental system of the Prague congenic lines

The congenic lines CB (B^12^/B^12^) and CC (B^4^/B^4^) were used [91]-[95]. These lines are genetically identical except for the MHC class I (B-F) and class II (B-L), and for other multigene families outside the MHC BF-BL region, which appears to determine effective antigen presentation during mature immune responses [94], [95] and acute rejection of grafts. These congenic lines were used to demonstrate the heart graft rejection-dependent genotype modifications in the chicken genome following *T. cruzi* minicircle kDNA mutagenesis.

In the skin graft experiment between CB (B^12^/B^12^) and CC (B^4^/B^4^) chickens we showed the integrity of the tolerance mechanism in the absence of graft rejection. Skin grafts were also performed between chickens with different haplotypes (from CB to CC and from CC to CB) to show the disruption of the tolerance mechanism in the presence of incompatible BF-BL region and other gene families outside the MHC complex [94], [95]. The results demonstrated that skin grafts between MHC-compatible chickens (within a particular syngeneic line) were accepted permanently, whereas grafts exchanged between the CB and CC chickens were acutely rejected within 11 days.

Further experiments were performed to evaluate heart tissue grafting in groups of five syngeneic chickens. One half of each one-day-old NC chicken heart was inserted into the inter-scapular subcutaneous tissue of an adult recipient. Three groups of independent experiments were performed for the following transplants: i) between compatible CB (B^12^/B^12^) chickens; ii) between compatible CC (B^4^/B^4^) chickens; iii) between incompatible CB donors and CC recipients; and iv) between incompatible CC donors and CB recipients. At different time points, the heart grafts were removed and subjected to histopathological evaluation. In combinations i and ii, the chickens with compatible MHC-B haplotypes did not reject the heart grafts. Microscopy revealed edema and vascular engorgement at 8 days, which was followed by loose connective tissue cuffing of the heart graft and no rejection of the donor myocardium at the 17th day. In contrast, the incompatible grafts (groups iii and iv) underwent rejection and inflammation-induced necrosis by the 14th day. The lymphocyte infiltrates in the reporter heart provided evidence of the disruption of immune tolerance and rejection. The second half of the heart sections, derived from control donors, exhibited normal histology. The results are summarized in Figs. 1A, 1B, and 1C.

### Modification of the CB (B^12^/B^12^) and CC (B^4^/B^4^) chicken genomes by integrating kDNA minicircles from *Trypanosoma cruzi*

**Figure 2:**
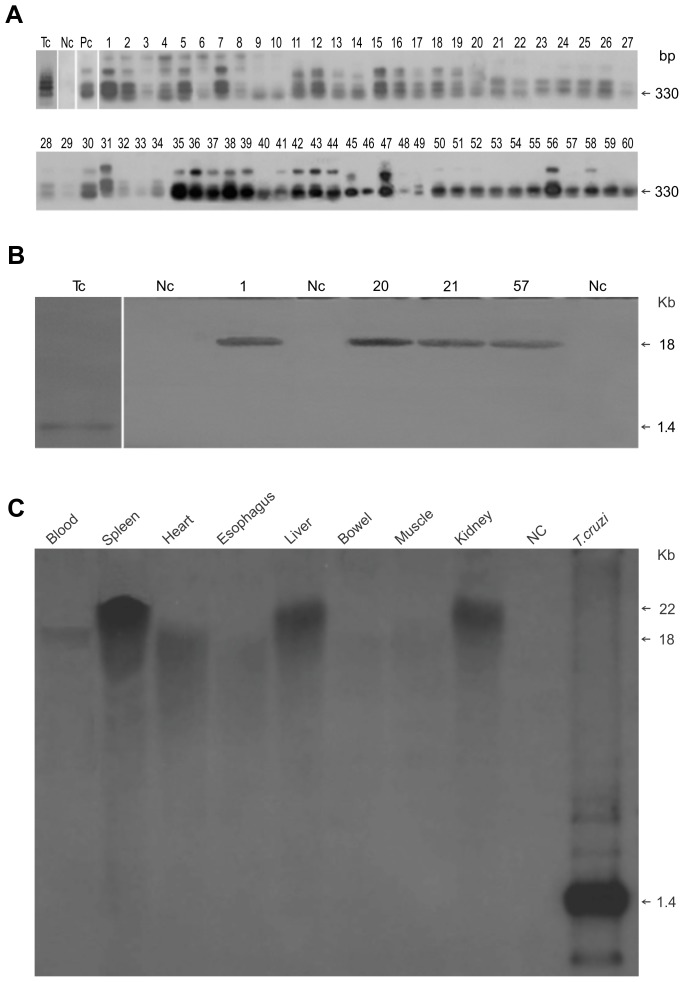
Modification of the CB chicken genome by the integration of kDNA minicircles from *Trypanosoma cruzi*. A) PCR amplification of the minicircle sequences in the genomes of (kDNA+) chickens hatched from *T. cruzi*-inoculated eggs. Tc, *T. cruzi*; Nc, negative control; Pc, positive control. Numbers 1 to 60, kDNA± F0 chickens. B) Southern hybridization of the *Eco*RI digests of blood mononuclear cell's DNA from kDNA+ chickens, demonstrating the 18-kb band. C) Southern hybridization of the *Eco*R1 digests of somatic cells from kDNA+ F2 rooster number 16, showing 18-to-20 kb bands. Nc, negative control. The *T. cruzi* positive control reveals a main 1.4 kDNA minicircle band.

This system of syngeneic lines described above was used to evaluate the noxious (auto)immunity induced in syngeneic chickens subjected to genomic modifications by the integration of *T. cruzi* kDNA minicircles following the inoculation of 100 *T. cruzi* trypomastigotes through a hole in the air chamber shell [10], [66]. The measurement of parasite template DNA in the chicken genome was conducted by PCR, with primer sets specific for the parasite kDNA and nDNA [96], [97]. These assays revealed that the mitochondrial kDNA minicircle sequences were retained in the genomes of CB (B^12^/B^12^) and CC (B^4^/B^4^) chicks hatched from *T. cruzi-*inoculated eggs (Fig. 2A). In view of the similar results obtained with these syngeneic lines, we chose the CB (B^12^/B^12^) line, which grew faster than the CC (B^4^/B^4^) line. The mock control chickens were nDNA- and kDNA-free. The specificity of the primers for the *T. cruzi* kDNA 330-bp and nDNA 188-bp sequences was demonstrated by the absence of the amplification of *Leishmania braziliensis* DNA [10], [66]. The refractory nature of the CB chickens to *T. cruzi* infection was demonstrated by PCR, which had a sensitivity of 10 fg, 24-fold less than the total nDNA in a single *T. cruzi* trypomastigote [67].

Southern blot assays documented the integration of the kDNA minicircle sequences into the chickens' genomes. *Eco*RI digests of the DNA from the chicks hatched from *T. cruzi-*inoculated eggs and from mock control eggs were probed with radiolabeled wild-type kDNA minicircle sequences, revealing 18 kb band in the kDNA-mutated blood mononuclear cells, and 18 to 20 kb bands consistent with exogenous DNA integration in the chicken genome (Fig. 2B and 2C). Southern blotting also revealed the absence of an nDNA band formed by the hybridization of the NC chicken DNA templates with the radiolabeled 188-nt DNA probe. Obtaining 18 and 20 kb bands instead of several size small bands consistent with a generalized mutagenesis stem from the repetitive sequences of TEs present in 164 out of 200 (82%) integration spots, which shared microhomology with the minicircle CSBs present in CRs of the kDNA radio labeled probe [10], [66] sequence smeared through the electrophoresis gel-transferred nylon membrane. These shared microhomology repeats dispersed in the chicken genome may explain the presence of 18 and 20 kb bands after overnight film exposure to a radio labeled membrane, and heavy smears instead of multiple small bands after film exposure to radiograph for 72 hours or more (Fig. 2B and 2C). Similar findings were shown in previous papers [10], [66].

### Labeling the isogeneic chicken immune cells with fluorochromes

CB chickens were used for immune cell labeling experiments to evaluate both kDNA+ chickens hatched from *T. cruzi-*inoculated eggs and syngeneic NCs that never had contact with *T. cruzi*. The PkH26 and the PkH67 fluorescent cell linkers (Sigma-Aldrich) were used to stably incorporate a dye with long aliphatic tails into the lipid receptors to labeling membrane of rapidly growing cells. These fluorochomes have been used for labeling epithelial cells in the gut [113], leukemia cells [114], lymphocytes [115], epithelial and mesenchymal stem cells [116], and cord blood T lymphocytes [117] for *ex vivo* and *in vivo* cell proliferation analysis. The chickens in these groups received alternate injections of red (PkH26) or green (PkH67) fluorochromes intravenously every two weeks (see Methods). After 15 days, the double-fluorochrome-labeled chickens were euthanized, and sections of the bone marrow, spleen, and heart were subjected to microscopic analysis under bright-field and UV illumination, using the appropriate wavelength filters. The bone marrow stem cells and blast-derived immune lymphocytes, which were stored in the spleen and often infiltrated the heart, exhibited intense fluorescence, unlike the bone, connective tissue, smooth and striated muscles, which exhibited faint, natural fluorescence. The fluorochrome-labeled cells stored in the spleen follicles migrated freely into the blood vessels. H-E-stained sections from kDNA+-derived bone marrow often exhibited hyperplasia, in contrast to the normal histology in the NC chickens. In some kDNA+ spleens, the lymphoid follicles contained approximately twofold brighter immune cells compared with the NC chicken cells (Fig. 1B).

### Absence of Chagas heart gross pathology in NCs subjected to passive transfer of blood immune effector cells from syngeneic kDNA+ chickens

**Figure 3:**
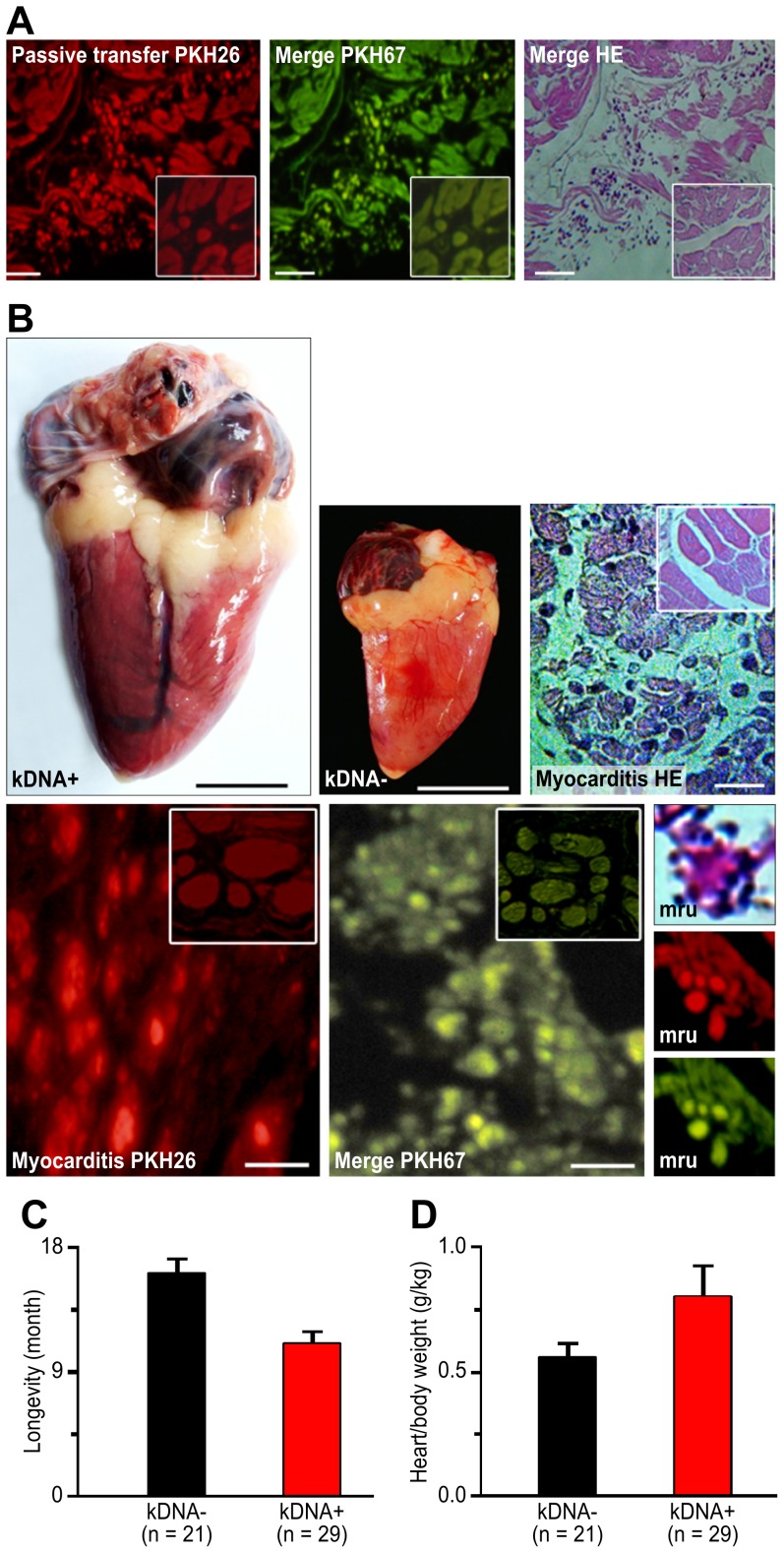
Gross and microscopic pathology resulting from the modification of the syngeneic chicken genome by the kDNA mutations from *Trypanosoma cruzi* minicircle sequences. A) Passive transfer of fluorochrome-labeled immune cells to syngeneic NC chickens. The kDNA+ immune cells were observed in the myocardium of the recipient. Insets demonstrate the absence of NC effector cells in the myocardium of the NC recipient. Bars, 5 µm. B) Production of Chagas-like heart pathology in syngeneic kDNA+ chickens hatched from *T. cruzi-*inoculated eggs. The kDNA+ heart weight 21.6 g, and the kDNA- heart weight 9.6 g. Severe myocarditis was present in the kDNA+ heart (H-E, right). The myocarditis was depicted by fluorochrome-labeled sections (bottom row), and typical MRUs are presented in the right column. Bars, 10 µm. C) Survival (months) of kDNA+ and kDNA- NC chickens. Differences are statistically significant (p<0.005). D) Heart weight/body weight indexes obtained upon death of kDNA+ and kDNA- F0 chickens. Differences are statistically significant (p<0.05%). Data of group ii chickens are representative of three independent experiments. Bars: Gross, 1 cm; micro, 10 µm.

To demonstrate whether it was possible to transfer the lesions observed in the CB kDNA+ chickens, we performed passive transfer of blood mononuclear cells from these chickens to eight-month-old syngeneic, NC recipient chickens. In the test group, each of five NC chickens received injections of 10^7^/ml kDNA+ blood mononuclear cells that were *in vitro*-labeled with red and green fluorochromes, administered one week apart through an ulnar vein. In the controls, the same protocol was used for the transfer of the fluorochrome-labeled mononuclear cells from NC to kDNA+ chickens. Fifteen days after the third weekly injection, the chickens from both groups were euthanized, and their tissues were examined. Histopathology revealed that the kDNA+ donor double-labeled immune lymphocytes formed scarce, small infiltrates in the hearts of the NC recipient chickens (Fig. 3A), but there was no gross pathology. In contrast, passive transfer of fluorochrome-labeled mononuclear cells from NC birds did not infiltrate the recipient chickens' hearts.

### Assessment of pathology

The kDNA+ chickens died naturally during the course of the experiments, and gross and microscopic pathology was recorded (Fig. 3B). The microscopic examination of four 2 × 1 cm H-E-stained sections from the F0 chickens that underwent natural death, consistently revealed: 15 chickens (25%) had evidence of an MRU (+), herein defined as single target heart cell lysis by lymphocytes; seven chickens (12%) exhibited a few (≤6) nearby MRUs (++) and lysis of target cells; six chickens (10%) exhibited confluent MRUs (≥6) (+++) and lysis of multiple heart fibers; three chickens (5%) exhibited diffuse myocarditis (++++) and lysis; and 29 chickens (48%) exhibited normal heart (-) histology. Body weight and heart weight were recorded. The development of heart pathology over time resulted in the early death of the kDNA+ chickens at 13.2±7 months, whereas the NC chickens survived 19.2±8 months; this difference was statistically significant (Fig. 3C). The kDNA+ chickens developed gross cardiomegaly-induced shortness of breath, cyanosis, hydrothorax, and ascites, which were correlated with myocarditis and with the rejection of the heart fibers. Three progeny (F1, F2 and F3) showed muscle weakness. The heart weight (g) and body weight (kg) of the kDNA+ and NC chickens were used to generate indices, which exhibited significant differences (Fig. 3D). The collective data revealed the kDNA+ chickens' loss of immune tolerance and rejection of compatible heart grafts. The kDNA- chickens, with their absence of heart pathology, exhibited no clinical manifestations.

**Figure 4:**
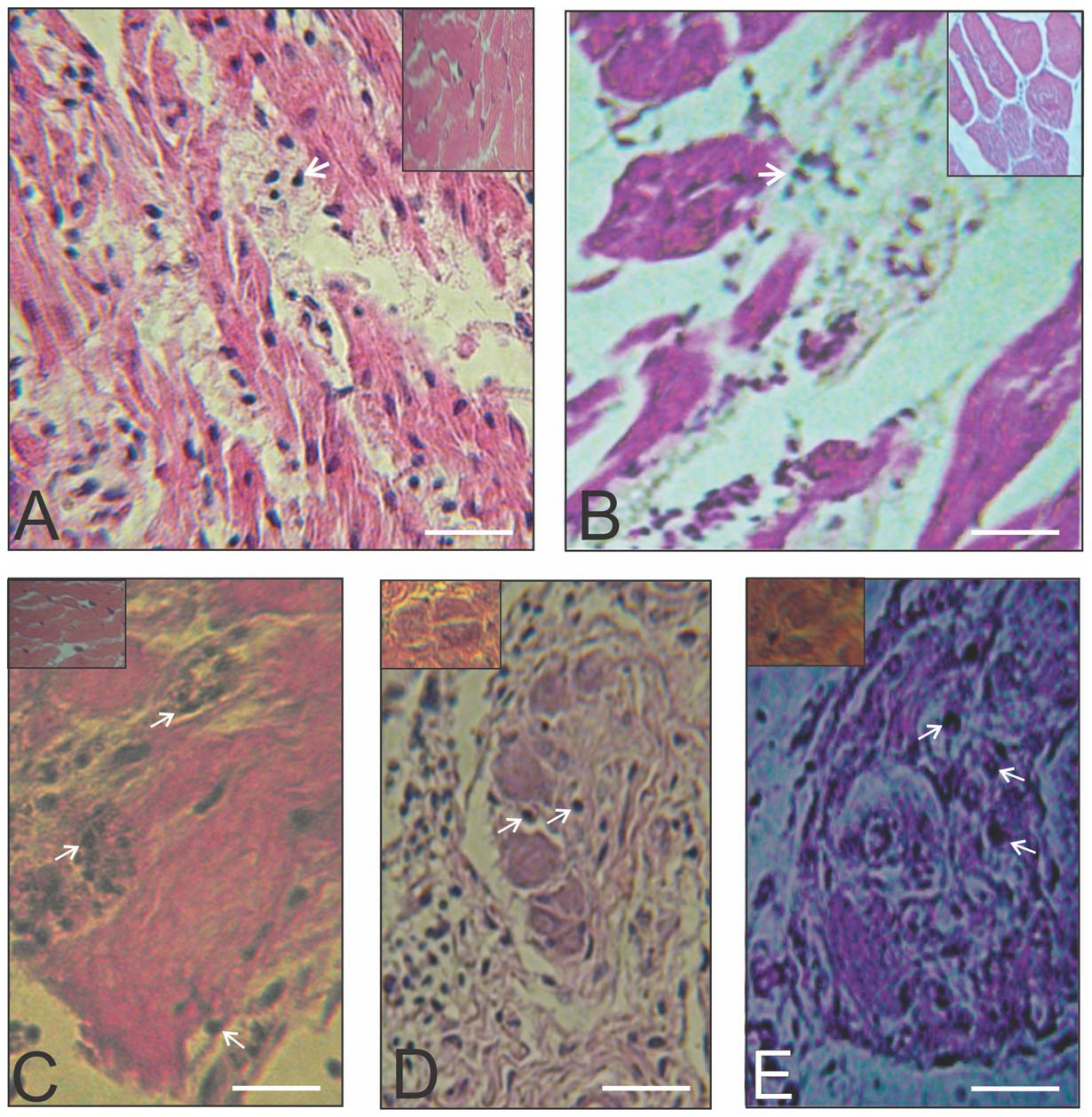
Microscopic pathology in the heart and skeletal muscles of a dystrophin gene kDNA-mutated rooster, showing inflammatory infiltrates and lysis of striated muscle and of parasympathetic neurons. A) and B) Heart (left) and skeletal muscle (right) sections showing vacuolation of fibers and inflammatory infiltrates (arrows) in rooster 24 with mutation (HG531472) in the dystrophin gene. C) Inflammatory infiltrations and target striated muscle cell lysis in a chicken progeny. D and E) Inflammatory lymphocytes infiltrates (arrows) parasympathetic ganglia of the large bowel (middle) and of the heart (right), and neuronolysis. Bars, 10 µm.

Three chickens with mutations in the dystrophin gene (birds 3, 15, and 24, S2 Table) revealed foam cells and inflammatory infiltrates in the heart and skeletal muscles (Fig. 4A and 4B). Additionally, inflammatory lesions alone were often of lesser intensity in the progeny than those shown in the F0 chickens. The heart sections revealed inflammatory infiltrates (+ to +++) and target cell lysis in 35 (25%) progeny chickens (F1, 12; F2, 13; F3,10). The skeletal muscle sections of 15 (10.6%) progeny chickens presented the inflammatory infiltrates (Fig. 4C). The parasympathetic nervous system showed inflammatory infiltrates (+ to +++) and occasional neuronal cells lysis (Fig. 4D and 4E) in 10 (7%) cases. Liver and kidney showed an absence of inflammatory infiltrates. The NC chickens revealed normal histology in the tissues and organs examined. Taken together, these findings reveal that kDNA+ chickens bear the potential to initiate autoimmune inflammatory lesions that appear to recrudesce over time.

### The inhibition and transfer of heart pathology by compatible BMC transplantation

**Figure 5:**
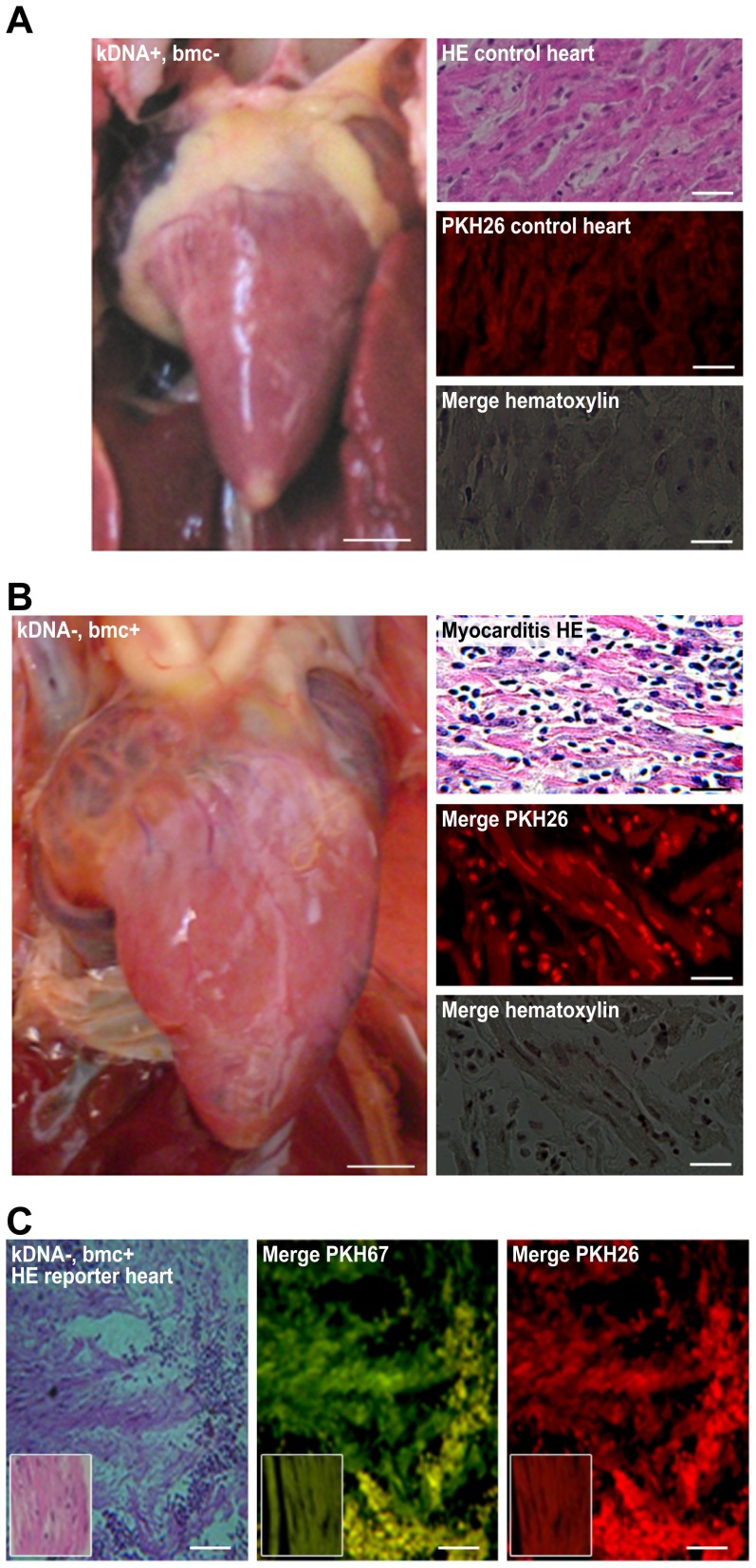
Inhibition and transfer of heart pathology by bone marrow transplantation. A) Inhibition of heart lesions by the replacement of kDNA+ sick BMCs with healthy kDNA- BMCs in syngeneic chickens. Heart (10.6 g) from a kDNA+ chicken receiving BMCs from a kDNA- donor revealing normal histology. B) Transfer of the heart lesion by the transplantation of sick BMCs from kDNA+ to kDNA- chickens. The heart weighed 26.1 g, and the myocardium exhibited infiltration by effectors lymphocytes and lysis of target cells. C) The reporter heart graft pathology in a kDNA NC chicken receiving BMCs from kDNA+ chicken. The insets present one-day-old normal heart histology before grafting. Data are representative of three independent experiments. Bars: gross, 1 cm; micro, 10 µm.

The pathologic findings in the kDNA+ progeny F1, F2 and F3 (Fig. 5), which were euthanized at 10 to 12 months of age, was documented. The production of small inflammatory infiltrates in the heart of NC by passive transfer of blood mononuclear cells from kDNA+ chickens revealed an inherent heart homing activity by immune lymphocytes. This finding led us to conduct transfer experiments with growing BMC-derived lymphoblasts, aiming at the inhibition of the Chagas-like heart disease. The inhibition of the pathology in the hearts of adult kDNA+ chickens (group *i*) or its transfer to NC chickens (groups ii and iii) through BMC transplantation was evaluated in three independent experimental groups. The chickens from groups i, ii, and iii, each with five birds, had their BMCs destroyed by injections of cytostatic and anti-folate drugs (see Materials). After two days, the kDNA+ chickens from group i received BMCs from NCs, the NC chickens from group ii received BMCs from kDNA+ chickens, and the NC chickens from group iii received BMCs from NC chickens. In group i, the kDNA+ chickens that received NC healthy BMCs exhibited no myocarditis and no heart rejection (Fig. 5A). In group ii, the NC chickens that received BMCs from kDNA+ chickens exhibited myocarditis and heart rejection (Fig. 5B). In group iii, the NC chickens that received BMCs from NC counterparts were completely healthy after six months.

### Reporter graft monitoring of the inhibition of heart pathology

To assess the *in vivo* protective effects of healthy BMC transplantation into kDNA+ chickens, we used the syngeneic CB chicken model system to monitor the pathology through reporter heart grafting. One month after the transplantation of healthy NC marrow cells into the kDNA+ chickens that had their BMC destroyed with drugs, we grafted a syngeneic reporter heart to evaluate whether it was possible to reconstitute the chickens' immune tolerance. We observed in triplicate groups of five chickens that the reporter heart grafts were accepted and that the healthy grafts became encapsulated by fibrous tissue by day 17 (Fig. 3C). In contrast, the compatible reporter hearts implanted into the NC chickens that received sick BMCs from kDNA+ donors were rejected by the recipients' immune cells. The healthy immune cells from NC chickens that replaced the kDNA+ chicken BMCs destroyed with drugs did not reject the syngeneic heart grafts. Control chickens (group ii) receiving the kDNA+ BMCs developed cardiomegaly and heart failure. The results presented in Fig. 5C illustrates group ii, second set experiments.

### Heritability of *Trypanosoma cruzi* kDNA minicircle sequences integrated into the chicken genome

**Figure 6:**
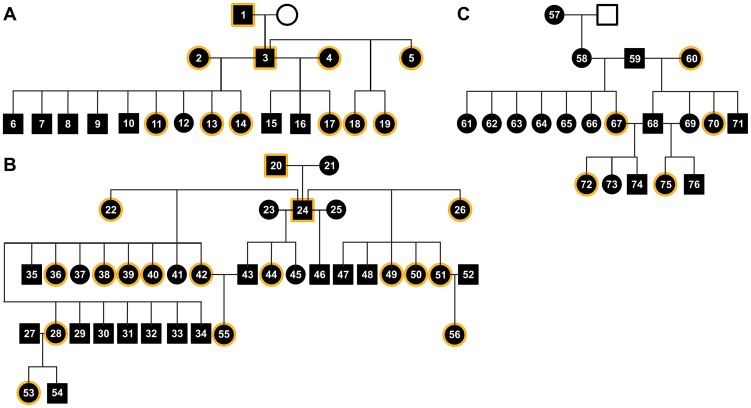
Heritability of the non-Mendelian kDNA mutations in CB chicken families A, B, and C.

We next sought to demonstrate clearly the familial predisposition and genetic susceptibility factors involved in the pathogenesis of heart rejection in syngeneic chickens. After breeding was conducted, we evaluated the pattern of inheritance of the *T. cruzi* kDNA minicircle sequences integrated into the parental genomes, which were vertically transferred to the progeny. The crossings produced the kDNA+ chicken families A, B, and C (Fig. 6).

We observed that the kDNA minicircle sequences integrated into the somatic cells from the parents and from the progeny (Fig. 2A, 2B and 2C). The somatic cells of 76 individuals from families A, B, and C consistently displayed evidence of the specific 330-bp kDNA minicircle band and its catamers. The crossings indicated that the kDNA minicircle sequences integrated into the genomes underwent vertical transfer to the descendants. These results confirm and extend previous knowledge of the heritability of kDNA mutations in an outbred chicken model system [10, 66, and 67].

**Figure 7:**
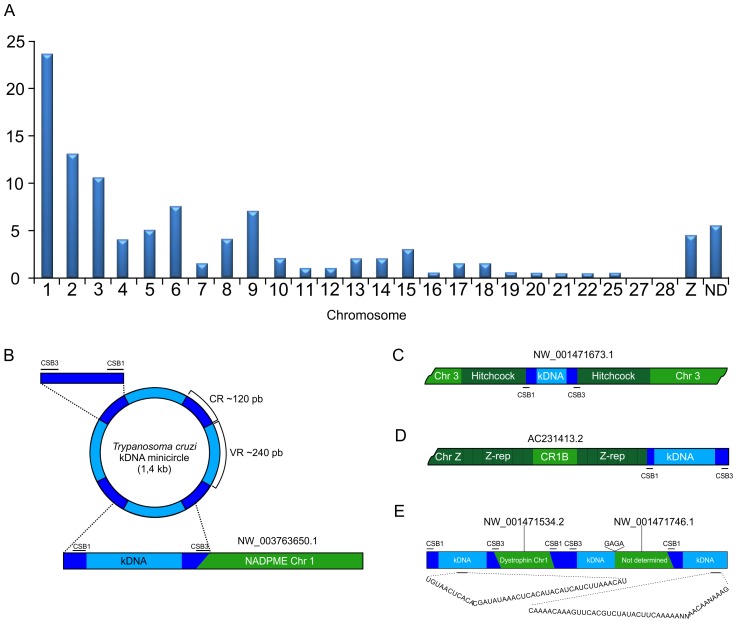
Mapping and schematic representations of the *T. cruzi* kDNA mutations in the chicken genome. A) Distribution of somatic cell mutations in the chromosomes of parental and progeny CB chickens. B) Schematic representation of the *T. cruzi* minicircle CSB3 and CSB1 mediating kDNA integration into the *NADPME* locus (HG531657) on chromosome 1. C) Minicircle kDNA (HG531412 and HG 531409) integration into a Hitchcock element at chromosome 3. D) Minicircle kDNA (HG531589) insertion into a repetitive element containing a CR1B element at chromosome Z. E) Three kDNA minicircle sequences hitchhike from the dystrophin locus at chromosome 1 (HG531399) to a second chromosome at an undetermined locus.

In this study, we combined the kDNA primer sets (S1 Table) with host primers, targeting a flanking host DNA sequence (FN599618) annealing to TEs [10, 66, and 67]. The somatic cell amplicon-derived clones exhibited evidence of host DNA-kDNA minicircle sequences, and revealed 200 non-redundant chimeras (S2 Table). The kDNA integration into the somatic cells was distributed in 23 of 38 autosomes and in the Z sex chromosome (HG531391 to HG531590) (Fig. 7A). Integration into chromosomes 1–5 and Z accounted for 58% of the total number of events. At the chimera junction site, the CA-rich microhomology revealed 8- to 25-nt short repeats. This result suggests that microhomology-mediated end joining was the primary mechanism mediating the host DNA-kDNA integration in the chickens, and that the microhomology hotspots might represent repetitive, truncated, reshuffled sequences, likely from TEs.

The longest host DNA-kDNA chimera sequence obtained was 1479 nt, and the kDNA minicircle maximal length reached 624 nt, with an average of 242±98 nt. The chimera minicircle alignments revealed an enormous diversity, explained by remarkable structural differences in their VRs. In contrast, almost perfect alignments among the kDNA CRs were obtained, and furthermore, the CSBs in the CRs revealed significant homology with highly conserved microhomology repeats dispersed in the chicken genome [10], [66]. At the kDNA integration site, the minicircle VR (average 230–250 bp) was usually flanked by truncated CR and VR fragments (50 to 100 bp) often observed amid CR segments, exhibiting high similarity scores (p<0.05). The variable topological patterns, consisting of host DNA flanking the kDNA minicircle at the junction site, suggested a variety of recombination events. To verify that the minicircle VR sequences originated from *T. cruzi*, we evaluated the sequences for the presence of gRNAs (S3 Table). The full complement of 113 gRNAs [68], [69] was predicted in the kDNA VR minicircle sequence chimeras in the chicken genome.

In the syngeneic chicken model, DNA integrations were frequently observed at locus NW_003763650.1, coding for the NADP-dependent mitochondrial malic enzyme (*NADPME*) [118]. Fifteen F1, F2, and F3 progeny chickens in families A, B, and C had kDNA minicircle integrations at the *NADPME* locus (Fig. 7B). A total of 30 kDNA mutations had minicircles inserted exactly at nt 90092 of the *NADPME* gene sequence on chromosome 1, and 15 of these kDNA mutations were obtained through template amplifications with gene-specific primer sets (S1 Table). All of these mutations resulted in an identical 1.7-kb *NADPME* sequence at locus NW_003763650.1 (S2 Table). In this series of kDNA mutations, some minicircle VRs were predicted to transcribe gRNAs (S3 Table). The kDNA integrations in retrotransposon CR1 sequences were distributed in the parental chickens' genomes (HG531593, locus NW*_*003763785.1; HG531594, locus NW_0037636687.1; HG 531596, locus NW_001471668.2) and in the progeny genomes (HG531644, locus BX640540.3).

The repetitive TEs, mainly non-LTR retrotransposon CR1 represented (82%) of chimera sequences kDNA-human DNA. In one instance, Hitchcock LTR retrotransposons (11%) were found (Fig. 7C). Additionally, the kDNA integrated into the CR1B segment (HG531589) present in a gene sequence (Fig. 7D), in which the host DNA coding region (AC231413.2) might represent an intron-derived degenerate TE. Eight independent events documented the hitchhiking of a kDNA minicircle to a second chromosomal location (Table 1 and Fig. 7E). The resultant mosaics can likely be explained by endogenous CR1 reverse transcriptase activity [10, 65,66, and 67].

**Table 1:** Hitchhiking *Trypanosoma cruzi* minicircle kDNA sequences integrated into the *Gallus gallus* genome.

Ave	EMBL Number	G. gallus DNA(^≠^)	kDNA span	*G. gallus* span	Chromosomes	kDNA Identity	*G. gallus* Identity	*Locus* ^(**)^
2	HG531398	SC	166 to 453	1 to 98	14	87%	95%	NW_003763931.1 (ND)
				99 to 174	5		97%	NW_003763785.1 (ND)
3	HG531399	SC	1 to 263	246 to 461	1	99%	98%	NW_001471534.2 (Dystrophin)
			459 to 600	597 to 742	ND	97%	99%	NW_001471746.1 (ND)
			740 to 835			85%		
3	HG531400	SC	1 to 149	140 to 305	ND	89%	98%	NW_001471746.1 (ND)
				288 to 503	1		97%	NW_001471534.2 (Dystrophin)
5	HG531425	SC	55 to 261	1 to 70	1	99%	89%	NW_001471556.1 (ND)
				253 to 379	ND		76%	NW_001479132.1 (ND)
58	HG531536	SC	345 to 636	1 to 86	15	70%	96%	NW_001471461.1 (ND)
				87 to 360	2		98%	NW_001471646.1 (ND)
22	HG531671	GC	278 to 533	1 to 277	12	93%	98%	NW_003763892.1 (ND)
			835 to 927	522 to 846	6 ^(*)^	98%	98%	NW_003763812.1 (ND)

(^≠^) SC, somatic cell; (*) CR1 non-LTR fragment at chromosome 6; (**) ND, not determined.

The kDNA integrations were often found in the CR1 retrotransposons of syngeneic chickens, as previously documented in outbred chickens [10], [66]. Interestingly, a series of kDNA integrations illustrated the enormous genetic diversity embodied in the mutation events. These chimeras exhibited almost perfect alignment (96%) with the host DNA, and the kDNA exhibited 86% identity. Additionally, the kDNA mutations at locus NW_001471534.2 of the pyruvate dehydrogenase kinase gene were present in the genomes of birds 19, 39, and 73, belonging to families A, B, and C, respectively. In all of these chickens, CLUSTALW analysis revealed perfect alignment of the gene sequence and almost perfect alignment of the kDNA minicircles (HG531663, HG 531708, and HG531758).

The topological differences among the kDNA sequences integrated at the chicken coding regions could be explained by the unlimited genetic differences among thousands of minicircles' VRs inserted into early embryonic stem cells. Therefore, a single *T. cruzi*-infected gonium has the potential to generate a variety of kDNA-mutated gametes. This variety explains why the *T. cruzi* mitochondrial kDNA minicircle transfers, which resulted in topological modifications, did not allow for the identification of siblings through sequence analyses. Moreover, the phylogenetic sequence analyses of the chimeras, which exhibited evidence of recombination, hitchhiking, and reshuffling, could not verify the parents of individuals.

The kDNA integrations into the chicken genome introduced new open reading frames (ORFs) with the potential for the translation of chimeric proteins (S4 Table). The study revealed putative ORFs, and the BLASTp algorithm demonstrated that among those, there were 89 hypothetical chimeric proteins, 62 of which bore no significant similarity to existing proteins.

### The absence of specific antibodies to *T. cruzi* and to heart tissue in kDNA-mutated syngeneic chickens

**Figure 8:**
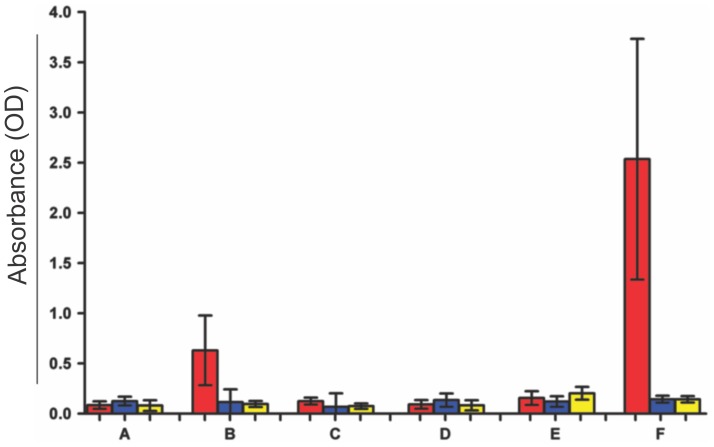
The absence of autoantibodies against *Trypanosoma cruzi* and against syngeneic soluble heart antigens in kDNA-mutated chickens. A) Pre-immune serum from NC chickens (n = 10). B) Immune serum from NC chickens receiving three subcutaneous injections of 10×10^7^ formalin-killed *T. cruzi* forms (n = 10). C) Serum from kDNA+ chicken (n = 25). D) Serum from kDNA-, NC chicken. E) Serum from human never exposured to *T. cruzi* (n = 10). F) Serum from Chagas patients with parasitological confirmation (n = 10). Columns: red, *T. cruzi* soluble antigen; blue, kDNA+ soluble chicken heart antigen; yellow, kDNA- soluble NC chicken heart antigen. Notice that significant titers of specific *T. cruzi* antibodies were detected in chickens immunized with formalin-killed parasite antigens, and that highly significant titers of *T. cruzi* antibodies were detected in human Chagas patients.

Next, we searched for autoantibodies that recognize specific anti-self heart antigen in cases of organ specific myocarditis and DMC [35], [49], [50]. These experiments shown in Fig. 8 revealed the absence of anti-heart antibodies in the kDNA+ as well in the kDNA- chickens. Additionally, Fig. 8 also revealed the absence of auto-antibodies against *T.cruzi* antigens in the kDNA+ chickens, thus suggesting these birds are immunetolerant to specific parasite antigens. Interestingly, chagasic patients (average 40±5 years of age) showing high titers of specific antibody to *T. cruzi* showed the absence of autoantibodies against chicken heart antigens. These results align with our early hypothesis that genetically driven autoimmune myocarditis in the transkingdom model of Chagas disease may be antigen independent [10].

The functional consequences emerging from the integration of exogenous DNA into the vertebrate genome seem to be directed at BMC progenitors of effector lymphocytes, causing an autoimmune reaction against heart tissue. Accordingly, the conventional antigen-driven autoimmune hypothesis was tested by comparing heart tissue extracts from kDNA+ and kDNA- chickens by two-dimensional gel electrophoresis (2DE). The protein profiles were similar in three independent experiments (S1 Fig.). Only a single protein spot in the kDNA+ sample was identified by peptide mass fingerprinting as C-reactive protein, which is overexpressed in inflammatory cardiomyopathy. We then searched for specific antibodies to heart cells and to *T. cruzi* neo-antigens in the serum of kDNA+ and NC chickens. ELISA revealed the absence of specific antibodies in the serum of kDNA+ and NC chickens (Fig. 8). Additionally, the results of control experiments revealed that the phenotype changes, transcribing putative chimeric ORFs (S4 Table), did not translate any recognizable neo-antigen. Altogether, in the absence of antibodies to *T. cruzi* and to heart antigens, these findings can be clearly explained by the kDNA+ chicken immune tolerance achieved during early embryonic development. In the absence of anti-self antibodies, the antigen-driven molecular mimicry failed in the parasite-free chicken model system of Chagas-like heart disease.

### Phenotypes of kDNA-mutated T cells associated with the rejection of histocompatible hearts

**Figure 9:**
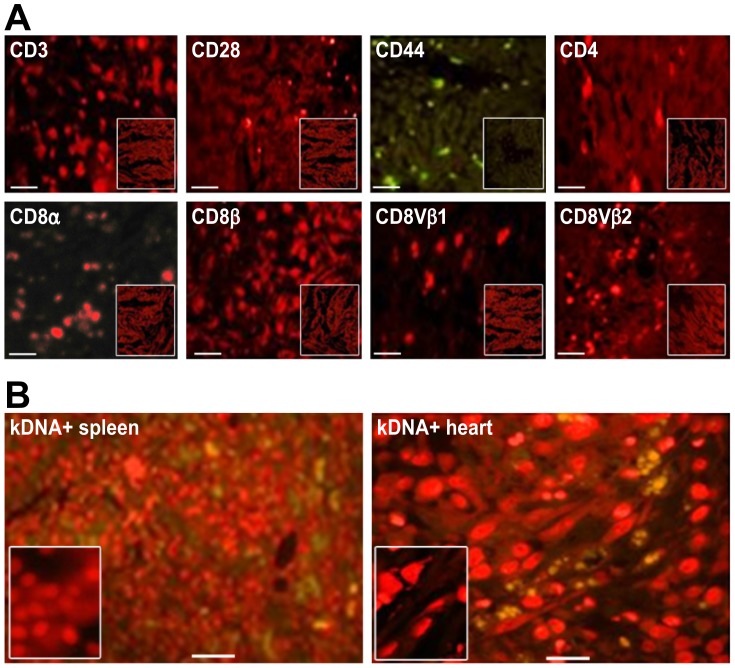
Phenotyping the immune effector lymphocytes involved in the rejection of the reporter heart graft and apoptosis. A) Heart sections from kDNA+ chickens sacrificed at 10 to12 months of age were subjected to analysis, showing effector T lymphocytes associated with syngeneic heart graft rejection in chickens with kDNA+ genome modifications, and immune cell phenotypes directly involved in the rejection. B) The effector immune cells underwent apoptosis in the spleen and myocardium of kDNA+ chickens. Tissue sections from kDNA+ and kDNA- chickens under semi-quantitative microscopy analysis revealed specific anti-annexin V-FITC monoclonal antibody-labeled apoptotic cells in the heart and spleen of kDNA+ chickens. Insets show kDNA-, NC chicken. Bars, 10 µm. Data are representative of three independent experiments.

Next, we tested our hypothesis that genotype-modified bone marrow-derived immune effector cells can attack and destroy syngeneic cells in the absence of the conventional antigen-dependent autoimmune reaction. To demonstrate the genetically driven immune rejection of the syngeneic heart, we used the kDNA+ progeny and NC chickens. We subcutaneously grafted syngeneic reporter hearts into sexually mature chickens. Groups of five syngeneic birds were used in triplicate experiments: *i*) kDNA+ chickens; *ii*) NC chickens; *iii*) kDNA+ chickens with drug-destroyed BMCs replaced by healthy NC marrow cells to inhibit heart pathology; *iv*) NC chickens undergoing transfer of the heart pathology, with the drug-destroyed healthy BMCs replaced by the kDNA+ marrow cells; and *v)* NC chickens with drug-destroyed BMCs replaced by NC marrow cells. In this study, we measured the clonal proliferation of the CD3^+^, CD28^+^, and CD45^+^ cell precursors of the thymus-dependent CD8α^+^ and CD8β^+^ effector cells expressing TCRγδ, vβ1 and vβ2 receptors, which infiltrated the adult hearts and the reporter heart grafts (Fig. 9). The results consistently revealed gross and microscopic effects similar to those present in the fluorochrome-labeled syngeneic chickens, whereby effector lymphocytes attacked and destroyed heart graft cells.

In light of the absence of anti-self heart antibodies in the kDNA+ CB chickens, we performed further experiments to elucidate the mechanism behind the disruption of immune tolerance to self-antigens, in the absence of chimeric ORF-putative neo-antigens, which would result in T cell-mediated rejection of the heart graft. Subcutaneous grafts consisting of a one-day-old kDNA+ syngeneic chick heart were not rejected by NC chickens. Subcutaneous grafts of kDNA+ reporter hearts into NC birds that had their BMCs destroyed by drugs and replaced by healthy marrow cells were not rejected either. These findings suggest that putative neo-antigens in the kDNA+ heart grafts either were not expressed or were not 'visible' to the immune system of the syngeneic chickens [119]. Taken together, the data suggest that the rejection of the hearts in kDNA+ chickens was an antigen-independent autoimmune phenomenon resulting from parasite-induced genotype modification and clonal proliferation of the effector T cells that attacked the heart. Thus, our findings confirm and extend our previous results of genetically driven, antigen-independent autoimmunity in a chicken model of human Chagas disease [10], [65], [66].

### Apoptosis of immune cells in kDNA+ chickens

The immune effector cells undergoing apoptosis in the hearts of kDNA+ chickens, as well as in the reporter heart grafts, were identified by fluorescence microscopy using FITC-conjugated annexin V antibody [110]–[112]. The apoptotic cells detected frequently in the hearts and spleens of adult kDNA+ chickens (Fig. 9B) were not observed in the tissues of control chickens. These results suggest that the apoptosis of the effector immune cells might be a down-regulation mechanism, preventing rejection of the target cells over a long time span in the genetically modified kDNA+ chickens, with disruption of immune tolerance and delaying of the onset of overt autoimmune heart disease.

## Discussion

In this study, we showed that Prague syngeneic chickens hatched from *T. cruzi-*inoculated eggs that retained the parasite mitochondrial kDNA (kDNA+) in the genome developed Chagas-like heart disease and died. Discrete inflammatory infiltrates were produced in the NC chickens subjected to passive transfer of fluorochrome-labeled blood mononuclear cells from kDNA+ chickens, demonstrating that kDNA+ chicken, but not NC blood mononuclear cells, had inherent heart-homing activity, even in the absence of clinical manifestations of heart pathology. The inflammatory infiltrates present in skeletal muscle and in parasympathetic ganglia suggested that somatic-mutated effectors' T-cells also have inherent homing activities directed to other targets in the body, although to a much lesser degree. In this regard, further studies are needed to explain the repertoire of T-cells homing specific targets in the body, and to define whether the cytotoxic reaction can be mediated by target cells MHC antigen recognition by putative receptors (Vβ-like) in the effector cells [54], [94], or the inherent homing activity can be driven by complex intragenomic pathways, such as the cAMP-dependent protein-kinase A signaling effectors cells and heart cells [36]. We hypothesize that biochemical pathways for intragenome signaling and cell-to-cell communication may associate somatic mutations at TEs repeat-motifs shared with the kDNA minicircle, so as to shed some light on the inherent homing of immune T-cells towards target tissues in Chagas disease.

Additionally, we used a grafting technique to monitor the development of lesions in the heart after labeling kDNA+ or NC chickens immune cells. The same technique was used to monitor the effects of BMC from kDNA+ or NC donors to recipients in which the BMCs were destroyed with drugs. This technique demonstrated that heart rejection can be improved by healthy BMC transplantation, thereby confirming our hypothesis that a chicken's immune tolerance is genetically controlled and that the disrupted immune tolerance exclusively observed in the kDNA+ chickens has severe physiopathological consequences that result in autoimmune graft rejection. Furthermore, we characterized the heritability of the kDNA mutation and the familiar genetic role of the autoimmune heart disease [10], [66], and we showed that genome modifications in the kDNA-mutated chickens disrupted their immune tolerance, which explains the heart rejection. Moreover, we showed that growing populations of lymphocytes (CD3^+^, CD28^+^, and CD45^+^), as well as the thymus-dependent CD8α^+^ and CD8β^+^ effector immune-competent cells that express TCRγδ, vβ1 and vβ2 receptors, displayed high selective homing activity towards the heart, and to a lesser extent towards skeletal muscle and parasympathetic nervous ganglia.

The kDNA-mutated T- lymphocytes that circulate in the blood and infiltrate the host tissues probably derived from pos-incubation epiblast, morula, and blastula stem cells by that early occasion when the *T. cruzi* infection gets inserted in the chicken embryo [10], [66]. In this regard every somatic cell in the body should have the somatic mutations. This is illustrated in Fig. 2C. The somatic mutations in the immune T cell phenotypes are suggested herein as main triggers of the autoimmune phenomenon's first signal, and subsequent massive tissue destruction can lead to a second autoimmune burst signal, possibly through cytokine release which may attract monocytes, mast cells, natural killers, eosynophils, neutrophils, and repair stromal cell that may infiltrate the inflamed heart [24, 25, 54, and 120]. Considering that protean clinical manifestations are present in some Chagas disease cases, we postulate that immune-competent cells bearing different genotype alterations can target other tissues in addition to the heart, skeletal muscles and neuronal cells in the body [1], [4]–[6].

In humans, approximately 30% of the individuals infected with *T. cruzi* develop chronic Chagas heart disease (94.5%) and/or megacolon and megaesophagus (5.5%) [3]. The clinical data suggest that the myocarditis is the hallmark of Chagas heart disease [3]–[6]. Additionally, Chagas disease lesions affect the parasympathetic nervous system and skeletal muscles, albeit to a much lesser degree. The clinical information [3]–[6] indicate that in a great majority of cases Chagas (average death 40±5 years old) is an organ specific autoimmune disease. The autoimmune phenomenon that targets the heart is the result of somatic mutations that cannot be random because the chicken TEs hotspot repeats shared with the parasite minicircle kDNA, possibly, anticipate the outcome of the heart disease in vertebrate hosts.

An alternative explanation for the basic autoimmune mechanisms in Chagas disease addresses cross-reactive antigens after exposure of mimicry epitopes shared with the hosts' self proteins [51], [58], [59]. However, antigen and epitope definition requires antibody recognition and, therefore, in the lack of antibodies to self tissue antigens, the antigenic mimicry hypothesis did not hold up rationality to explain the parasite-free autoimmune Chagas-like disease, but the hypothesis that peptides bound to MHC molecules can trigger the autoimmune mechanism needs to be evaluated experimentally, since invading T cells destroy target host tissues [94], [95]. Moreover, various attempts to associate autoantibodies against self's myosin and its B13 epitope and against parasite specific antigens [58], [59] paralleled the experimental counterpart animal models of Hashimoto thyroiditis [55], EAE [56], and CVB3 myocarditis [57], in which the adjuvant effect was necessary to provide the second signal that triggers inflammatory lesions [53]. Such effect is encountered in the induction of experimental autoimmune diseases through administration of microbe antigens emulsified with non-metabolizable paraffin oil and mannide monooleate containing killed *Micobacterium tuberculosis*; the relationship between severe inflammatory disease and arthritogenicity with adjuvanticity has been described [121]. Interestingly, other adjuvants fail to induce autoimmune disease, although they may elicit autoantibody production [53]. For example, myocarditis can be elicited in susceptible mice by administration of cardiotrophic CVB3 if an additional adjuvant is given through pro-inflammatory cytokines IL-1 and TNF-α [25], [121].

In the absence of clinical manifestations associated with autoantibodies [22], [32], [33], our hypothesis sustains that the Chagas heart disease is an antigen-independent autoimmune phenomenon, which associates first signal somatic mutations with overreactivity of cytotoxic T lymphocytes, which infiltrates and rejects target tissues in the parasite-free transkingdom chicken model system. On the one hand, after several decades, science did not provide satisfactory treatment for autoimmune disease with the basis of the conventional mechanisms [24, 54, and 56], which required bolstering with unspecific adjuvant effect leading to recrudescence of inflammatory reactions. On the other hand, somatic mutations, which lead to the disease, suggest that the treatment of fatal Chagas heart disease can be achieved through histocompatible BMC transplantation.

The Prague congenic chickens are among the best genetically defined model systems for immunological studies [91]–[93]. We introduced modifications in the genome of syngeneic CB (B^12^/B^12^) chickens through the inoculation of *T. cruzi* trypomastigotes into fertilized eggs prior to incubation. The chicks hatched from the *T. cruzi-*inoculated eggs contained sequences from *T. cruzi*, and Southern blot revealed high molecular weights (18 and 20 kb) kDNA bands formed with the *Eco*RI digests of somatic cell's DNA of the heart, kidney, liver, skeletal muscle, esophagus, large bowel, and blood mononuclear cells.

The absence of the nDNA bands revealed the refractoriness of the chickens to *T. cruzi* infection [10], [66]. Sequencing showed the parasite mitochondrion kDNA minicircle integrated into several chromosomes. The chimeras were often present in repetitive elements, mainly non-LTR retrotransposon CR1 (82%). Often the kDNA mutations were found in coding regions at various *loci* of macrochromosomes shown in Tables S2 and S4. For example, we analyzed the topological features of kDNA integrations in dystrophin, *NADPME*, and other genes (S2 and S4 Tables) that demonstrated disruption of the ORFs. Interestingly, some physiopathological consequences from the insertional mutagenesis were documented in the progeny of three chickens that presented with muscle weakness, inflammatory cardiomyopathy, and kDNA mutations in the dystrophin gene. The dystrophin cluster spans 2.4 Mb on the human X chromosome. Mutations in the gene can be associated with the absence of the dystrophin cytoskeletal β-spectrin/α-actinin protein family located primarily in skeletal muscles and in the heart. The dystrophin protein complex acts as an anchor, connecting the cytoskeleton to the cell membrane components, and the mutation can be associated with clinically manifested muscular dystrophy syndromes, affecting the contractile movements of skeletal and heart muscles [121]–[124].

In this study, two levels of pathological effects were recorded: i) kDNA mutations and genotype modifications of immune effector lymphocytes that carried out accelerated rejection of target cells; and ii) kDNA mutations that undergo biochemical alterations and lesions such as those described for the dystrophin gene [122]–[124]. Although the kDNA mutations and genotype modifications documented in several *loci* at various chromosomes of chicken flocks were often silent for long periods of time, they have the potential for the development of Chagas heart disease, and therefore, random physiopathological effects on different target organs (Fig. 4C, 4D, and 4E) cannot be ruled out.

The chickens genetically modified by the integration of the kDNA minicircles reached sexual maturity and were subjected to a series of experiments that demonstrated the unique role played by parasite-induced genomic modifications, associated with the pathogenesis of heart disease observed in the kDNA+ chickens only. We often observed that some kDNA+ birds developed clinical manifestations, such as cyanosis, shortness of breath, hydrothorax, and ascites, and exhibited evidence of cardiomegaly when they died. Microscopy revealed the myocardial fibers destroyed by cytotoxic effector lymphocytes. The dynamics of the lesions in the chicken's heart were then studied by intravenous injections of fluorochromes that stained the BMCs and the blast cells in the splenic lymphoid follicles. These results were consistent with the often-evident clonally proliferation of immune cells that reached the blood vessels and attacked the myocardium of the kDNA+ chickens. These effector lymphocytes formed MRUs, and the confluence of several MRUs produced diffuse myocarditis in the hearts of kDNA+ chickens. However, apoptosis of the immune cells in the spleens and hearts of kDNA+ chickens was observed. Altogether, the results suggest that the immune systems of the genetically modified chickens underwent cyclical changes and thus experienced multifaceted relapses and recrudescence of disease activity until heart failure.

The production of the heart lesions in NC chickens was investigated through the passive transfer of fluorochrome-labeled blood mononuclear cells from kDNA+ chickens. After a series of three intravenous injections of immune cells, we euthanized the NC recipient chickens to assess the immune cell infiltrates in the heart. Microscopy revealed small lymphocyte infiltrates in the hearts of the NC chickens receiving kDNA+ cells only. Passive transfer demonstrated that the kDNA+ chickens' immune cells, but not the NC immune cells, had inherent heart-homing ability, even in the absence of clinical manifestations of heart pathology. These results are consistent with previous findings that gross Chagas heart pathology cannot be obtained through the passive transfer of short half-life blood mononuclear cells [62]–[64]. However, the adherence of some blood lymphocytes to heart cells was evident, and this finding suggested that the transplantation of BMC lymphoblast progenitors, which are undergoing continuous proliferation and differentiation into kDNA+ or kDNA- lymphocytes, could induce or prevent heart pathology, respectively.

The grafting technique used to monitor the development of the lesions in the hearts of the kDNA+ chickens revealed that the fluorochrome-labeled green or red immune cell infiltrates rejected the one-day-old syngeneic heart grafts by 11 days, whereas syngeneic grafting was accepted in the NC chickens, with no rejection at 17 days. We then used the grafting technique to monitor the effects of the transfer of the BMCs from kDNA+ or NC animals to recipients that had their BMCs destroyed with anti-folate and cytostatic drugs. The transfer of kDNA+ BMCs to NC birds resulted in lymphocyte infiltrates and rejection of the syngeneic heart grafts. Moreover, the heart graft rejection was inhibited by the transfer of the BMCs from NC to kDNA+ chickens that had their marrow cells destroyed with the drugs. This observation was confirmed by the mock controls, consisting of the transfer of healthy BMCs to NC chickens that had their marrow cells destroyed with the same drugs, and these birds did not reject the syngeneic heart grafts.

After the documentation of the clinical manifestations stemming from the development of heart pathology in the syngeneic chickens that retained the kDNA minicircles in their genomes, we documented the genetic modifications resulting from integration into several loci on the chicken chromosomes. A timely search for the familial genetic susceptibility ascribed to some autoimmune diseases was conducted. The crossbreeding of the kDNA+ chickens generated descendants, and a non-Mendelian type of inheritance of the minicircle sequences and their genetic role in the susceptibility to autoimmune disease was observed.

The crossbreeding of the F0 kDNA+ chickens generated F1, F2, and F3 progeny that exhibited kDNA mutations mapping to several chromosomes. An important feature of the kDNA mutations was frequent integration into TEs in the chicken genome, particularly at the non-LTR transposable CR1 repeats. Additionally, the kDNA mutations were frequently observed in macrochromosomes encoding genes associated with cell metabolism, replication, growth, and important cytoskeletal functions, as described in previous papers [10, 33, and 44]. A linear relationship between the kDNA-mutations and the development of heart pathology was not observed. Complete sequencing of the kDNA-mutated chicken genomes will hopefully shed light on this matter.

Interestingly, arrays of various somatic mutations that were widely spread in the genome of humans, rabbits, and chickens have not been associated with tumor growth [10, 65, 66, and 67]. Clinical-epidemiological studies have shown that chronic *T. cruzi* infection in humans bear a negative relationship with cancer [125], [126], and it has been reported that the protozoan *T. cruzi* may be an effective cancer antigen delivery vector [127]. We postulated that in the *T. cruzi* refractory chicken model system, in the absence of live infection and of tumor growth, the reported negative relationship [125]–[127] should be explained through those TEs repeat-motifs present in kDNA CRs sequences shared microhomology hotspots with hundreds of thousands of TEs spread in the chicken genome [85], whose somatic mutations driving autoimmune disease fall short of cancerigenesis.

The life spans of the chickens with genome modifications were significantly shorter than those of the controls. However, the factors contributing to the aggravation of the lesions and heart failure in the kDNA+ chickens are unknown. In this regard, we used the reporter heart graft technique to evaluate the autoimmune status of the parental and progeny chickens at 10 months of age. We observed that the syngeneic heart grafts were mildly to severely rejected by the kDNA-mutated chickens' lymphocytes, whereas the control chickens retained the grafts in the absence of an attack by the immune system. These results suggest that the chickens' immune tolerance was genetically controlled and that the disrupted immune tolerance observed exclusively in the kDNA+ chickens had severe physiopathological consequences, resulting in autoimmune graft rejection. Thus, the clinical manifestations associated with the extensive heart pathology observed in these chickens appear to be a threshold phenomenon.

We next sought to determine the type of inheritance of the kDNA mutations. Based on our previous work [10], [65]–[67], we anticipated that frequent foreign DNA integrations into the TEs in the chicken genome would occur; we also observed hundreds of other mutations that underwent fixation in the progeny. A total of 200 minicircle sequences from host DNA-kDNA somatic chimeras from our laboratory were deposited in the EMBL database. Herein, an enormous diversity present in the kDNA minicircle variable regions was observed, but the effects of mismatching no longer prevented robust bioinformatic analyses [10], [67]. Additionally, we predicted several dozen gRNAs stemming from the chimeric minicircle variable regions. Sequence analysis revealed that the chimeras fixed in the chicken genome underwent extensive structural modifications due to recombination, reshuffling, and hitchhiking from a primary site to a second chromosomal location at a distance. The inevitable modifications of the host's genome explain the structural differences between a kDNA mutation in the progeny and the parental chimera at that single locus. As a consequence of the topological changes in the kDNA-host DNA chimeras, non-Mendelian inheritance drove molecular evolution through the mutations. These mutations cannot have been neutral because they bore potential pathophysiological consequences. The alternation between homeostatic physiology and pathophysiological manifestations, characterized by disease activity relapse and remission, has been associated to some extent with the potential for retrotransposable elements to remodel the effector cell genome following kDNA-driven mutation, and therefore, the arrays of cytotoxic T lymphocytes phenotypes fit the definition of 'forbidden clones' that carry out self's tissue rejection [17].

The disruption of immune tolerance prior to the appearance of clinical manifestations suggests the possibility of treatment of autoimmune cardiomyopathy in the chicken model system. The transplantation of healthy BMCs into sick kDNA+ chickens that had their marrow cells destroyed with drugs clearly resulted in the absence of rejection of the syngeneic grafts, with a lack of inflammatory cell infiltrates and no evident heart pathology in adult chickens. In contrast, the transplantation of sick BMCs to NC recipients that had their marrow cells destroyed with drugs resulted in severe rejection of the grafts and of the resident hearts as well. Therefore, we searched for neo-antigens stemming from the putative ORF-derived chimeras at the host DNA-kDNA junctions. Two-D gel electrophoresis did not reveal differences in the patterns of protein spots. The inflammatory C-reactive protein was the only over-expressed protein in the kDNA+ chickens. Collectively, the data indicated that the immune effector lymphocytes primarily destroyed the target heart cells. Furthermore, a role for humoral immunity was not detected due to the absence of auto-antibodies in the chickens, which acquired complete immune tolerance early in embryonic development [119].

The refractoriness of the transkingdom chicken model to the *T. cruzi* parasite was highly convenient for this study of the treatment of the heart pathology because it did not require pre-clearing the infection. The inhibition of autoimmune heart disease by the replacement of sick kDNA+ BMCs with healthy BMCs from a compatible donor holds promise for the timely treatment of Chagas heart disease in humans, which is now a global health problem on five continents [1], [2]. However, for this treatment to be effective, new drugs must be developed to ensure complete pre-clearance of the *T. cruzi* infection, which cannot be anticipated in light of the scarce research in this field.

Together, the findings documented in this study confirm and extend the work of previous studies that have demonstrated antigen-independent, genetically driven, autoimmune phenomena in Chagas disease [10, 65, 66, and 67]. The unraveling of the pathogenesis of the autoimmune disease is expected to be a precursor to preventing otherwise unavoidable heart failure. This achievement will transform the current dogma of immunology, which maintains that the autoimmune rejection of self-tissue requires a so-called auto-antigen, super-antigen, and/or cross-reactive alien antigen that mimics the host's constituents and elicits an immune response that destroys its own body [48-46]. This canonical speculation has never been proven to represent the full complement of the pathology, as is clearly documented in human Chagas cases and in the chicken model of autoimmune Chagas heart disease [1], [88]. Thus, with the goal of discovering treatments for this still incurable disease, constructive criticism is encouraged.

Ongoing research aimed at demonstrating the associations between microbes and highly prevalent autoimmune disease [23] anticipates the necessity of pre-clearing the host with drugs to eliminate effectively the specific infectious agent and of bone marrow transplantation for the treatment of the ailment. Additionally, we postulate that organic and inorganic compounds, and physical agents as well, could induce autoimmune diseases resulting from promiscuous linking to macromolecules, forming DNA-adducts, and the resulting genetic modifications might lead to the disruption of immune tolerance and autoimmune disease. Scientific advancements in the field of tissue typing and identification of compatible bone marrow donors indicate possible future success in the treatment of human autoimmune diseases [128]–[131].

### Genes

The genes and loci in the text are as follows:

The *NADPME* locus NW_003763650.1; *CR1* locus NW*_*003763785.1 (HG531593); locus NW_0037636687.1 (HG531594); locus NW_001471668.2 (HG 531596); and locus BX640540.3 (HG531644). *SOX*-6 locus NW_003763785.1 (HG531593, HG531392); dystrophin locus emb|V01390.1; gbL26251.1; pyruvate dehydrogenase kinase locus NW_001471534.1 (HG531471); cytospin A locus NW_001471461.2 (HG531751) (see S2 Table).

## Supporting Information

S1 Fig Two-dimensional electrophoresis profiles of cardiac proteomes from kDNA+ and kDNA- syngenic roosters.
(TIF)

S1 Table PCR primers.
(DOCX)

S2 Table Lateral transfer of kDNA minicircle sequences from *Trypanosoma cruzi* to the genome of *Gallus gallus* somatic cells.
(DOCX)

S3 Table Putative gRNAs within kDNA-host DNA chimera sequences in the *Gallus gallus* genome.
(DOCX)

S4 Table ORF-translated chimera protein sequences from the kDNA+ *Gallus gallus* somatic cell genome.
(DOCX)
