# Supplementary figures and images for: Inhibition of Autoimmune Chagas-Like Heart Disease by Bone Marrow Transplantation
Source: PLoS Negl Trop Dis. 2014 Dec 18;8(12):e3384. doi: 10.1371/journal.pntd.0003384 (PMC4270743; doi:10.1371/journal.pntd.0003384)

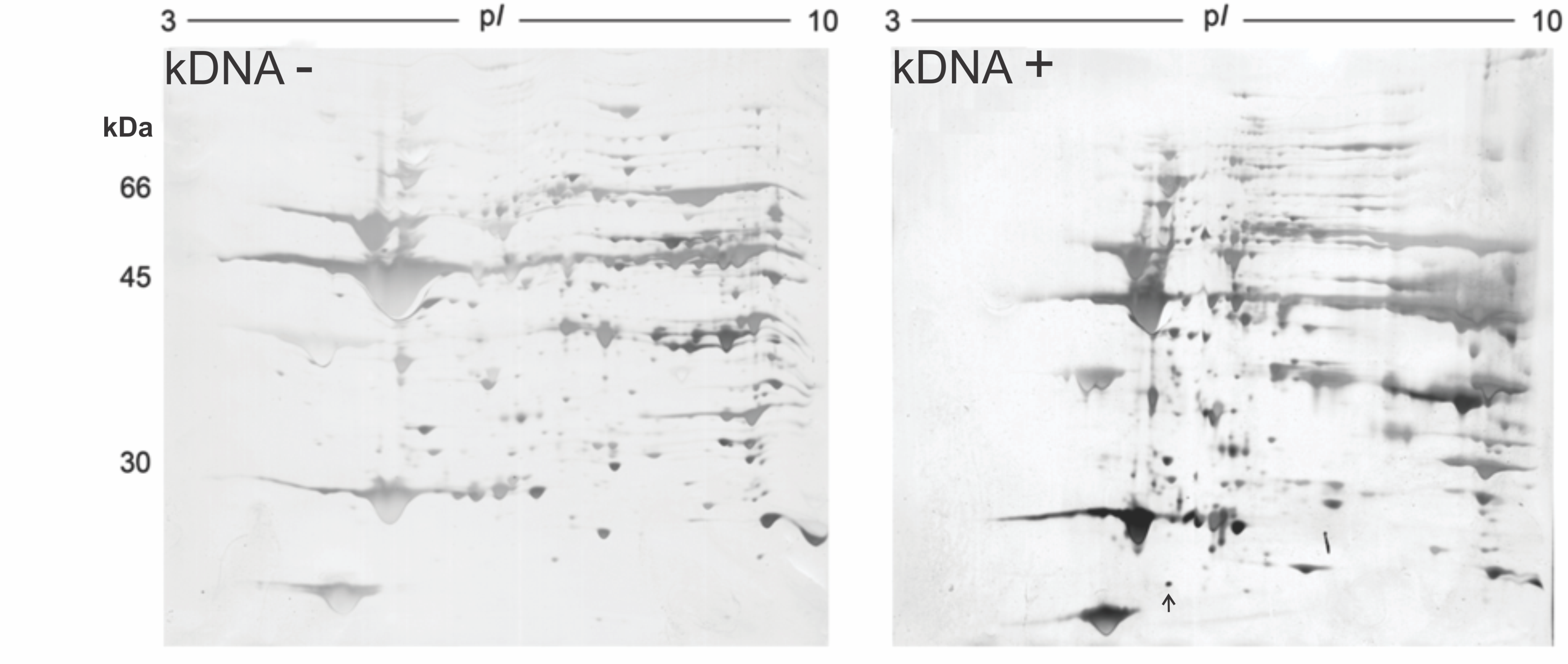

Supplement: S1 Fig — Two-dimensional electrophoresis profiles of cardiac proteomes from kDNA+ and kDNA- syngenic roosters. (TIF) [file pntd.0003384.s001.tif]
