# Supplementary material for: Inhibition of Autoimmune Chagas-Like Heart Disease by Bone Marrow Transplantation
Source: PLoS Negl Trop Dis. 2014 Dec 18;8(12):e3384. doi: 10.1371/journal.pntd.0003384 (PMC4270743; doi:10.1371/journal.pntd.0003384)
Supplement: S1 Table — PCR primers. (DOCX) [file pntd.0003384.s002.docx]

**Table S1.** PCR primers.

| **Primer** | **Target** | **Sequence** | **Tm*** |
| --- | --- | --- | --- |
| TCZ1 | *T. cruzi* nDNA | 5’ GAG CTC TTG CCC CAC ACG GGT GCT 3’ | 67.6 |
| TCZ2 | *T. cruzi* nDNA | 5’ CCT CCA AGC AGC GGA TAG TTC ACG 3’ | 61.4 |
| S34 | *T. cruzi* kDNA | 5’ ACA CCA ACC CCA ATC GAA CC 3’ | 57.9 |
| S67 | *T. cruzi* kDNA | 5’ GGT TTT GGG AGG GG(G/C) (G/C)(T/G)T C 3’ | 60.1 |
| S35 | *T. cruzi* kDNA | 5’ ATA ATG TAC GGG (T/G)GA GAT GC 3’ | 59.4 |
| S36 | *T. cruzi* kDNA | 5’ GGT TCG ATT GGG GTT GGT G 3’ | 57.9 |
| CC1 | *G. gallus* DNA | 5’ AGA GAA CTG CTT GAA ACC TAC AGG 3’ | 56.6 |
| CC2 | *G. gallus* DNA | 5’ GTG AAA GGC CTA TAT CAA GTC AGC 3’ | 55.5 |
| CC3 | *G. gallus* DNA | 5’ CCA AGG AGC TGG TAG AGA GCA 3’ | 58.5 |
| CC4 | *G. gallus* DNA | 5’ CCA CGC TGG GAG AAG AGT T 3’ | 57.1 |
| CC5 | *G. gallus* DNA | 5’ CTC ACT TCC TCC CTT CCC A 3’ | 56.1 |
| CC6 | *G. gallus* DNA | 5’ TGC TCT CTA CCA GCT CCT TGG 3’ | 58.5 |
| CNNM2 | *G. gallus* DNA | 5’ ACCATTCTTGTGACTGCCCTG 3’ | 62.0 |
| DC1 | *G. gallus* DNA | 5’ TTCTAGTCCGCTCCTGTTCAA 3’ | 61.3 |
| DC2 | *G. gallus* DNA | 5' AAA GCA TTC CAG TGG CAG AA 3' | 60.8 |
| NADPME1 | *G. gallus* DNA | 5'CCA GAG GCA CAT GGG TGT AA 3' | 61.9 |
| NADPME2 | *G. gallus* DNA | 5' TTG TCC TGC AGC CAT ATG G 3' | 60.6 |
| XeCRs1 | *G. gallus* DNA | 5’ ATW TCW GTS TTT GCA GAT GAC ACA 3’ | 54.0 |
| XeCRs2 | *G. gallus* DNA | 5’ CTT WGT TGC CCT YCT CTG KAC YCT CTC YA 3’ | 61.0 |
| XeCRs3 | *G. gallus* DNA | 5’ TGT GTC ATC TGC AAA SAC WGA WAT 3’ | 54.0 |
| XeCRs4 | *G. gallus* DNA | 5’TRG AGA GRG TMC AGA GRA GGG CAA CWA TG 3’ | 61.0 |

*Tm = average annealing temperature ºC
