# Supplementary material for: Inhibition of Autoimmune Chagas-Like Heart Disease by Bone Marrow Transplantation
Source: PLoS Negl Trop Dis. 2014 Dec 18;8(12):e3384. doi: 10.1371/journal.pntd.0003384 (PMC4270743; doi:10.1371/journal.pntd.0003384)
Supplement: S2 Table — Lateral transfer of kDNA minicircle sequences from Trypanosoma cruzi to the genome of Gallus gallus somatic cells. (DOCX) [file pntd.0003384.s003.docx]

**Table S2.** Lateral transfer of minicircle sequences of kDNA from *Trypanosoma cruzi* to the genome of *Gallus galllus* somatic cells.

| Ave | EMBL | kDNA | *G. gallus* DNA | Intermediate recombination site | kDNA  *E-value* | kDNA  Identity | *G. gallus*  *E-value* | *G. gallus*  Identity | *G. gallus* Chromosome | *Locus* | *G. gallus* repeats |
| --- | --- | --- | --- | --- | --- | --- | --- | --- | --- | --- | --- |
| **1** | HG531391 | 1-285 | 284-460 | CC | 1e^-136^ | 99% | 1e^-29^ | 78% | Not determined | NW_001479132.1  (Not determined) | --- |
| **1** | HG531392 | 427-682 | 1-447 | TATAATGTACGGGGGAGATGC | 1e^-119^ | 98% | 0.0 | 98% | 5 | NW_003763785.1  (Transcription factor SOX-6) | --- |
| **1** | HG531393 | 321-611 | 1-328 | GACCGCCC | 6e^-136^ | 98% | 2e^-156^ | 98% | 2 | NW_003763668.1  (Not determined) | --- |
| **2** | HG531394 | 1-310 | 293-508 | ACCAACCCCAATGGAACC | 1e^-04^ | 79% | 5e^-99^ | 98% | 1 | NW_001471534.2  (Dystrophin) | --- |
| **2** | HG531395 | 1-266 | 249-464 | ACCAACCCCAATGGAACC | 2e^-128^ | 99% | 3e^-95^ | 96% | 1 | NW_001471534.2  (Dystrophin) | --- |
| **2** | HG531396 | 1-231 | 214-429 | ACCAACCCCAATGGAACC | 2e^-108^ | 98% | 2e^-97^ | 97% | 1 | NW_001471534.2  (Dystrophin) | --- |
| **2** | HG531397 | 102-182 | 1-101 | - | 6e^-28^ | 97% | 5e^-42^ | 98% | 14 | NW_003763931.1  (Not determined) | --- |
| **2** | HG531398 | 166-453 | 1-98 / 99-174 | GACCGCCCC | 5e^-22^ | 87% | 1e^-35^ /  6e^-27^ | 95% /  97% | 14 / 5 | NW_003763931.1  (Not determined) /  NW_003763785.1  (Not determined) | --- |
| **3** | HG531399 | 1-263 /  459-600 /  740-835 | 246-461 /  597-742 | ACCAACCCCAATGGAACC /  GAA /  GAGA /  TAC | 1e^-126^ /  5e^-59^ /  4e^-20^ | 99% /  97% /  85% | 8e^-99^ /  3e^-66^ | 98% /  99% | 1 /  Not determined | NW_001471534.2  (Dystrophin) /  NW_001471746.1  (Not determined) | --- |
| **3** | HG531400 | 1-149 | 140-305 /  288-503 | GGGAGATGCA | 4e^-48^ | 89% | 2e^-72^ /  2e^-96^ | 98% /  97% | Not determined /  1 | NW_001471746.1  (Not determined) /  NW_001471534.2  (Dystrophin) | --- |
| **3** | HG531401 | 1-137 | 128-599 | CAATCGAACC | 4e^-06^ | 76% | 0.0 | 96% | 20 | NW_003764128.1  (Not determined) | --- |
| **3** | HG531402 | 173-441 | 1-185 | GACCCCCCCTCCC | 2e^-06^ | 81% | 4e^-87^ | 99% | 3 | NW_001471668.2  (probable palmitoyltransferase ZDHHC14 isoform 1 and 2) | CR1 (Non LTR) |
| **3** | HG531403 | 236-317 | 1-247 | GAACCCCCCTCC | 6e^-10^ | 97% | 7e^-113^ | 96% | 2 | NW_001471639.1  (Not determined) | --- |
| **3** | HG531404 | 102-244 | 1-125 | GAAGCCCCCTCCCAAAACCATAAT | 4e^-23^ | 92% | 2e^-42^ | 91% | 8 | NW_001471740.1  (Not determined) | --- |
| **3** | HG531405 | 523-647 | 1-538 | AAATAATGTACGGGGG | 4e^-22^ | 94% | 0.0 | 95% | 8 | NW_001471740.1  (Not determined) | --- |
| **3** | HG531406 | 148-290 | 1-155 | GACCGCCC | 1e^-56^ | 98% | 2e^-68^ | 97% | 1 | NW_001471551.1  (Similar to protocadherin-9 isoform 1and [2](http://www.ncbi.nlm.nih.gov/entrez/viewer.fcgi?val=118084795&db=Nucleotide&from=1737993&to=2446821&view=gbwithparts&RID=K8FZ7MZB01R)) | CR1 (Non LTR) |
| **4** | HG531407 | 487-741 | 1-502 | ATAATGTACGGGTGGG | 5e^-119^ | 98% | 0.0 | 99% | 6 | NW_003763812.1  (Metal transporter CNNM2 isoform 2) | --- |
| **4** | HG531408 | 1-148 | 142-251 | GAGATGC | 5e^-53^ | 94% | 6e^-43^ | 97% | 5 | NW_001471698.1  (Dickkopf homolog 3) | --- |
| **4** | HG531409 | 1-94 | 77-869 | ACCAACCCCAATGGAACC | 1e^-32^ | 95% | 0.0 | 99% | 3 | NW_001471673.1  (Not determined) | Hitchcock (LTR) |
| **4** | HG531410 | 1-94 | 80-534 | AACCCCAATGGAACC | 5e^-32^ | 93% | 0.0 | 93% | 17 | NW_001471503.1  (Not determined) | Hitchcock (LTR) |
| **4** | HG531411 | 1-100 | 79-217 | CAACCCCAATGGAACCAGACCT | 1e^-32^ | 94% | 7e^-61^ | 98% | 17 | NW_001471503.1  (Protein-O-mannosyltransferase 1)  (Proteína O-manosiltransferase 1 - POMT1) | --- |
| **4** | HG531412 | 301-395 | 1-319 | TGAACGCCCCTCCCAAAAC | 2e^-32^ | 95% | 1e^-143^ | 96% | 3 | NW_001471673.1  (Not determined) | Hitchcock (LTR) |
| **4** | HG531413 | 194-329 | 1-202 | ATAATGTAC | 2e^-56^ | 97% | 2e^-96^ | 99% | 6 | NW_003763812.1 (Metal transporter CNNM2 isoform 2) | --- |
| **4** | HG531414 | 150-244 | 1-165 | TGAACGCCCCTCCCAA | 2e^-32^ | 95% | 3e^-53^ | 93% | 11 | NW_001471432.1  ([WD repeat domain 59](http://www.ncbi.nlm.nih.gov/nucleotide/118096260?report=gbwithparts&from=1129753&to=1177348&RID=ME9NW1ED016)) | CR1 (Non LTR) |
| **4** | HG531415 | 240-334 | 1-253 | TGAACGCCCCTCCC | 7e^-32^ | 95% | 1e^-109^ | 95% | 3 | NW_001471673.1  (Not determined) | --- |
| **4** | HG531416 | 341-738 | 1-349 | GAACCCCCC | 1e^-110^ | 94% | 1e^-166^ | 98% | 13 | NW_001471449.1  (Not determined) | --- |
| **4** | HG531417 | 125-647 | 1-127 | GAC | 4e^-151^ | 98% | 1e^-50^ | 96% | 8 | NW_001471740.2  (Not determined) | --- |
| **4** | HG531418 | 139-484 | 1-163 | ATTCTCACTTCCTCCCTTCCCAAAA | 3e^-111^ | 95% | 2e^-53^ | 95% | 1 | NW_001471513.1  (Not determined) | --- |
| **4** | HG531419 | 302-589 | 1-319 | GACCCCCCCTCCCAAAAC | 3e^-140^ | 99% | 2e^-142^ | 95% | 3 | NW_001471673.2  (Not determined) | Hitchcock (LTR) |
| **4** | HG531420 | 301-540 | 1-319 | GAACGCCCCTCCCAAAACC | 8e^-117^ | 99% | 6e^-142^ | 96% | 3 | NW_001471673.2  (Not determined) | Hitchcock (LTR) |
| **4** | HG531421 | 265-358 | 1-284 | GAACGCCCCTCCCAAAACCA | 2e^-33^ | 95% | 5e^-122^ | 95% | 2 | NW_003763673.1  (Not determined) | --- |
| **5** | HG531422 | 102-316 | 1-110 | GAACCCCCC | 3e^-46^ | 90% | 1e^-25^ | 86% | 9 | XM_419045.2  (Chromosome 9 open reading frame 4) | CR1 (Non LTR) |
| **5** | HG531423 | 86-361 | 1-103 | GAACCCCCCTCCCAAAAC | 1e^-64^ | 89% | 7e^-32^ | 92% | Not determined | NW_001477517.1  (Not determined) | CR1 (Non LTR) |
| **5** | HG531424 | 89-414 | 1-98 | TGAACGCCCC | 3e^-99^ | 92% | 9e^-31^ | 92% | 8 | NW_001471740.1 ([Similar to protein tyrosine phosphatase, receptor type, F](http://www.ncbi.nlm.nih.gov/nucleotide/118094775?report=gbwithparts&from=8913288&to=9131655&RID=N2Z318MM014) – PTPRF) | --- |
| **5** | HG531425 | 55-261 | 1-70 /  253-379 | GAACGCCCCTCCCAA A /  AACCATTAT | 2e^-95^ | 99% | 1e^-15^ /  1e^-16^ | 89% /  76% | 1 / Not determined | NW_001471556.1  (Not determined) /  NW_001479132.1  (Not determined) | --- |
| **5** | HG531426 | 192-457 | 1-199 | TTTGAACG | 1e^-130^ | 99% | 4e^-93^ | 99% | 1 | NW_001471512.1  (Tetraspanin-12) | --- |
| **5** | HG531427 | 152-485 | 1-166 | ATAA TGTACGGGTGA | 4e^-111^ | 97% | 8e^-71^ | 96% | 13 | NW_001471446.1  (Not determined) | --- |
| **5** | HG531428 | 197-468 | 1-208 | CCCTTCCCAAAA | 5e^-90^ | 98% | 3e^-76^ | 94% | Not determined | NW_001475305.1 (Similar to FLJ20433 protein) | --- |
| **5** | HG531429 | 265-590 | 1-281 | GACGGCCCCTCCCAAAA | 2e^-115^ | 92% | 3e^-133^ | 98% | 2 | NW_001471633.1  (Not determined) | --- |
| **5** | HG531430 | 91-233 | 1-108 | GAACCCCCCTCCCAAAAC | 1e^-55^ | 99% | 4e^-32^ | 95% | 2 | NW_001471633.1  (Similar to retina-derived POU-domain factor-1) | CR1 (Non LTR) |
| **5** | HG531431 | 407-549 | 1-408 | GA | 7e^-58^ | 97% | 0.0 | 99% | 1 | NW_001471519.1  ([hypothetical protein](http://www.ncbi.nlm.nih.gov/nucleotide/118081868?report=gbwithparts&from=266754&to=383549&RID=N3UFY3U1014)) | CR1 (Non LTR) |
| **6** | HG531432 | 71-391 | 1-93 | ATAATGTACGGGTGA GATGCATG | 3e^-21^ | 88% | 3e^-31^ | 93% | 15 | NW_001471461.1  (Not determined) | --- |
| **6** | HG531433 | 223-513 | 1-234 | GACGGCCCCTCC | 2e^-06^ | 71% | 8e^-100^ | 93% | Z | AC193222.4  (Not determined) | --- |
| **7** | HG531434 | 1-68 | 51-393 | TCGAACCATCTATCCCAA | 3e^-09^ | 91% | 7e^-165^ | 97% | 16 | NW_001471464.1/ AB268588.1  (MHC region - tenascin X B) | --- |
| **7** | HG531435 | 61-133 | 1-76 | GATAATGTACGGGTGA | 2e^-17^ | 90% | 3e^-24^ | 93% | 11 | NW_001471434.1  (matrix Metalloproteinase 2 - MMP2) | --- |
| **7** | HG531436 | 194-353 | 1-202 | ATAATGTAC | 1e^-20^ | 90% | 2e^-95^ | 98% | 6 | NW_003763812.1  (Metal transporter CNNM2 isoform 2) | --- |
| **10** | HG531437 | 1-308 | 291-506 | ACCAACCCCAATCGAACC | 3e^-95^ | 88% | 4e^-100^ | 98% | 1 | NW_001471534.2  (Dystrophin) | --- |
| **15** | HG531438 | 1-283 | 263-506 | TACACCAACCCCAATCGA ACC | 1e^-134^ | 98% | 5e^-111^ | 96% | 1 | NW_001471554.1  ([Similar to spinal cord-derived growth factor-B](http://www.ncbi.nlm.nih.gov/entrez/viewer.fcgi?val=118085257&db=Nucleotide&from=20229293&to=20366942&view=gbwithparts&RID=P44RMZWE014) - SCDGFB) | --- |
| **15** | HG531439 | 1-286 | 270-465 | CCAACCCCAATCGAACC | 2e^-134^ | 98% | 9e^-89^ | 97% | 9 | NW_001471744.1  (Not determined) | CR1 (Non LTR) |
| **15** | HG531440 | 1-286 | 276-400 | CCAATCGAACC | 6e^-05^ | 84% | 1e^-54^ | 98% | 5 | NW_003763785.1 ([Tetraspanin-18](http://www.ncbi.nlm.nih.gov/entrez/viewer.fcgi?val=118091697&db=Nucleotide&from=3781763&to=3799239&view=gbwithparts&RID=P45P59DM012)) | --- |
| **18** | HG531441 | 1-81 | 61-169 | TACACCAACCCCAATCGAACC | 0.002 | 85% | 6e^-35^ | 90% | 1 | NW_001471554.1  (Not determined) | --- |
| **18** | HG531442 | 1-279 | 262-457 | CCAACCCCAATCGAACCC | 4e^-14^ | 68% | 2e^-91^ | 98% | 9 | NW_001471744.1  (Not determined) | CR1 (Non LTR) |
| **18** | HG531443 | 1-284 | 262-457 | CCAACCCCAATCGAACCCACCAT | 1e^-133^ | 97% | 2e^-90^ | 97% | 9 | NW_001471744.1  (Not determined) | CR1 (Non LTR) |
| **18** | HG531444 | 1-276 | 261-449 | CCAACCCCAATCGAAC | 3e^-21^ | 73% | 1e^-86^ | 97% | 9 | NW_001471744.1  (Not determined) | CR1 (Non LTR) |
| **18** | HG531445 | 1-291 | 275-496 | CCAACCCCAATCGAACC | 2e^-144^ | 99% | 4e^-100^ | 96% | 3 | NW_001471679.1  (Not determined) | CR1 (Non LTR) / ERV3 |
| **18** | HG531446 | 184-468 | 1-197 | GGACGCCCCCTCCC | 8e^-08^ | 79% | 3e^-88^ | 96% | 2 | NW_001471633.1  ([Similar to parathyroid hormone-responsive B1](http://www.ncbi.nlm.nih.gov/entrez/viewer.fcgi?val=118086258&db=Nucleotide&from=47460395&to=47746860&view=gbwithparts&RID=RE3DV2BD014) - PTHB1) | CR1 (Non LTR) |
| **19** | HG531447 | 1-94 | 86-536 | AATCGAACC | 0.002 | 84% | 0.0 | 99% | 10 | NW_001471429.1  (Not determined) | --- |
| **19** | HG531448 | 1-281 | 263-315 | CACCAACCCCAATCGAACC | 2e^-04^ | 88% | 3e^-11^ | 90% | 1 | NW_001471548.1  ([Hypothetical protein](http://www.ncbi.nlm.nih.gov/entrez/viewer.fcgi?val=118084718&db=Nucleotide&from=2603213&to=2617902&view=gbwithparts&RID=RE4PN415015)) | --- |
| **20** | HG531449 | 51-315 | 1-59 | TGAACGCCC | 2e^-105^ | 94% | 5e^-20^ | 99% | 5 | NW_001471710.1  (Not determined) | --- |
| **20** | HG531450 | 64-327 | 1-80 | GAACGCCCCTCCCAAAA | 2e^-106^ | 94% | 1e^-27^ | 97% | 21 | NW_001471571.1  ([Hypothetical protein](http://www.ncbi.nlm.nih.gov/nucleotide/118101178?report=gbwithparts&from=4952289&to=4954669&RID=PP4R4KWN014)) | --- |
| **20** | HG531451 | 49-338 | 1-63 | GAAGCCCCCTCCCAA | 2e^-99^ | 92% | 2e^-18^ | 94% | 22 | NW_001471585.1  (Not determined) | --- |
| **21** | HG531452 | 1-153 | 136-351 | ACCAACCCCAATCGAACC | 4e^-04^ | 84% | 6e^-102^ | 99% | 1 | NW_001471534.2  (Dystrophin) | --- |
| **21** | HG531453 | 306-391 | 1-324 | GACCGCCCCTCCCAAAACC | 5e^-10^ | 81% | 2e^-145^ | 96% | 3 | NW_001471668.2  (Not determined) | --- |
| **22** | HG531454 | 1-152 | 140-461 | GGGAGATGCGTGA | 2e^-53^ | 94% | 2e^-153^ | 98% | 2 | NW_001471654.1  (Similar to RIM2-5B) | --- |
| **22** | HG531455 | 1-149 | 127-262 | ATATAATGTACGGGTGAGATGCA | 1e^-54^ | 95% | 3e^-55^ | 96% | 1 | NW_001471554.1  ([Similar to spinal cord-derived growth factor-B](http://www.ncbi.nlm.nih.gov/nucleotide/118085257?report=gbwithparts&from=20229293&to=20366942&RID=N7V8KMRB01S)- SCDGFB) | --- |
| **22** | HG531456 | 268-489 | 1-288 | AATTTTGAAGGCCCCTCCCAA | 8e^-66^ | 83% | 2e^-141^ | 99% | 2 | NW_001471637.1  (Similar to CDK5 regulatory subunit associated protein 1-like 1) | CR1 (Non LTR) |
| **22** | HG531457 | 185-381 | 1-195 | ATAATGTACGG | 7e^-54^ | 82% | 6e^-90^ | 98% | 14 | NW_001471454.1  ([Similar to mitotic checkpoint protein](http://www.ncbi.nlm.nih.gov/nucleotide/118098117?report=gbwithparts&from=2770369&to=3088511&RID=NE9K6SG7014)) | --- |
| **22** | HG531458 | 86-362 | 1-103 | GAACCCCCCTCCCAAAAC | 6e^-93^ | 86% | 7e^-32^ | 92% | Not determined | NW_001476599.1  (Not determined) | CR1 (Non LTR) |
| **22** | HG531459 | 269-485 | 1-272 | TGAA | 5e^-62^ | 83% | 2e^-121^ | 95% | 10 | NW_001471426.1  (Protogenin) | --- |
| **22** | HG531460 | 125-560 | 1-137 | GACCCCCCCTCCC | 6e^-116^ | 92% | 2e^-52^ | 94% | 8 | NW_001471740.2  (Not determined) | --- |
| **22** | HG531461 | 71-165 | 1-79 | TGAAGCCCC | 1e^-29^ | 93% | 6e^-28^ | 95% | 1 | NW_001471534.1  (Not determined) | --- |
| **22** | HG531462 | 134-393 | 1-145 | CCCTTCCCAAAA | 8e^-100^ | 93% | 3e^-50^ | 96% | Not determined | NW_001475305.1 (Similar to FLJ20433 protein) | --- |
| **22** | HG531463 | 67-203 | 1-88 | AATAATGTACGGGGGAGATGCA | 4e^-49^ | 93% | 2e^-29^ | 94% | 6 | NW_001471715.1  (Not determined) | --- |
| **22** | HG531464 | 68-264 | 1-88 | ATAATGTACGGGGGAGATGCA | 1e^-56^ | 83% | 2e^-29^ | 94% | 6 | NW_001471715.1  (Not determined) | --- |
| **22** | HG531465 | 51-320 | 1-73 | AACCTGAACCCCCCTCCCAAAAC | 3e^-103^ | 93% | 1e^-21^ | 94% | 4 | NW_001471681.1  (Not determined) | CR1 (Non LTR) |
| **23** | HG531466 | 1-260 | 243-458 | ACCAACCCCAATCGAACC | - | - | 3e^-100^ | 98% | 1 | NW_001471534.2  (Dystrophin) | --- |
| **23** | HG531467 | 1-263 | 246-459 | ACCAACCCCAATCGAACC | 5e^-129^ | 99% | 9e^-101^ | 99% | 1 | NW_001471534.2  (Dystrophin) | --- |
| **23** | HG531468 | 418-480 | 1-437 | ATAATGTACGGGGGAGATGC | 6e^-12^ | 100% | 0.0 | 96% | 5 | NW_001471698.1  (Hypothetical protein) | --- |
| **23** | HG531469 | 90-397 | 1-103 | GAAGCCCCCTCCCA | 1e^-95^ | 88% | 4e^-41^ | 97% | 1 | NW_001471513.1  ([Similar to SH3-domain binding protein 1](http://www.ncbi.nlm.nih.gov/entrez/viewer.fcgi?val=118083185&db=Nucleotide&from=7753171&to=7761315&view=gbwithparts&RID=K2J195MS016)) | --- |
| **23** | HG531470 | 109-396 | 1-120 | CCCTTCCCAAAA | 2e^-133^ | 97% | 7e^-39^ | 88% | 1 | NW_001471526.1  ([limbic system-associated membrane protein](http://www.ncbi.nlm.nih.gov/entrez/viewer.fcgi?val=118083519&db=Nucleotide&from=4640571&to=4925899&view=gbwithparts&RID=K2MX0PFJ012)) | --- |
| **23** | HG531471 | 143-433 | 1-143 | A | 2e^-131^ | 96% | 5e^-53^ | 92% | 1 | NW_001471534.1  ([pyruvate dehydrogenase kinase, isoenzyme 3](http://www.ncbi.nlm.nih.gov/nucleotide/118084132?report=gbwithparts&from=17031429&to=17080459&RID=R8SGPG91014)) | CR1 (Non LTR) |
| **24** | HG531472 | 1-281 | 264-464 | ACCAACCCCAATCGAACC | - | - | 1e^-93^ | 99% | 1 | NW_001471534.2  (Dystrophin) | --- |
| **24** | HG531473 | 1-129 | 113-327 | ACCAACCCAATCGAACC | 1e^-45^ | 97% | 8e^-100^ | 98% | 1 | NW_001471534.2  (Dystrophin) | --- |
| **24** | HG531474 | 251-393 | 1-262 | TGACGGCCCCTC | 1e^-54^ | 96% | 6e^-115^ | 96% | 3 | NW_001471669.1  ([Similar to phosphodiesterase 7B](http://www.ncbi.nlm.nih.gov/nucleotide/118088524?report=gbwithparts&from=9302574&to=9467307&RID=N3XZNCYB016)) | CR1 (Non LTR) |
| **24** | HG531475 | 86-411 | 1-103 | GAAGCCCCCTCCCAAAAC | 6e^-133^ | 98% | 8e^-32^ | 92% | Not determined | NW_001476599.1  (Not determined) | CR1 (Non LTR) |
| **24** | HG531476 | 335-497 | 1-339 | GTGAC | 2e^-71^ | 98% | 8e^-172^ | 99% | 7 | NW_001471733.1  (Not determined) | --- |
| **24** | HG531477 | 181-384 | 1-193 | GACGGCCCCTCCC | 2e^-83^ | 97% | 7e^-83^ | 96% | 1 | NW_001471550.1  (Not determined) | CR1 (Non LTR) |
| **24** | HG531478 | 264-468 | 1-284 | TGAAGCCCCCTCCCAAAACCA | 2e^-84^ | 97% | 2e^-115^ | 97% | 2 | NW_001471638.1  ([Similar to GDP-mannose 4, 6-dehydratase](http://www.ncbi.nlm.nih.gov/nucleotide/118086508?report=gbwithparts&from=3784522&to=4183624&RID=N41WUP6001N)) | --- |
| **24** | HG531479 | 301-429 | 1-319 | TGAACCCCCCTCCCAAAAC | 4e^-55^ | 99% | 1e^-143^ | 96% | 3 | NW_001471673.1  (Not determined) | Hitchcock (LTR) |
| **24** | HG531480 | 122-325 | 1-134 | GACCGCCCCTCCC | 8e^-86^ | 97% | 2e^-49^ | 93% | 8 | NW_001471740.2  (Not determined) | --- |
| **24** | HG531481 | 264-468 | 1-281 | TGACCGCCCCTCCCAAAA | 5e^-86^ | 97% | 3e^-133^ | 98% | 2 | NW_001471633.1  (Not determined) | --- |
| **24** | HG531482 | 152-350 | 1-163 | CCCTTCCCAAAA | 1e^-82^ | 97% | 3e^-55^ | 96% | 1 | NW_001471513.1  (Not determined) | --- |
| **24** | HG531483 | 1-277 | 265-365 | GGAGGGGGCGTCA | 2e^-120^ | 96% | 7e^-38^ | 97% | 18 | NW_001471505.1  (Hypothetical protein) | --- |
| **24** | HG531484 | 1-311 | 300-400 | GGAGGGGGGTTC | 3e^-92^ | 81% | 4e^-31^ | 90% | 18 | NW_001471505.1  (Hypothetical protein) | --- |
| **25** | HG531485 | 424-559 | 1-443 | ATAATGTACGGGGGAGATGC | 1e^-49^ | 93% | 0.0 | 98% | 5 | NW_003763785.1  (transcription factor SOX-6) | --- |
| **25** | HG531486 | 236-376 | 1-252 | ATAATGTACGGGTGAGA | 4e^-61^ | 89% | 3e^-119^ | 98% | 3 | NW_001471673.2  (disintegrin and Metalloproteinase domain-containing protein 17 precursor) | --- |
| **25** | HG531487 | 834-920 | 1-851 | ATGGTATGAACCTCCCTC | 2e^-127^ | 93% | 0.0 | 96% | 1 | NW_003763584.1  (Not determined) | CR1 (Non LTR) |
| **25** | HG531488 | 229-418 | 1-244 | GAACGCCCCTCCCAAA | 4e^-80^ | 96% | 2e^-108^ | 96% | 15 | NW_003763985.1  (Not determined) | --- |
| **25** | HG531489 | 81-446 | 1-101 | ATAATGTACGGGTGAGATGCA | 2e^-171^ | 97% | 4e^-36^ | 94% | 2 | NW_003763673.1  (Not determined) | --- |
| **26** | HG531490 | 231-524 | 1-236 | GAAGCC | 2e^-28^ | 73% | 4e^-113^ | 99% | 7 | NW_003763822.1  (zinc finger protein 804A) | --- |
| **26** | HG531491 | 1-203 | 197-525 | GAGATGC | 1e^-93^ | 99% | 6e^-161^ | 98% | 15 | NW_003763985.1  (WSC domain-containing protein 2) | CR1 (Non LTR) |
| **26** | HG531492 | 1-455 | 433-1065 | CATATAATGTACGGGTGAGATGC | 0.0 | 95% | 0.0 | 96% | 1 | NW_003763645.1  (Not determined) | --- |
| **26** | HG531493 | 1-296 | 285-705 | CCCAATCGAACC | 9e^-141^ | 99% | 0.0 | 97% | 7 | NW_003763823.1  (Collagen alpha-1(VI) chain precursor) | --- |
| **26** | HG531494 | 161-555 | 1-160 | - | 2e^-118^ | 84% | 6e^-63^ | 94% | 1 | NW_001471534.2  (Pyruvate dehydrogenase kinase, isozyme 3) | CR1 (Non LTR) |
| **26** | HG531495 | 142-640 | 1-148 | GAACGCC | 1e^-131^ | 91% | 3e^-53^ | 92% | 4 | NW_001471685.2  (Amyloid beta A4 precursor protein-binding family B member 2) | CR1 (Non LTR) |
| **26** | HG531496 | 147-418 | 1-165 | GACCCCCCCTCCCAAAACC | 1e^-92^ | 93% | 3e^-62^ | 94% | 2 | NW_003763668.1  (Eukaryotic translation initiation factor 1b) | CR1 (Non LTR) |
| **27** | HG531497 | 1-302 | 282-525 | TACACCAACCCCAATCGAACC | 2e^-06^ | 80% | 2e^-109^ | 96% | 1 | NW_001471554.1  ([Similar to spinal cord-derived growth factor-B](http://www.ncbi.nlm.nih.gov/entrez/viewer.fcgi?val=118085257&db=Nucleotide&from=20229293&to=20366942&view=gbwithparts&RID=R9GYHMXG015)- SCDGF-B) | --- |
| **27** | HG531498 | 1-301 | 290-365 | CCCAATCGAACC | 4e^-06^ | 71% | 1e^-23^ | 93% | 10 | NW_001471428.1  ([ST8 alpha-N-acetyl-neuraminide alpha-2,8 -sialyltransferase 2](http://www.ncbi.nlm.nih.gov/entrez/viewer.fcgi?val=118095917&db=Nucleotide&from=3895725&to=3920359&view=gbwithparts&RID=R9HGKSK501R)) | --- |
| **27** | HG531499 | 1-308 | 288-531 | TACACCAACCCCAATCGAACC | 7e^-134^ | 97% | 2e^-110^ | 96% | 1 | NW_001471554.1  ([Similar to spinal cord-derived growth factor-B](http://www.ncbi.nlm.nih.gov/entrez/viewer.fcgi?val=118085257&db=Nucleotide&from=20229293&to=20366942&view=gbwithparts&RID=R9JY3YP701R)- SCDGF-B) | --- |
| **27** | HG531500 | 1-291 | 274-324 | ACCAACCCCAATCGAACC | 5e^-05^ | 75% | 4e^-09^ | 88% | Z | NW_001488823.1  (3-oxoacid CoA transferase 1) | --- |
| **27** | HG531501 | 1-295 | 277-328 | ACCAACCCCAATCGAACCC | 3e^-08^ | 68% | 1e^-08^ | 87% | Z | NW_001488823.1  (3-oxoacid CoA transferase 1) | --- |
| **27** | HG531502 | 1-263 | 252-505 | CCCAATCGAACC | 0.001 | 88% | 5e^-86^ | 91% | 2 | NW_001471651.1  (Similar to aminopeptidase) | --- |
| **27** | HG531503 | 1-264 | 246-297 | ACCAACCCCAATCGAACCC | 1e^-04^ | 68% | 1e^-09^ | 88% | Z | NW_001488823.1  (3-oxoacid CoA transferase 1) | --- |
| **27** | HG531504 | 1-263 | 244-279 | ACACCAACCCCAATCGAACC | 0.002 | 91% | 7e^-05^ | 91% | 3 | NW_001471673.1  (Suppressor of Ty 3 homolog) | --- |
| **27** | HG531505 | 1-287 | 271-466 | CCAACCCCAATCGAACC | 4e^-124^ | 95% | 2e^-90^ | 97% | 9 | NW_001471744.1  (Not determined) | CR1 (Non LTR) |
| **27** | HG531506 | 1-308 | 298-423 | CCAATCGAACC | 6e^-151^ | 99% | 2e^-52^ | 98% | 5 | NW_003763785.1 (Tetraspanin-18) | --- |
| **27** | HG531507 | 1-286 | 276-400 | CCAATCGAACC | 5e^-06^ | 86% | 1e^-55^ | 99% | 5 | NW_003763785.1 (Tetraspanin-18) | --- |
| **28** | HG531508 | 270-546 | 1-288 | GAAGCCCCCTCCCAAAACC | 1e^-125^ | 96% | 6e^-136^ | 98% | 1 | NW_003763584.1  ([gamma-aminobutyric acid receptor subunit gamma-3](http://www.ncbi.nlm.nih.gov/nucleotide/358485322?report=gbwithparts&from=7387222&to=7687401&RID=1BRNK2HG013)) | --- |
| **28** | HG531509 | 707-985 | 1-713 | GAACGCC | 3e^-136^ | 99% | 0.0 | 97% | 6 | NW_003763812.1  (Not determined) | --- |
| **36** | HG531510 | 1-286 | 269-464 | CCAACCCCAATCGAACCC | 3e^-07^ | 77% | 1e^-87^ | 96% | 9 | NW_001471744.1  (Not determined) | CR1 (Non LTR) |
| **36** | HG531511 | 71-403 | 1-90 | ATAATATACGGGTGAGATGC | 5e^-19^ | 69% | 1e^-28^ | 91% | 15 | NW_001471461.1  (Not determined) | --- |
| **37** | HG531512 | 1-281 | 271-492 | CCAATCGAACC | 4e^-52^ | 96% | 4e^-86^ | 91% | 1 | NW_001471549.1  (Not determined) | Hitchcock (LTR) |
| **37** | HG531513 | 1-285 | 277-564 | AATCGAACC | 2e^-62^ | 99% | 2e^-140^ | 98% | 15 | NW_001471461.1  (Not determined) | --- |
| **42** | HG531514 | 1-270 | 247-462 | ACCAACCCCAATCGAACCAAGACC | - | - | 8e^-102^ | 99% | 1 | NW_001471534.2  (Dystrophin) | --- |
| **43** | HG531515 | 1-93 | 70-285 | ACCAACCCCAATCGAACCAAGACC | - | - | 5e^-102^ | 99% | 1 | NW_001471534.2  (Dystrophin) | --- |
| **45** | HG531516 | 1-336 | 319-534 | ACCAACCCCAATCGAACC | 1e^-49^ | 76% | 9e^-102^ | 99% | 1 | NW_001471534.2  (Dystrophin) | --- |
| **45** | HG531517 | 185-408 | 1-197 | GACGCCCCCTCCC | 6e^-108^ | 99% | 2e^-89^ | 97% | 2 | NW_001471633.1  ([Similar to parathyroid hormone-responsive B1](http://www.ncbi.nlm.nih.gov/entrez/viewer.fcgi?val=118086258&db=Nucleotide&from=47460395&to=47746860&view=gbwithparts&RID=N9PSSYDY016)) | CR1 (Non LTR) |
| **45** | HG531518 | 185-447 | 1-197 | GAACCCCCCTCCC | 3e^-125^ | 98% | 6e^-84^ | 95% | 2 | NW_003763668.1  ([parathyroid hormone-responsive B1](http://www.ncbi.nlm.nih.gov/entrez/viewer.fcgi?val=118086258&db=Nucleotide&from=47460395&to=47746860&view=gbwithparts&RID=N9RCNY6P014)) | CR1 (Non LTR) |
| **47** | HG531519 | 1-293 | 280-380 | ACCCCAATCGAACC | 2e^-04^ | 73% | 5e^-40^ | 97% | 3 | NW_001471679.1  (Similar to Eml4 protein) | --- |
| **54** | HG531520 | 1186-1479 | 1-1189 | TTGA | 3e^-144^ | 99% | 0.0 | 99% | 2 | NW_001471639.1  (Not determined) | CR1 (Non LTR) |
| **54** | HG531521 | 288-579 | 1-305 | GAACGCCCCTCCCAAAAC | 1e^-143^ | 99% | 3e^-134^ | 94% | 3 | NW_001471673.1  (Not determined) | Hitchcock (LTR) |
| **55** | HG531522 | 1-433 | 416-631 | ACCAACCCCAATCGAACC | 1e^-169^ | 97% | 1e^-101^ | 99% | 1 | NW_001471534.2  (Dystrophin) | --- |
| **55** | HG531523 | 1-136 /  332-691 | 119-335 /  684-877 | ACCAACCCCAATCGAACC /  GAAG /  CCAAAACC | 2e^-56^ /  2e^-169^ | 97% /  97% | 4e^-102^ /  2e^-94^ | 99% /  100% | 1 /  1 | NW_001471534.2  (Dystrophin)/  NW_001471534.2  (Dystrophin) | --- |
| **55** | HG531524 | 1-111 | 94-308 | ACCAACCCCAATCGAACC | 2e^-43^ | 96% | 7e^-100^ | 98% | 1 | NW_001471534.2  (Dystrophin) | --- |
| **57** | HG531525 | 487-622 | 1-495 | ATAATGTAC | 1e^-50^ | 94% | 0.0 | 100% | 6 | NW_003763812.1  (Metal transporter CNNM2 isoform 2) | --- |
| **57** | HG531526 | 47-311 | 1-60 | GAACGCCCCTCCCA | 5e^-101^ | 93% | 8e^-18^ | 95% | 3 | NW_001471667.1  (Similar to beige protein homolog) | --- |
| **57** | HG531527 | 403-670 | 1-416 | CTTGAACCCCCCTC | 4e^-100^ | 92% | 0.0 | 98% | 4 | NW_001471687.1  (Not determined) | --- |
| **57** | HG531528 | 1218-1435 | 1-1222 | GGTAG | 3e^-76^ | 92% | 0.0 | 96% | 3 | NW_001471668.1  (Not determined) | CR1 (Non LTR) |
| **57** | HG531529 | 75-344 | 1-88 | AACCTGAACCCCCC | 2e^-100^ | 92% | 6e^-32^ | 96% | 3 | NW_001471671.1  (Not determined) | CR1 (Non LTR) |
| **57** | HG531530 | 481-615 | 1-488 | ATAATGTA | 6e^-47^ | 93% | 0.0 | 99% | 1 | NW_001471545.1  (Hypothetical protein) | --- |
| **57** | HG531531 | 406-845 | 1-416 | GAAGCCCCCTC | 4e^-114^ | 91% | 0.0 | 98% | 4 | NW_001471687.1  (Not determined) | --- |
| **57** | HG531532 | 398-843 | 1-420 | GAACGCCCCTCCCAAAACCACAG | 6e^-175^ | 98% | 0.0 | 96% | 25 | NW_001471602.1  (Not determined) | --- |
| **58** | HG531533 | 487-793 | 1-495 | ATAATGTAC | 3e^-109^ | 90% | 0.0 | 99% | 6 | NW_003763812.1  (Metal transporter CNNM2 isoform 2) | --- |
| **58** | HG531534 | 487-880 | 1-495 | ATAATGTAC | 7e^-112^ | 91% | 0.0 | 99% | 6 | NW_003763812.1  (Metal transporter CNNM2 isoform 2) | --- |
| **58** | HG531535 | 193-482 | 1-194 | GA | 9e^-04^ | 68% | 3e^-53^ | 95% | 2 | NW_001471633.1  (Not determined) | --- |
| **58** | HG531536 | 345-636 | 1-86 /  87-360 | TTGAAGCCCCCTCCCA | 3e^-05^ | 70% | 3e^-33^ /  6e^-131^ | 96% /  98% | 15 / 2 | NW_001471461.1 (Not determined)/  NW_001471646.1  (Not determined) | --- |
| **58** | HG531537 | 267-558 | 1-268 | GA | 2e^-05^ | 71% | 5e^-125^ | 99% | 2 | NW_001471633.1  (Not determined) | --- |
| **58** | HG531538 | 43-333 | 1-58 | GAACCCCCCTCCCAAA | 3e^-07^ | 70% | 4e^-16^ | 93% | 4 | NW_001471688.1  (Not determined) | --- |
| **58** | HG531539 | 262-524 | 1-275 | GAACGCCCCTCCCA | 2e^-05^ | 84% | 8e^-52^ | 99% | 12 | NW_001471441.1  (Not determined) | --- |
| **58** | HG531540 | 194-327 | 1-202 | ATAATGTAC | 2e^-21^ | 100% | 2e^-95^ | 98% | 6 | NW_003763812.1 ([Metal transporter CNNM2 isoform 2](http://www.ncbi.nlm.nih.gov/nucleotide/358485087?report=gbwithparts&from=11324020&to=11422251&RID=7TJWX4S9015)) | --- |
| **58** | HG531541 | 1-150 | 145-335 | CATTAT | 9e^-21^ | 94% | 2e^-64^ | 87% | 2 | NW_001471633.1  (Not determined) | --- |
| **58** | HG531542 | 142-216 | 1-160 | GACCCCCCCTCCCAAAACC | 2e^-28^ | 98% | 5e^-68^ | 96% | 1 | NW_001471534.2  (pyruvate dehydrogenase kinase, isozyme 3) | CR1 (Non LTR) |
| **58** | HG531543 | 521-585 | 1-534 | AAATAATGTACGGG | 6e^-13^ | 100% | 0.0 | 99% | 8 | NW_001471740.1  (Not determined) | --- |
| **59** | HG531544 | 487-791 | 1-495 | ATAATGTAC | 2e^-149^ | 99% | 0.0 | 100% | 6 | NW_003763812.1  (Metal transporter CNNM2 isoform 2) | --- |
| **59** | HG531545 | 487-724 | 1-495 | ATAATGTAC | 9e^-40^ | 76% | 0.0 | 100% | 6 | NW_003763812.1  (Metal transporter CNNM2 isoform 2) | --- |
| **59** | HG531546 | 488-603 | 1-496 | ATAATGTAC | 7e^-34^ | 98% | 0.0 | 99% | 6 | NW_003763812.1  (Metal transporter CNNM2 isoform 2) | --- |
| **59** | HG531547 | 99-162 | 1-108 | CATAATGTAC | 4e^-23^ | 100% | 6e^-41^ | 96% | 8 | NW_001471740.2  (Not determined) | --- |
| **59** | HG531548 | 362-424 | 1-377 | ATAATGTACGGGGGAG | 3e^-20^ | 97% | 0.0 | 99% | 4 | NW_003763740.1  ([mineralocorticoid receptor](http://www.ncbi.nlm.nih.gov/nucleotide/358485161?report=gbwithparts&from=545458&to=739512&RID=7BHFA3CU014)) | CR1 (Non LTR) |
| **59** | HG531549 | 289-351 | 1-317 | ATAATGTACGGGGGAGATGCATGAATTTC | 2e^-20^ | 97% | 2e^-147^ | 97% | 13 | NW_003763912.1  (Not determined) | --- |
| **60** | HG531550 | 102-399 | 1-107 | AAAGTG | 8e^-145^ | 99% | 6e^-46^ | 99% | 19 | NW_003764121.1  (Putative polypeptide N-acetylgalactosa minyltransferase-like protein 3-like) | --- |
| **60** | HG531551 | 296-558 | 1-308 | GAACCCCCCTCCC | 6e^-129^ | 99% | 5e^-143^ | 97% | 1 | NW_003763493.1  (Not determined) | CR1 (Non LTR) |
| **60** | HG531552 | 304-593 | 1-313 | GAACCCCCCT | 5e^-143^ | 99% | 3e^-147^ | 97% | 3 | NW_001471668.2  (Not determined) | --- |
| **60** | HG531553 | 421-686 | 1-442 | TTTGACCCCCCCTCCCAAAACC | 2e^-111^ | 93% | 0.0 | 97% | 2 | NW_003763661.1  ([integrin alpha-8 precursor](http://www.ncbi.nlm.nih.gov/nucleotide/358485244?report=gbwithparts&from=19895458&to=19993969&RID=2AMKNJMV01N)) | --- |
| **60** | HG531554 | 137-203 | 1-146 | AAAATAATAA | 7e^-22^ | 97% | 1e^-63^ | 97% | 3 | NW_003763720.1  (Not determined ) | --- |
| **60** | HG531555 | 760-813 | 1-767 | TATAGTGT | 2e^-04^ | 80% | 0.0 | 98% | Not determined | NW_003764339.1  (Not determined) | --- |
| **60** | HG531556 | 204-328 | 1-210 | CATAATG | 1e^-46^ | 97% | 1e^-103^ | 100% | 2 | NW_003763661.1  (Not determined) | --- |
| **61** | HG531557 | 1-263 | 245-298 | CACCAACCCCAATCGAACC | 1e^-108^ | 94% | 8e^-11^ | 90% | 2 | NW_001471639.1  ([Similar to KIAA0222](http://www.ncbi.nlm.nih.gov/entrez/viewer.fcgi?val=118086752&db=Nucleotide&from=22937985&to=22995040&view=gbwithparts&RID=NEZUT5UX01R)) | --- |
| **61** | HG531558 | 1-263 | 247-332 | CCAACCCCAATCGAACC | 1e^-06^ | 78% | 9e^-30^ | 94% | 5 | NW_001471698.1  (UDP-N-acetyl-alpha-D-galactosamine:poly peptide N-acetyl galactosaminyltransferase-like 4) | --- |
| **62** | HG531559 | 1-286 | 270-465 | CCAACCCCAATYGAACC | 1e^-04^ | 89% | 3e^-75^ | 90% | 9 | NW_001471744.1  (Not determined) | CR1 (Non LTR) |
| **62** | HG531560 | 1-290 | 274-469 | CCAACCCCAATCGAACC | 3e^-132^ | 97% | 2e^-91^ | 98% | 9 | NW_001471744.1  (Not determined) | CR1 (Non LTR) |
| **62** | HG531561 | 81-363 | 1-89 | GACCGCCCC | 2e^-05^ | 86% | 3e^-36^ | 99% | 1 | NW_003763647.1  (Not determined) | CR1 (Non LTR) |
| **62** | HG531562 | 138-425 | 1-144 | AAGCTGA | 1e^-05^ | 94% | 4e^-61^ | 96% | 3 | NW_001471671.1  (Not determined) | CR1 (Non LTR) |
| **62** | HG531563 | 141-423 | 1-142 | GA | 4e^-06^ | 100% | 2e^-57^ | 95% | Z | NW_001488823.1  (Not determined) | CR1 (Non LTR) |
| **66** | HG531564 | 1-297 | 279-352 | CACCAACCCCAATCGAACC | 6e^-04^ | 88% | 6e^-20^ | 90% | Not determined | NW_001477862.1  (Not determined) | --- |
| **66** | HG531565 | 1-286 | 270-465 | CCAACCCCAATCGAACC | 8e^-139^ | 99% | 9e^-89^ | 97% | 9 | NW_001471744.1  (Not determined) | CR1 (Non LTR) |
| **66** | HG531566 | 1-308 | 292-487 | CCAACCCCAATCGAACC | 2e^-127^ | 94% | 2e^-91^ | 98% | 9 | NW_001471744.1  (Not determined) | CR1 (Non LTR) |
| **66** | HG531567 | 1-267 | 247-442 | CCAACCCCAATCGAACCCACC | 2e^-128^ | 99% | 6e^-91^ | 97% | 9 | NW_001471744.1  (Not determined) | CR1 (Non LTR) |
| **66** | HG531568 | 1-283 | 263-373 | TACACCAACCCCAATCGAACC | 4e^-06^ | 100% | 6e^-33^ | 90% | 1 | NW_001471554.1  (Not determined) | --- |
| **66** | HG531569 | 1-278 | 254-775 | ACCAACCCCAATCGAACCCCCAACT | 3e^-06^ | 82% | 0.0 | 89% | 3 | NW_001471673.1  (Not determined) | Hitchcock (LTR) |
| **66** | HG531570 | 1-271 | 253-306 | CACCAACCCCAATCGAACC | 2e^-04^ | 81% | 8e^-11^ | 90% | 2 | NW_001471639.1  ([Similar to KIAA0222](http://www.ncbi.nlm.nih.gov/entrez/viewer.fcgi?val=118086752&db=Nucleotide&from=22937985&to=22995040&view=gbwithparts&RID=RE1Y2ZVU01R)) | --- |
| **66** | HG531571 | 1-263 | 250-658 | ACCCCAATCGAACC | 1e^-04^ | 94% | 0.0 | 97% | 4 | NW_001471687.1  (fibroblast growth factor receptor 3) | --- |
| **67** | HG531572 | 1-394 | 373-496 | ACACACCAACCCCAATCGAACC | 5e^-161^ | 93% | 1e^-49^ | 96% | 2 | NW_003763686.1  (Not determined) | --- |
| **67** | HG531573 | 1-624 | 608-801 | CCAACCCCAATCGAACC | 0.0 | 94% | 3e^-73^ | 92% | 6 | NW_003763812.1  (Not determined) | --- |
| **67** | HG531574 | 1-214 | 208-332 | GAGATGC | 5e^-83^ | 93% | 1e^-47^ | 94% | 13 | NW_003763912.1  (Kv channel-interacting protein 1 isoform 2) | --- |
| **67** | HG531575 | 1-301 | 292-893 | GGGAGATGCA | 2e^-130^ | 95% | 0.0 | 96% | 4 | NW_003763740.1  (Collagen alpha-1(XXV) chain) | --- |
| **67** | HG531576 | 1-316 | 310-573 | GAGATGC | 1e^-145^ | 98% | 3e^-127^ | 99% | 17 | NW_003764072.1  (Not determined) | --- |
| **68** | HG531577 | 1-290 | 279-445 | CCCAATCGAACC | 2e-^06^ | 72% | 2e^-77^ | 98% | 2 | NW_001471642.1  (Not determined) | --- |
| **68** | HG531578 | 1-290 | 270-350 | TACACCAACCCCAATCGAACC | 5e^-05^ | 70% | 4e-28 | 95% | 1 | NW_003763650.1  ([NADP-dependent malic enzyme, mitochondrial](http://www.ncbi.nlm.nih.gov/nucleotide/358485255?report=gbwithparts&from=26967796&to=27085886&RID=7V1SWZ0R016)) | --- |
| **68** | HG531579 | 1-290 | 270-381 | TACACCAACCC CAATCGAACC | 4e^-07^ | 72% | 7e^-32^ | 89% | 1 | NW_003763650.1  ([NADP-dependent malic enzyme, mitochondrial](http://www.ncbi.nlm.nih.gov/nucleotide/358485255?report=gbwithparts&from=26967796&to=27085886&RID=7V1HN5U6014)) | --- |
| **68** | HG531580 | 1-288 | 271-476 | ACCAACCCCAATCGAACC | 3e^-54^ | 98% | 6e^-88^ | 96% | Z | AC200647.3  (Not determined) | --- |
| **68** | HG531581 | 1-294 | 278-473 | CCAACCCCAATCGAACC | 4e^-143^ | 99% | 2e^-91^ | 98% | 9 | NW_001471744.1  (Not determined) | CR1 (Non LTR) |
| **71** | HG531582 | 1-70 | 71-239 | ___ | 2e-08 | 84% | 1e^-77^ | 98% | 9 | NW_001471744.1  (Not determined) | CR1 (Non LTR) |
| **72** | HG531583 | 514-806 | 1-525 | GTTGAACCCCCC | 3e^-141^ | 99% | 0.0 | 99% | 6 | NW_003763812.1  (Metal transporter CNNM2 isoform 2) | --- |
| **73** | HG531584 | 91-306 | 1-103 | CCCCTCCCAAAAC | 2e^-100^ | 99% | 9e^-29^ | 90% | Z | AC216895.3  (Not determined) | CR1 (Non LTR) |
| **74** | HG531585 | 74-366 | 1-92 | TGACCCCCCCTCCCAAAAC | 7e^-145^ | 99% | 3e^-29^ | 91% | 12 | NW_003763892.1  (Dedicator of cytokinesis protein 3) | --- |
| **74** | HG531586 | 376-668 | 1-389 | TGAACCCCCCTCCC | 5e^-144^ | 99% | 0.0 | 99% | 18 | NW_003764078.1  (Uncharacterized protein LOC100858297) | --- |
| **74** | HG531587 | 181-475 | 1-200 | TTTGAACCCCCCTCCCAAAA | 2e^-146^ | 99% | 4e^-87^ | 96% | 10 | NW_003763854.1  (Immunoglobulin superfamily DCC subclass member 4-like) | --- |
| **74** | HG531588 | 388-679 | 1-395 | GAAGCCCC | 1e^-144^ | 99% | 0.0 | 99% | 14 | NW_003763931.1  (Ubiquitin carboxyl-terminal hydrolase 22-A) | --- |
| **74** | HG531589 | 527-818 | 1-534 | GAACGCCC | 4e^-140^ | 98% | 0.0 | 96% | Z | AC231413.2  (Not determined) | Z-REP (Sat) /  CR1 (Non LTR) |
| **74** | HG531590 | 382-673 | 1-389 | GAAGACCC | 1e^-144^ | 99% | 0.0 | 96% | Z | NW_003764323.1  (Not determined) | Z-REP (Sat) |

**Table S3.** Vertical transfer of minicircle sequences of kDNA from *Trypanosoma cruzi* into the *Gallus* gallus germ line cells.

| Ave | Accession # | kDNA | *G. gallus* DNA | Intermediate recombination site | KDNA *E-value* | kDNA  Identity | *G. gallus*  *E-value* | *G. gallus*  Identity | *G. gallus* Chromosome | *Locus* | *G. gallus* repeats |
| --- | --- | --- | --- | --- | --- | --- | --- | --- | --- | --- | --- |
| **1** | HG531591 | 1-93 | 76-291 | ACCAACCCCAATCGAACC | 4e^-31^ | 96% | 6e^-101^ | 98% | 1 | NW_001471534.2 (Dystrophin) | --- |
| **1** | HG531592 | 1-263 | 246-461 | ACCAACCCCAATCGAACC | 9e^-11^ | 100% | 3e^-100^ | 98% | 1 | NW_001471534.2 (Dystrophin) | --- |
| **1** | HG531593 | 415-742 | 1-435 | TATAATGTACGGGTGAGATGC | 3e^-20^ | 77% | 0.0 | 98% | 5 | NW_003763785.1 (Transcription Factor SOX-6) | --- |
| **1** | HG531594 | 185-470 | 1-197 | GACGCCCCCTCCC | 2e^-140^ | 100% | 2e^-84^ | 95% | 2 | NW_003763668.1 (Protein PTHB1) | CR1 (Non LTR) |
| **1** | HG531595 | 189-468 | 1-197 | CCCCCTCCC | 9e^-09^ | 81% | 2e^-77^ | 92% | 2 | NW_003763668.1 (Protein PTHB1) | CR1 (Non LTR) |
| **1** | HG531596 | 173-459 | 1-185 | GACGCCCCCTCCC | 3e^-139^ | 100% | 2e^-84^ | 98% | 3 | NW_001471668.2 (Probable Palmitoyltransferase ZDHHC14 isoforms 1 or 2) | CR1 (Non LTR) |
| **1** | HG531597 | 76-130 | 1-104 | ACCAATTTCACATCACAACCCAAACCCAT | 7e^-19^ | 100% | 3e^-31^ | 91% | 4 | NW_003763735.1 (Not determined) | CR1 (Non LTR) |
| **1** | HG531598 | 53-346 | 1-63 | AAGACCGCCCC | 3e^-137^ | 98% | 9e^-24^ | 100% | 5 | NW_003763785.1 (Not determined) | --- |
| **1** | HG531599 | 1-719 | 698-1204 | TACACCAACCCCAATCGAACC | 0.0 | 89% | 0.0 | 98% | 1 | NW_003763650.1 (NADP-dependent malic enzyme, mitochondrial) | --- |
| **1** | HG531600 | 1-477 | 456-963 | ATACACCAACCCCAATCGAACC | 0.0 | 96% | 0.0 | 98% | 1 | NW_003763650.1 (NADP-dependent malic enzyme, mitochondrial) | --- |
| **1** | HG531601 | 1-309 | 292-793 | ACCAACCCCAATCGAACC | 1e^-128^ | 37% | 0.0 | 97% | 1 | NW_003763650.1 (NADP-dependent malic enzyme, mitochondrial) | --- |
| **2** | HG531602 | 1-250 | 233-393 | ACCAACCCCAATCGAACC | 3e^-67^ | 83% | 6e-71 | 98% | 3 | NW_001471673.2 (Not determined) | Hitchcock (LTR) |
| **2** | HG531603 | 1-192 | 178-260 | GTACGGGTGAGATGC | 9e^-79^ | 95% | 5e^-25^ | 92% | 1 | NW_003763482.1 (Not determined) | --- |
| **2** | HG531604 | 1-328 | 292-794 | ACCAACCCCAATCGAACC | 7e^-131^ | 95% | 0.0 | 98% | 1 | NW_003763650.1 (NADP-dependent malic enzyme, mitochondrial) | --- |
| **2** | HG531605 | 1-265 | 244-749 | ATACACCAACCCCAATCGAACC | 4e^-102^ | 92% | 0.0 | 97% | 1 | NW_003763650.1 (NADP-dependent malic enzyme, mitochondrial) | --- |
| **3** | HG531606 | 1-773 | 755-970 | ACCAACCCCAATCGAACC | 0.0 | 97% | 1e^-96^ | 97% | 1 | NW_001471534.2 (Dystrophin) | --- |
| **3** | HG531607 | 898-1241 | 1-914 | GAACCCCCCTCCCAAAA | 3e^-75^ | 96% | 0.0 | 97% | Not determined | NW_003780056.1 (Not determined) | --- |
| **3** | HG531608 | 108-810 | 1-121 | CCCTTCCCAAAACC | 1e^-86^ | 94% | 3e^-40^ | 98% | 14 | NW_001471454.1\|Gga_WGA202_2 (Similar to KIAA1691 protein) | --- |
| **3** | HG531609 | 1-79 | 58-167 | ATACACCAACCCCAATCGAACC | 7e^-14^ | 92% | 4e^-37^ | 92% | 1 | NW_003763650.1 (NADP-dependent malic enzyme, mitochondrial) | --- |
| **3** | HG531610 | 1-83 | 63-583 | ACCAACCCCAATCGAACCCCA | 1e^-23^ | 92% | 0.0 | 99% | 3 | NW_001471673.2 (Not determined) | Hitchcock (LTR) |
| **3** | HG531611 | 1-83 | 55-125 | ACCATAACACCAACCCCAATCGAACCCCA | 3e^-24^ | 92% | 3e^-06^ | 92% | 28 | NW_003764302.1 (Not determined) | --- |
| **3** | HG531612 | 1-141 | 107-293 | AATATAACTCTAATTACACCAACCCCAATCGAACC | 2e^-42^ | 95% | 4e^-90^ | 97% | 2 | AB556723.1 (Centromere 2 repeat sequence) | --- |
| **3** | HG531613 | 1-263 | 244-747 | ACACCAACCCCAATCGAACC | 1e^-120^ | 97% | 0.0 | 97% | 1 | NW_003763650.1 (NADP-dependent malic enzyme, mitochondrial) | --- |
| **4** | HG531614 | 1-62 | 53-317 | CAATCGAACC | 5e^-20^ | 98% | 2e^-125^ | 98% | 1 | NW_003763650.1 (Teneurin-4) | CR1 (Non LTR) |
| **4** | HG531615 | 1-73 | 53-153 | TACACCAACCCCAATCGAACC | 2e^-20^ | 96% | 2e^-33^ | 96% | 1 | NW_003763650.1 (NADP-dependent Malic enzime, mitochondrial) | --- |
| **5** | HG531616 | 1-94 | 77-292 | ACCAACCCCAATCGAACC | 4e^-34^ | 99% | 2e^-99^ | 98% | 1 | NW_001471534.2 (Dystrophin) | --- |
| **5** | HG531617 | 1-466 | 249-664 | ACCAACCCCAATCGAACC | 4e^-146^ | 97% | 1e^-100^ | 98% | 1 | NW_001471534.2 (Dystrophin) | --- |
| **5** | HG531618 | 1-170 | 133-368 | ACCAACCCCAATCGAACTACACCAACCCCAATCGAACC | 5e^-27^ | 87% | 6e^-102^ | 99% | 1 | NW_001471534.2 (Dystrophin) | --- |
| **5** | HG531619 | 1-264 | 246-461 | ACCAACCCCAATCGAACC | 1e^-125^ | 98% | 8e^-102^ | 99% | 1 | NW_001471534.2 (Dystrophin) | --- |
| **5** | HG531620 | 117-185 | 1-127 | TACACCAACCC | 7e^-16^ | 96% | 2e^-41^ | 91% | Not determined | NW_003771790.1 (Not determined) | --- |
| **5** | HG531621 | 1-245 | 222-514 | GTCGAACGCCCCTCCCAAAACCAA | 4e^-144^ | 99% | 3e^-121^ | 99% | 15 | NW_003763985.1 (Not determined) | --- |
| **5** | HG531622 | 1-250 | 229-735 | ATACACCAACCCCAATCGAACC | 1e^-102^ | 94% | 0.0 | 98% | 1 | NW_003763650.1 (NADP-dependent malic enzyme, mitochondrial) | --- |
| **5** | HG531623 | 1-247 | 239-744 | ATACACCAACCCCAATCGAACC | 6e^-93^ | 92% | 0.0 | 98% | 1 | NW_003763650.1 (NADP-dependent malic enzyme, mitochondrial) | --- |
| **11** | HG531624 | 1-93 | 76-291 | ACCAACCCCAATCGAACC | 5e^-32^ | 98% | 1e^-96^ | 99% | 1 | NW_001471534.2 (Dystrophin) | --- |
| **11** | HG531625 | 263-556 | 1-286 | CTGAACCCCCCTCCCAAAACCGCA | 5e^-143^ | 99% | 3e^-120^ | 94% | 2 | NW_003763673.1 (Not determined) | --- |
| **11** | HG531626 | 153-444 | 1-162 | GAACGCCCCT | 1e^-143^ | 99% | 9e^-76^ | 99% | 6 | NW_003763812.1 (Similar to atractin 1, isoform 2) | --- |
| **11** | HG531627 | 58-350 | 1-73 | TGAAAGCCCCCTCCCAA | 5e^-147^ | 100% | 5e^-21^ | 90% | 1 | NW_003763464.1 (Not determined) | --- |
| **11** | HG531628 | 1-39 | 39-330 | G | 7e^-145^ | 99% | 2e^-12^ | 94% | 4 | NW_003763735.1 (Not determined) | --- |
| **11** | HG531629 | 299-600 | 1-321 | CAGGAACGATGAAGCCCCCTCCC | 8e^-147^ | 100% | 3e^-152^ | 98% | 6 | NW_003763812.1 (Not determined) | CR1 (Non LTR) |
| **11** | HG531630 | 1-231 | 269-716 | ATACACCAACCCCAATCGAACC | 1e^-102^ | 98% | 0.0 | 98% | 1 | NW_003763650.1 (NADP-dependent malic enzyme, mitochondrial) | --- |
| **11** | HG531631 | 1-187 | 183-685 | ACCAACCCCAATCGAACC | 3e^-72^ | 95% | 0.0 | 98% | 1 | NW_003763650.1 (NADP-dependent malic enzyme, mitochondrial) | --- |
| **11** | HG531632 | 1-154 | 134-639 | TACACCAACCCCAATCGAACC | 2e^-23^ | 80% | 0.0 | 98% | 1 | NW_003763650.1 (NADP-dependent malic enzyme, mitochondrial) | --- |
| **13** | HG531633 | 1-167 | 150-365 | ACCAACCCCAATCGAACC | 4e^-47^ | 91% | 3e^-100^ | 98% | 1 | NW_001471534.2 (Dystrophin) | --- |
| **13** | HG531634 | 1-75 | 51-274 | GGAGATGCACAACCCCAATCGAACC | 2e^-19^ | 94% | 4e^-102^ | 99% | 1 | NW_001471534.2 (Dystrophin) | --- |
| **13** | HG531635 | 1-284 | 272-684 | CCCCAATCGAACC | 2e^-04^ | 79% | 0.0 | 98% | 17 | NW_003764074.1 (Adenylate kinase 8) | --- |
| **13** | HG531636 | 1-169 | 153-348 | CCAACCCCAATCGAACC | 6e^-57^ | 96% | 1e^-91^ | 98% | 9 | NW_003763840.1 (Not determined) | CR1 (Non LTR) |
| **13** | HG531637 | 1-276 | 267-391 | CAATCGAACC | 2e^-70^ | 91% | 9e^-56^ | 99% | 5 | NW_003763785.1 (Tetraspanin 18) | --- |
| **13** | HG531638 | 1-226 | 205-709 | ATACACCAACCCCAATCGAACC | 2e^-100^ | 97% | 0.0 | 97% | 1 | NW_003763650.1 (NADP-dependent malic enzyme, mitochondrial) | --- |
| **13** | HG531639 | 1-104 | 94-600 | ATACACCAACCCCAATCGAACC | 9e^-27^ | 94% | 0.0 | 98% | 1 | NW_003763650.1 (NADP-dependent malic enzyme, mitochondrial) | --- |
| **14** | HG531640 | 1-151 | 134-349 | ACCAACCCCAATCGAACC | 8e^-50^ | 94% | 1e^-98^ | 97% | 1 | NW_001471534.2 (Dystrophin) | --- |
| **14** | HG531641 | 1-265 | 248-463 | ACCAACCCCAATCGAACC | 1e^-99^ | 92% | 3e^-100^ | 98% | 1 | NW_001471534.2 (Dystrophin) | --- |
| **14** | HG531642 | 1-169 | 152-367 | ACCAACCCCAATCGAACC | 1e^-48^ | 92% | 5e^-97^ | 97% | 1 | NW_001471534.2 (Dystrophin) | --- |
| **14** | HG531643 | 115-400 | 1-124 | GAACCCCCCT | 2e^-140^ | 99% | 1e^-40^ | 91% | 13 | NW_003763912.1 (Not determined) | --- |
| **14** | HG531644 | 132-417 | 1-140 | GACCCCCCC | 2e^-140^ | 99% | 6e^-39^ | 86% | Not determined | BX640540.3 (Not determined) | CR1 (Non LTR) |
| **14** | HG531645 | 399-688 | 1-420 | GAACGCCCCTCCCAAAACCAAA | 6e-^137^ | 100% | 0.0 | 97% | 25 | NW_003764242.1 (Not determined) | --- |
| **17** | HG531646 | 1-486 | 467-683 | ACACCAACCCCAATCGAACC | 2e^-27^ | 80% | 6e^-99^ | 98% | 1 | NW_001471534.2 (Dystrophin) | --- |
| **17** | HG531647 | 1-317 | 300-515 | ACCAACCCCAATCGAACC | 7e^-57^ | 98% | 5e^-99^ | 98% | 1 | NW_001471534.2 (Dystrophin) | --- |
| **17** | HG531648 | 78-177 | 1-95 | GAAGGCCCCTCCCAAAAC | 5e^-29^ | 89% | 1e^-31^ | 93% | 14 | NW_003763931.1 (xylosyltransferase 1) | --- |
| **17** | HG531649 | 204-284 | 1-210 | CATAATG | 4e^-27^ | 96% | 1e^-103^ | 100% | 2 | NW_003763661.1 (Not determined) | --- |
| **17** | HG531650 | 1-601 | 582-1086 | TACACCAACCCCAATCGAACC | 0.0 | 97% | 0.0 | 98% | 1 | NW_003763650.1 (NADP-dependent malic enzyme, mitochondrial) | --- |
| **18** | HG531651 | 1-201 | 91-300 | TACACCAACCCCAATCGAACC | --- | --- | 3e^-35^ | 91% | 1 | NW_003763650.1 (NADP-dependent malic enzyme, mitochondrial) | --- |
| **18** | HG531652 | 1-258 | 238-337 | TACACCAACCCCAATCGAACC | --- | --- | 1e^-35^ | 94% | 1 | NW_003763650.1 (NADP-dependent malic enzyme, mitochondrial) | --- |
| **18** | HG531653 | 1-278 | 267-433 | CCCAATCGAACC | 9e^-126^ | 96% | 1e^-74^ | 98% | 2 | NW_003763680.1  (Receptor-type tyrosine-protein phosphatase mu-like) | --- |
| **18** | HG531654 | 39-326 | 1-49 | GACGGCCCCTC | 1e^-140^ | 99% | 2e^-12^ | 94% | 4 | NW_003763735.1 (Not determined) | --- |
| **18** | HG531655 | 264-551 | 1-277 | GAACGCCCCTCCCA | 2e^-141^ | 99% | 2e^-135^ | 99% | 12 | NW_003763892.1 (Not determined) | --- |
| **18** | HG531656 | 30-317 | 1-37 | GAAGGCCC | 1e^-141^ | 99% | 2e^-07^ | 97% | 5 | NW_003763748.1 (Not determined) | --- |
| **18** | HG531657 | 1-288 | 268-772 | TACACCAACCCCAATCGAACC | 1e^-134^ | 99% | 0.0 | 97% | 1 | NW_003763650.1 (NADP-dependent malic enzyme, mitochondrial) | --- |
| **19** | HG531658 | 1-209 | 192-406 | ACCAACCCCAATCGAACC | 7e^-49^ | 88% | 4e^-99^ | 98% | 1 | NW_001471534.2 (Dystrophin) | --- |
| **19** | HG531659 | 1-607 | 588-637 | ACACCAACCCCAAACGAACC | 0.0 | 84% | 5e^-05^ | 86% | 1 | NW_003763496.1 (Not determined) | --- |
| **19** | HG531660 | 1-150 | 146-798 | ATGCA | 8e^-54^ | 94% | 0.0 | 98% | 3 | NW_001471673.2 (Peptidyl-prolyl cis-trans isomerase FKBP1B) | CR1 (Non LTR) |
| **19** | HG531661 | 1-265 | 237-666 | GATACACATACACCAACCCCAATCGAACC | 7e^-104^ | 93% | 0.0 | 96% | 17 | NW_003764074.1 (Adenylate kinase 8) | --- |
| **19** | HG531662 | 1-433 | 417-624 | CCAACCCCAATCGAACC | 3e^-171^ | 92% | 4e^-94^ | 97% | 5 | NW_003763785.1 (Inositol-triphosfate 3-kinase A) | --- |
| **19** | HG531663 | 141-405 | 1-159 | GACCCCCCCTCCCAAAACC | 1e^-105^ | 94% | 1e^-67^ | 96% | 1 | NW_001471534.2 ([Pyruvate dehydrogenase [lipoamide]] kinase isozyme 3, mitochondrial) | CR1 (Non LTR) |
| **19** | HG531664 | 151-757 | 1-169 | GACGCCCCCTCCCAAAACC | 0.0 | 84% | 1e^-51^ | 88% | Not determined | NW_003776158.1 (Not determined) | CR1 (Non LTR) |
| **20** | HG531665 | 1-98 | 79-272 | CAACCCCAATCGAACCCACC | 1e^-33^ | 95% | 1e^-89^ | 98% | 9 | NW_003763840.1 (Not determined) | CR1 (Non LTR) |
| **20** | HG531666 | 1-39 | 16-105 | AACCATAATGTACGGGTGAGATGC | 9e^-10^ | 100% | 2e^-25^ | 92% | 1 | NW_003763482.1 (Not determined | --- |
| **20** | HG531667 | 1-241 | 224-419 | CCAACCCCAATCGAACCC | 6e^-71^ | 98% | 2e^-90^ | 98% | 9 | NW_003763840.1 (Not determined) | CR1 (Non LTR) |
| **22** | HG531668 | 31-308 | 1-38 | CCCCTCCC | 2e^-133^ | 99% | 8e^-05^ | 89% | 4 | NW_003763740.1 (Not determined) | --- |
| **22** | HG531673 | 303-585 | 1-315 | GACGCCCCCTCCC | 1e^-138^ | 96% | 2e^-143^ | 96% | 1 | NW_003763493.1 (Not determined) | CR1 (Non LTR) |
| **22** | HG531669 | 36-318 | 1-43 | GACGGCCC | 2e^-05^ | 89% | 2e^-11^ | 98% | 27 | NW_003764296.1 (Amiloride-sensitive cation channel 1, neuronal) | --- |
| **22** | HG531670 | 74-346 | 1-82 | GAACGCCCCTCCCAAAACC | 3e^-137^ | 99% | 1e^-27^ | 96% | 21 | NW_003764143.1 (Cytidine deaminase) | --- |
| **22** | HG531671 | 278-533 /  835-927 | 1-277 /  522-846 | GAACGCCCTCCCA/  GGGAGAAGAGTT/  GACGCCCCTCCC | 3e^-97^ /  1e^-32^ | 93% /  98% | 7e^-132^ /  1e^-154^ | 98% /  98% | 12 /  6 | NW_003763892.1 (Not determined) /  NW_003763812.1 (Not determined) | --- /  CR1 (Non LTR) |
| **22** | HG531672 | 43-318 | 1-45 | TCC | 7e^-62^ | 78% | 0.012 | 83% | 5 | NW_003763748.1 (Not determined) | --- |
| **24** | HG531674 | 1-130 | 120-219 | TACACCAACCCCAATCGAACC | 6e^-53^ | 97% | 2e^-36^ | 92% | 1 | NW_003763650.1 (NADP-dependent malic enzyme, mitochondrial) | --- |
| **24** | HG531675 | 1-201 | 195-296 | ATACACCAACCCCAATCGAACC | 1e^-71^ | 91% | 2e^-36^ | 94% | 1 | NW_003763650.1 (NADP-dependent malic enzyme, mitochondrial) | --- |
| **24** | HG531676 | 1-130 | 120-295 | CCATCGAACC | 3e^-53^ | 97% | 3e^-73^ | 97% | 2 | NW_003763680.1  (Receptor-type tyrosine-protein phosphatase mu-like) | --- |
| **24** | HG531677 | 1-150 | 134-683 | CCAACCCCAATCGAACC | 9e^-46^ | 90% | 0.0 | 99% | 5 | NW_003763785.1 (Not determined) | --- |
| **24** | HG531678 | 1-264 | 242-437 | CCAACCCCAATCGAACCCACCAT | 4e^-79^ | 86% | 2e^-91^ | 98% | 9 | NW_003763840.1 (Not determined) | CR1 (Non LTR) |
| **24** | HG531679 | 1-216 | 200-395 | CCAACCCCAATCGAACC | 1e^-72^ | 91% | 1e^-91^ | 98% | 9 | NW_003763840.1 (Not determined) | CR1 (Non LTR) |
| **24** | HG531680 | 1-283 | 269-351 | GTACGGGTGAGATGC | 5e^-109^ | 92% | 7e^-25^ | 92% | 1 | NW_003763482.1 (Not determined) | --- |
| **26** | HG531681 | 1-158 | 141-250 | TACACCAACCCCAATCGA | --- | --- | 1e^-33^ | 90% | 1 | NW_003763650.1 (NADP-dependent malic enzyme, mitochondrial) | --- |
| **26** | HG531682 | 1-265 | 249-444 | CCAACCCCAATCGAACC | 1e^-41^ | 94% | 2e^-90^ | 98% | 9 | NW_003763840.1 (Not determined) | CR1 (Non LTR) |
| **28** | HG531683 | 101-392 | 1-112 | GAAGGCCCCTCC | 8e-145 | 99% | 1e-47 | 98% | 1 | NW_003763482.1 (Not determined) | --- |
| **28** | HG531684 | 98-387 | 1-104 | GACCGCC | 1e^-141^ | 99% | 2e^-44^ | 99% | 5 | NW_001471710.2 (RAC-alpha serine/threonine-protein kinase) | --- |
| **28** | HG531685 | 264-555 | 1-277 | GAACGCCCCTCCCA | 1e^-143^ | 99% | 3e^-134^ | 99% | 12 | NW_003763892.1 (Not determined) | --- |
| **28** | HG531686 | 265-556 | 1-286 | GAAGCCCCCTCCCAAAACCACA | 1e^-144^ | 99% | 8e^-128^ | 96% | 2 | NW_003763673.1 (Not determined) | --- |
| **28** | HG531687 | 62-353 | 1-74 | GAAGCCCCTCCC | 9e^-144^ | 99% | 2e^-25^ | 96% | 9 | NW_001471743.2 (Uncharacterized protein LOC769332) | --- |
| **28** | HG531688 | 362-653 | 1-369 | GAAGCCCC | 4e^-145^ | 99% | 0.0 | 99% | 4 | NW_003763739.1 (Not determined) | --- |
| **28** | HG531689 | 52-343 | 1-59 | GAAGGCCC | 2e^-138^ | 98% | 3e^-17^ | 93% | 5 | NW_003763785.1 (Not determined) | --- |
| **28** | HG531690 | 175-463 | 1-180 | GAACCC | 9e^-139^ | 99% | 4e^-87^ | 100% | 12 | NW_003763892.1 (Not determined) | --- |
| **36** | HG531691 | 68-417 | 1-91 | CACATAATGTACGGGGGAGATGCA | 5e^-09^ | 95% | 2e^-33^ | 97% | 15 | NW_001471461.2 (Not determined) | --- |
| **36** | HG531692 | 235-526 | 1-253 | GAACGCCCCTCCCAAAACC | 1e^-144^ | 99% | 1e^-119^ | 98% | 15 | NW_003763985.1 (Not determined) | --- |
| **36** | HG531693 | 382-673 | 1-397 | GAAGGCCCCTCCCAAA | 1e^-144^ | 99% | 0.0 | 97% | Z | NW_003764323.1 (Not determined) | Z-Rep (Sat) |
| **36** | HG531694 | 394-690 | 1-414 | TTTGCGAAGGCCCCTCCCAAA | 2e^-143^ | 99% | 0.0 | 98% | 11 | NW_003763865.1 (Not determined) | Z-Rep (Sat)/ CR1 (Non LTR) |
| **36** | HG531695 | 268-559 | 1-285 | GACCCCCCCTCCCAAAAC | 1e^-143^ | 99% | 6e^-136^ | 98% | 2 | NW_003763664.1 (Not determined) | --- |
| **36** | HG531696 | 382-673 | 1-397 | GAAGGCCCCTCCCAAA | 3e^-140^ | 99% | 0.0 | 96% | Z | NW_003764324.1 (Not determined) | Z-Rep (Sat) |
| **36** | HG531697 | 620-911 | 1-638 | GAAGGCCCCTCCCAAAAC | 3e^-143^ | 99% | 0.0 | 96% | 2 | NW_003763668.1 (E3 ubiquitin-protein ligase HECW1) | --- |
| **36** | HG531698 | 404-695 | 1-421 | GAACGCCCCTCCCAAAAC | 2e^-143^ | 99% | 0.0 | 95% | 1 | NW_003763449.1 (Not determined) | --- |
| **36** | HG531699 | 156-447 | 1-164 | GACGGCCCC | 9e^-145^ | 99% | 4e^-74^ | 98% | 1 | NW_003763484.1 (Not determined) | --- |
| **36** | HG531700 | 264-555 | 1-277 | GAAGGCCCCTCCCA | 3e^-139^ | 98% | 2e^-135^ | 99% | 12 | NW_003763892.1 (Not Not determined) | --- |
| **38** | HG531701 | 1-276 | 255-365 | CTACACCAACCCCAATCGAACC | 5e^-46^ | 77% | 3e^-36^ | 92% | 1 | NW_003763650.1 (NADP-dependent malic enzyme, mitochondrial) | --- |
| **38** | HG531702 | 1-133 | 117-667 | CCAACCCCAATCGAACC | 5e^-49^ | 99% | 0.0 | 99% | 5 | NW_003763785.1 (Not determined) | --- |
| **39** | HG531703 | 1-109 | 76-281 | CACCAACCCCAATCGAACCGTTTTCTCCCCTAAA | 7e^-30^ | 89% | 2e^-93^ | 97% | Not determined | NW_003779328.1 (Not determined) | GGXOI (Simple Sat) |
| **39** | HG531704 | 1-111 | 99-515 | CCCCAATCGAACC | 2e^-21^ | 97% | 0.0 | 97% | 17 | NW_003764074.1 (Adenylate kinase 8) | --- |
| **39** | HG531705 | 1-197 | 194-599 | ACCA | 5e^-49^ | 83% | 0.0 | 98% | 17 | NW_003764074.1 (Adenylate kinase 8) | --- |
| **39** | HG531706 | 629-827 | 1-641 | GAAGCCCCCTCCC | 4e^-77^ | 97% | 0.0 | 97% | 13 | NW_003763913.1 (Solute carrier Family 25 member 48) | --- |
| **39** | HG531707 | 427-675 | 1-533 | ATAATG | 3e^-39^ | 87% | 0.0 | 99% | 9 | NW_001471743.2 (Not determined) | --- |
| **39** | HG531708 | 160-308 | 1-163 | CATA | 2e^-38^ | 92% | 8e^-68^ | 96% | 1 | NW_001471534.2 ([Pyruvate dehydrogenase [lipoamide]] kinase Isozyme 3, mitochondrial) | CR1 (Non LTR) |
| **39** | HG531709 | 1-42 | 33-276 | GAGATGCATA | 3e^-09^ | 98% | 1e^-116^ | 98% | 2 | NW_003763661.1 (Not determined) | --- |
| **40** | HG531710 | 1-149 | 129-238 | TACACCAACCCCAATCGAACC | --- | --- | 2e^-35^ | 91% | 1 | NW_003763650.1 (NADP-dependent malic enzyme, mitochondrial) | --- |
| **40** | HG531711 | 1-130 | 110-219 | TACACCAACCCCAATCGAACC | 2e^-54^ | 98% | 2e^-36^ | 92% | 1 | NW_003763650.1 (NADP-dependent malic enzyme, mitochondrial) | --- |
| **40** | HG531712 | 1-100 | 85-704 | GTACGGGGGAGATGCA | 5e^-30^ | 91% | 0.0 | 98% | 12 | NW_003763903.1 (forkhead box protein P1) | --- |
| **40** | HG531713 | 68-250 | 1-93 | CACATAATGTACGGGTGAGATGCATG | 2e^-80^ | 97% | 1e^-33^ | 96% | 15 | NW_001471461.2 (Not determined) | --- |
| **40** | HG531714 | 256-465 | 1-289 | TAGCACAATCCGACCCCCCCTCCCAAAAC | 4e^-80^ | 98% | 9e-^133^ | 98% | 2 | NW_003763664.1 (Not determined) | --- |
| **40** | HG531715 | 1-100 | 93-342 | GAGATGCT | 1e^-34^ | 95% | 3e^-119^ | 98% | 20 | NW_003764128.1 (Not determined) | --- |
| **42** | HG531716 | 185-475 | 1-197 | GACGCCCCCTCCC | 5e^-142^ | 99% | 2e^-89^ | 97% | 2 | NW_003763668.1 (Protein PTHB1) | CR1 (Non LTR) |
| **42** | HG531717 | 50-396 | 1-97 | GAACCTCCCCTGGCACAACTTCAACCATCACCTCTCCTTCTGTTGCTG | 2e^-122^ | 99% | 2e^-32^ | 93% | Z | NW_003764322.1 (Not determined) | CR1 (Non LTR) |
| **42** | HG531718 | 185-474 | 1-197 | GACGCCCCCTCCC | 1e^-142^ | 100% | 1e^-86^ | 96% | 2 | NW_003763668.1 (Protein PTHB1) | CR1 (Non LTR) |
| **44** | HG531719 | 1-200 | 183-274 | ACCAACCCCAATCGAACC | --- | --- | 3e-34 | 96% | 1 | NW_003763650.1 (NADP-dependent malic enzyme, mitochondrial) | --- |
| **44** | HG531720 | 1-132 | 112-307 | CCAACCCCAATCGAACCCACC | 3e^-35^ | 91% | 1e^-90^ | 98% | 9 | NW_003763840.1 (Not determined) | CR1 (Non LTR) |
| **44** | HG531721 | 1-150 | 139-373 | GGGTGAGATGCA | 4e^-60^ | 96% | 2e^-114^ | 99% | 2 | NW_003763693.1 (Protein tyrosine phosphatase type IVA 3 isoform 1) | --- |
| **49** | HG531722 | 1-179 | 173-431 | GAGATGC | 9e^-50^ | 93% | 4e^-130^ | 100% | 5 | NW_003763785.1 (Not determined) | --- |
| **49** | HG531723 | 1-102 | 99-755 | ATGC | 1e^-25^ | 87% | 0.0 | 98% | 3 | NW_001471673.2 (Peptidyl-prolyl cis-trans isomerase FKBP1B) | CR1 (Non LTR) |
| **49** | HG531724 | 1-265 | 246-297 | ACCAACCCCAATCGAACCC | 5e^-127^ | 99% | 1e^-08^ | 87% | Z | NW_001488830.2 (Succinyl-CoA: 3-ketoacid-coenzyme A transferase 1, mitochondrial) | --- |
| **49** | HG531725 | 1-442 | 422-786 | TATAATGTACGGGGGAGATGC | 2e^-98^ | 83% | 0.0 | 97% | 5 | NW_003763785.1 (Transcription Factor SOX-6) | --- |
| **50** | HG531726 | 264-555 | 1-277 | GAACCCCCCTCCCA | 2e^-117^ | 93% | 3e^-134^ | 99% | 12 | NW_003763892.1 (Not determined) | --- |
| **50** | HG531727 | 383-494 | 1-104 | TATAATGTACGGGTGAGATGCA | 7e^-39^ | 94% | 0.0 | 98% | 3 | NW_001471673.2 (Mitochondrial peptide methionine sulfoxide reductase) | CR1 (Non LTR) |
| **50** | HG531728 | 1-76 | 58-172 | GCAGATAATGTACGGGTGA | 3e^-45^ | 97% | 1e^-23^ | 92% | 11 | NW_003763881.1 (72kDa type IV collagenase preproprotein) | --- |
| **51** | HG531729 | 1-296 | 279-475 | CCAACCCCAATCGAACCC | 7e-^134^ | 98% | 7e^-90^ | 98% | 9 | NW_003763840.1 (Not determined) | CR1 (Non LTR) |
| **51** | HG531730 | 1-84 / 271-347 | 85-273 | TCC | 4e^-24^ / 3e^-26^ | 92%/ 94% | 1e^-12^ | 72% | 12 | NW_003763903.1 (Uncharacterized protein LOC416093) | --- |
| **53** | HG531731 | 185-314 | 1-197 | GACGCCCCCTCCC | 1e^-51^ | 98% | 2e^-89^ | 97% | 2 | NW_003763668.1 (Protein PTHB1) | CR1 (Non LTR) |
| **53** | HG531732 | 318-448 | 1-329 | GAAGGCCCCTC | 5e^-53^ | 97% | 1e^-156^ | 98% | 20 | NW_003764126.1 (Retinoblastoma-like protein 1) | CR1 (Non LTR) |
| **53** | HG531733 | 121-425 | 1-123 | AAC | 4e^-67^ | 83% | 3e^-06^ | 71% | Z | NW_003764319.1 (Not determined) | --- |
| **55** | HG531734 | 1-339 | 318-429 | ATACACCAACCCCAATCGAACC | 2e^-97^ | 86% | 1e^-34^ | 94% | 1 | NW_003763650.1 (NADP-dependent malic enzyme, mitochondrial) | --- |
| **55** | HG531735 | 1-285 | 265-374 | TACACCAACCCCAATCGAACC | 5e^-141^ | 99% | 3e^-36^ | 92% | 1 | NW_003763650.1 (NADP-dependent malic enzyme, mitochondrial) | --- |
| **60** | HG531736 | 1-187 | 180-224 | TGAGATGC | 6e^-74^ | 95% | 8e^-09^ | 91% | 1 | NW_003763650.1 (Not determined) | --- |
| **60** | HG531737 | 1-279 | 262-480 | CCAACCCCAATCGAACCA | 2e^-108^ | 91% | 7e^-90^ | 93% | 2 | NW_003763686.1 (Not determined) | CR1 (Non LTR) |
| **60** | HG531738 | 1-543 | 525-728 | CACCAACCCCAATCGAACC | 2e^-75^ | 96% | 1e^-83^ | 94% | Not determined | NW_003779328.1 (Not determined) | --- |
| **67** | HG531739 | 1-304 | 275-387 | TACACCAACCCCAATCGAACCCTAACCACC | 1e^-117^ | 93% | 2e^-32^ | 89% | 1 | NW_003763650.1 (NADP-dependent malic enzyme, mitochondrial) | --- |
| **67** | HG531740 | 1-167 | 146-258 | ATACACCAACCCCAATCGAACC | 1e^-44^ | 90% | 6e^-37^ | 92% | 1 | NW_003763650.1 (NADP-dependent malic enzyme, mitochondrial) | --- |
| **67** | HG531741 | 1-716 | 697-832 | ATAATGTACGGGGGAGATGC | 0.0 | 85% | 4e^-52^ | 96% | 1 | NW_003763650.1 (Platelet-derived growth fator D) | --- |
| **70** | HG531742 | 1-280 | 272-406 | TGAGATGCA | 9e^-77^ | 82% | 2e^-44^ | 97% | 1 | NW_003763584.1 (Not determined) | --- |
| **70** | HG531743 | 1-333 | 325-431 | TGAGATGCA | 3e^-76^ | 93% | 1e^-45^ | 98% | 1 | NW_003763584.1 (Not determined) | --- |
| **70** | HG531744 | 1-217 | 220-744 |  | 4e^-70^ | 90% | 0.0 | 98% | 5 | NW_003763785.1  (Not determined) | --- |
| **70** | HG531745 | 1-588 | 573-655 | GTACGGGTGAGATGC | 6e^-124^ | 75% | 2e^-23^ | 90% | 1 | NW_003763482.1 (Not determined) | --- |
| **70** | HG531746 | 156-522 | 1-162 | GAAGGCC | 3e^-83^ | 80% | 3e^-20^ | 92% | 1 | NW_003763484.1 (Not determined) | --- |
| **72** | HG531747 | 1-150 | 142-248 | TGAGATGC | 8e^-54^ | 94% | 4e^-45^ | 98% | 1 | NW_003763584.1 (Not determined) | --- |
| **72** | HG531748 | 1-90 | 72-618 | CCAACCCCAATCGAACCAA | 2e^-29^ | 92% | 0.0 | 93% | 5 | NW_003763785.1 (Not determined) | --- |
| **72** | HG531749 | 98-155 | 1-106 | TGCATCTCA | 2e^-15^ | 96% | 4e^-42^ | 95% | 1 | NW_003763584.1 (Not determined) | --- |
| **72** | HG531750 | 1-105 | 88-283 | CCAACCCCAATCGAACCC | 1e^-39^ | 96% | 1e^-91^ | 98% | 9 | NW_003763840.1 (Not determined) | CR1 (Non LTR) |
| **72** | HG531751 | 1-219 /  437-550 | 202-436 /  537-593 | ACTCACTTCTCACTTCCTCCCTTCCC /  TCTCACTTNCTCCC | 3e^-78^ / 2e^-47^ | 96% /  90% | 1e^-12^ /  6e^-09^ | 70% /  93% | 12 /  15 | NW_003763903.1 (Uncharacterized protein LOC416093)/ NW_001471461.2 (cytospin-A) | --- /  --- |
| **72** | HG531752 | 1-80 / 164-379 /  495-564 | 81-170 /  380-501 | CCCTCCC /  CCCTCCC | 8e^-27^ / 1e^-80^ | 97% /  92% | 0.26 /  1e^-05^ | 70% /  71% | Z /  Z | NW_003764324.1 (Not determined) /  NW_003764324.1 (Not determined) | --- /  --- |
| **73** | HG531753 | 1-285 | 263-340 | CCTACACCAACCCCAATCGAACC | 3e^-137^ | 99% | 7e^-25^ | 94% | 20 | NW_003764128.1 (Not determined) | --- |
| **73** | HG531754 | 1-150 | 135-212 | CAACCCCAATCGAACC | 5e^-43^ | 88% | 5e^-24^ | 92% | 20 | NW_003764128.1 (Not determined) | --- |
| **73** | HG531755 | 304-610 | 1-317 | GAACGCCCCTCCCA | 4e^-06^ | 67% | 5e^-157^ | 99% | 20 | NW_003764127.1 (Not determined) | --- |
| **73** | HG531756 | 252-381 | 1-265 | GAAGCCCCCTCCCA | 6e^-47^ | 97% | 2e^-127^ | 98% | 12 | NW_003763892.1 (Not determined) | --- |
| **73** | HG531757 | 1-290 | 285-456 | ATAATG | 3e^-46^ | 96% | 5e^-142^ | 99% | 1 | NW_003763650.1 (Not determined) | --- |
| **73** | HG531758 | 1-160 | 142-373 | GACCCCCCCTCCCAAAACC | 9e^-26^ | 100% | 1e^-67^ | 96% | 1 | NW_001471534.2 ([Pyruvate dehydrogenase [lipoamide]] kinase isozyme 3, mitochondrial) | CR1 (Non LTR) |
| **73** | HG531759 | 142-324 | 1-152 | GACGCCCCCTC | 7e^-44^ | 87% | 9e^-49^ | 87% | 1 | NW_001471534.2 ([Pyruvate dehydrogenase [lipoamide]] kinase isozyme 3, mitochondrial) | CR1 (Non LTR) |
