# Supplementary material for: Inhibition of Autoimmune Chagas-Like Heart Disease by Bone Marrow Transplantation
Source: PLoS Negl Trop Dis. 2014 Dec 18;8(12):e3384. doi: 10.1371/journal.pntd.0003384 (PMC4270743; doi:10.1371/journal.pntd.0003384)
Supplement: S3 Table — Putative gRNAs within kDNA-host DNA chimera sequences in the Gallus gallus genome. (DOCX) [file pntd.0003384.s004.docx]

**Table S3** Putative gRNAs within kDNA-host DNA chimera sequences in the *Gallus gallus* genome.

| **Aves** | **Accession number** | ***Locus*** | **Kiss Database** | | | **Accesion number of pre-RNA-edited gene** | **pre-RNA-edited gene** |
| --- | --- | --- | --- | --- | --- | --- | --- |
|  |  |  | **Hit** | **% Id.** | **Interval seq. lenght** |  |  |
| 1 | HG531391 | Not determined | TO2604042_N10_ND7BSG05M13R_035.ab1 | 53% | 143-205 | [gb\|M94286.1\|TRBKPGEN](http://www.ncbi.nlm.nih.gov/nuccore/gb\|M94286.1\|) | NADH dehydrogenase subunit 7 |
| 2 | HG531395 | NW_001471534.2 (Dystrophin) | Tbrps12ed | 54% | 125-183 | [gb\|M77751.1\|TRBCR6MC](http://www.ncbi.nlm.nih.gov/nuccore/gb\|M77751.1) | CR6 |
| 2 | HG531396 | NW_001471534.2 (Dystrophin) | TO2604042_N10_ND7BSG05M13R_035.ab1 | 52% | 89-151 | [gb\|M94286.1\|TRBKPGEN](http://www.ncbi.nlm.nih.gov/nuccore/gb\|M94286.1\|) | NADH dehydrogenase subunit 7 |
| 2 | HG531398 | Not determined | Tba6ed | 51% | 305-347 | [gb\|M33228.1\|TRBKPEATA](http://www.ncbi.nlm.nih.gov/nuccore/gb\|M33228.1\|) | ATPase 6 |
| 3 | HG531399 | NW_001471534.2 (Dystrophin) | Tbnd3ed | 58% | 148-190 | [gb\|L26251.1\|TRBND3](http://www.ncbi.nlm.nih.gov/nucleotide/468423?report=genbank&log$=nuclalign&blast_rank=1&RID=7JG7303W01R) | NADH dehydrogenase 3: ND3,G5,CR5 |
| 3 | HG531399 | NW_001471534.2 (Dystrophin) | TO151203_E08_B3C04m13r_028.ab1 | 60% | 782-824 | [emb\|V01390.1\|](http://www.ncbi.nlm.nih.gov/nucleotide/14269?report=genbank&log$=nuclalign&blast_rank=1&RID=7JJPJ4VX01R) | 12S rRNA and a 9S rRNA |
| 4 | HG531417 | Not determined | TO2604042_D18_ND7BSB09M13R_077.ab1 | 50% | 497-572 | [gb\|M55645.1\|TRBKPDHAB](http://www.ncbi.nlm.nih.gov/nuccore/gb%7CM55645.1%7C) | NADH dehydrogenase subunit 7 |
| 4 | HG531419 | Not determined | TO151203_F18_B4C09m13r_075.ab1 | 51% | 473-422 | [emb\|X02547.1\|](http://www.ncbi.nlm.nih.gov/nuccore/emb%7CX02547.1%7C) | 12S and 9S ribosomal |
| 5 | HG531426 | NW_001471512.1 (Tetraspanin-12) | Tbnd3ed | 58% | 342-384 | [gb\|L26251.1\|](http://www.ncbi.nlm.nih.gov/nuccore/gb%7CL26251.1%7C) | NADH dehydrogenase 3: ND3,G5,CR5 |
| 6 | HG531432 | Not determined | Tbnd3ed | 48% | 226-270 | [gb\|L26251.1\|](http://www.ncbi.nlm.nih.gov/nuccore/gb%7CL26251.1%7C) | NADH dehydrogenase 3: ND3,G5,CR5 |
| 15 | HG531438 | Not determined | TO270442_ND8PCB05M13R_C09.ab1 | 53% | 122-164 | [gb\|M14820.1\|](http://www.ncbi.nlm.nih.gov/nuccore/gb%7CM14820.1%7C) | cytochrome c oxidase subunit I (COI), and NADH dehydrogenase subunits 4 and 5 |
| 15 | HG531439 | Not determined | TO2604042_N10_ND7BSG05M13R_035.ab1 | 53% | 144-206 | [gb\|M94286.1\|](http://www.ncbi.nlm.nih.gov/nuccore/gb%7CM94286.1%7C) | NADH dehydrogenase subunit 7 |
| 15 | HG531440 | NW_003763785.1 (Tetraspanin-18) | TOCo3001_N16_TbOCo3001aG08.g1_052.ab1 | 48% | 132-193 | [ref\|XM_814182.1\|](http://www.ncbi.nlm.nih.gov/nuccore/ref%7CXM_814182.1%7C) | serine/threonine protein phosphatase |
| 18 | HG531442 | Not determined | Tba6ed | 60% | 146-203 | [gb\|M33228.1\|](http://www.ncbi.nlm.nih.gov/nuccore/gb%7CM33228.1%7C) | ATPase 6 |
| 18 | HG531443 | Not determined | TO270442_ND9BSA08M13R_B16.ab1 | 48% | 155-199 | [ref\|XM_812410.1\|](http://www.ncbi.nlm.nih.gov/nuccore/ref%7CXM_812410.1%7C) | hypothetical protein |
| 18 | HG531444 | Not determined | TOCo3001_K18_TOCo3001F09.g1_069.ab1 | 59% | 129-182 | [ref\|XM_808058.1\|](http://www.ncbi.nlm.nih.gov/nuccore/ref%7CXM_808058.1%7C) | hypothetical protein |
| 23 | HG531467 | NW_001471534.2 (Dystrophin) | Tbnd3ed | 58% | 148-190 | [gb\|L26251.1\|](http://www.ncbi.nlm.nih.gov/nuccore/gb%7CL26251.1%7C) | NADH dehydrogenase 3: ND3,G5,CR5 |
| 23 | HG531469 | NW_001471513.1 (Similar to SH3-domain binding protein 1) | TO161203_A09_B5A05M13R_047.ab1 | 48% | 262-308 | [gb\|S73851.1\|](http://www.ncbi.nlm.nih.gov/nuccore/gb%7CS73851.1%7C) | cytochrome oxidase subunit III transcript |
| 25 | HG531489 | Not determined | TO151203_I23_B1E12m13r_088.ab1 | 47% | 156-226 | [gb\|M14820.1\|](http://www.ncbi.nlm.nih.gov/nuccore/gb%7CM14820.1%7C) | cytochrome c oxidase subunit I (COI), and NADH dehydrogenase subunits 4 and 5 |
| 26 | HG531494 | NW_001471534.2 (Pyruvate dehydrogenase kinase, isozyme 3) | TO161203_E08_B7C04M13R_028.ab1 | 64% | 288-335 | [emb\|X02547.1\|](http://www.ncbi.nlm.nih.gov/nuccore/emb%7CX02547.1%7C) | 12S and 9S ribosomal |
| 26 | HG531495 | NW_001471685.2 (Amyloid beta A4 precursor protein-binding family B member 2) | TO161203_E08_B7C04M13R_028.ab1 | 64% | 372-419 | [emb\|X02547.1\|](http://www.ncbi.nlm.nih.gov/nuccore/emb%7CX02547.1%7C) | 12S and 9S ribosomal |
| 26 | HG531496 | NW_003763668.1 (Eukaryotic translation initiation factor 1b) | TO151203_E13_B1C07m13r_059.ab1 | 59% | 343-292 | [ref\|XM_806453.1\|](http://www.ncbi.nlm.nih.gov/nuccore/ref%7CXM_806453.1%7C) | hypothetical protein |
| 27 | HG531497 | NW_001471554.1 (Similar to spinal cord-derived growth factor-B- SCDGF-B) | TOCo3001_A20_TOCo3001A10.g1_080.ab1 | 55% | 164-219 | [gb\|S73851.1\|](http://www.ncbi.nlm.nih.gov/nuccore/gb%7CS73851.1%7C) | cytochrome oxidase subunit III transcript |
| 27 | HG531498 | NW_001471428.1 (ST8 alpha-N-acetyl-neuraminide alpha-2,8 -sialyltransferase 2) | TOCo3001_A20_TOCo3001A10.g1_080.ab1 | 55% | 163-218 | [gb\|S73851.1\|](http://www.ncbi.nlm.nih.gov/nuccore/gb%7CS73851.1%7C) | cytochrome oxidase subunit III transcript |
| 27 | HG531499 | NW_001471554.1 (Similar to spinal cord-derived growth factor-B- SCDGF-B) | TO161203_A09_B5A05M13R_047.ab1 | 46% | 173-219 | [gb\|S73851.1\|](http://www.ncbi.nlm.nih.gov/nuccore/gb%7CS73851.1%7C) | cytochrome oxidase subunit III transcript |
| 27 | HG531500 | NW_001488823.1 (3-oxoacid CoA transferase 1) | Tba6ed | 69% | 167-125 | [gb\|M33228.1\|](http://www.ncbi.nlm.nih.gov/nuccore/gb%7CM33228.1%7C) | ATPase 6 |
| 27 | HG531501 | NW_001488823.1 (3-oxoacid CoA transferase 1) | Tbnd3ed | 47% | 149-190 | [gb\|L26251.1\|](http://www.ncbi.nlm.nih.gov/nuccore/gb%7CL26251.1%7C) | NADH dehydrogenase 3: ND3,G5,CR5 |
| 27 | HG531502 | NW_001471651.1 (Similar to aminopeptidase) | Tbnd3ed | 58% | 148-190 | [gb\|L26251.1\|](http://www.ncbi.nlm.nih.gov/nuccore/gb%7CL26251.1%7C) | NADH dehydrogenase 3: ND3,G5,CR5 |
| 27 | HG531503 | NW_001488823.1 (3-oxoacid CoA transferase 1) | Tbnd3ed | 58% | 148-190 | [gb\|L26251.1\|](http://www.ncbi.nlm.nih.gov/nuccore/gb%7CL26251.1%7C) | NADH dehydrogenase 3: ND3,G5,CR5 |
| 27 | HG531504 | NW_001471673.1 (Suppressor of Ty 3 homolog) | Tbnd3ed | 55% | 148-190 | [gb\|L26251.1\|](http://www.ncbi.nlm.nih.gov/nuccore/gb%7CL26251.1%7C) | NADH dehydrogenase 3: ND3,G5,CR5 |
| 27 | HG531505 | Not determined | TOCo3001_N16_TbOCo3001aG08.g1_052.ab1 | 46% | 133-194 | [gb\|AY169988.1\|](http://www.ncbi.nlm.nih.gov/nuccore/gb%7CAY169988.1%7C) | _____ |
| 27 | HG531506 | NW_003763785.1 (Tetraspanin-18) | TO161203_A09_B5A05M13R_047.ab1 | 48% | 173-219 | [gb\|S73851.1\|](http://www.ncbi.nlm.nih.gov/nuccore/gb%7CS73851.1%7C) | cytochrome oxidase subunit III transcript |
| 27 | HG531507 | NW_003763785.1 (Tetraspanin-18) | TOCo3001_N16_TbOCo3001aG08.g1_052.ab1 | 46% | 132-193 | [gb\|AY169988.1\|](http://www.ncbi.nlm.nih.gov/nuccore/gb%7CAY169988.1%7C) | _____ |
| 28 | HG531508 | NW_003763584.1 (gamma-aminobutyric acid receptor subunit gamma-3) | TO270442_ND9BSA08M13R_B16.ab1 | 41% | 423-465 | [ref\|XM_812690.1\|](http://www.ncbi.nlm.nih.gov/nuccore/ref%7CXM_812690.1%7C) | hypothetical protein |
| 28 | HG531509 | Not determined | TO2604042_D18_ND7BSB09M13R_077.ab1 | 50% | 835-910 | [gb\|M55645.1\|](http://www.ncbi.nlm.nih.gov/nuccore/gb%7CM55645.1%7C) | NADH dehydrogenase subunit 7 |
| 37 | HG531513 | Not determined | PC_9C05primer.ab1 | 42% | 148-210 | [gb\|S73851.1\|](http://www.ncbi.nlm.nih.gov/nuccore/gb%7CS73851.1%7C) | cytochrome oxidase subunit III transcript |
| 42 | HG531514 | NW_001471534.2 (Dystrophin) | TO171203_L21_B10F11M13R_085.ab1 | 50% | 175-120 | [gb\|M14820.1\|](http://www.ncbi.nlm.nih.gov/nuccore/gb%7CM14820.1%7C) | cytochrome c oxidase subunit I (COI), and NADH dehydrogenase subunits 4 and 5 |
| 45 | HG531518 | NW_003763668.1 (parathyroid hormone-responsive B1) | Tbnd3ed | 58% | 332-374 | [gb\|L26251.1\|](http://www.ncbi.nlm.nih.gov/nuccore/gb%7CL26251.1%7C) | NADH dehydrogenase 3: ND3,G5,CR5 |
| 54 | HG531520 | Not determined | Tba6ed | 69% | 1355-1313 | [gb\|M33228.1\|](http://www.ncbi.nlm.nih.gov/nuccore/gb%7CM33228.1%7C) | ATPase 6 |
| 54 | HG531521 | Not determined | Tba6ed | 69% | 455-413 | [gb\|M33228.1\|](http://www.ncbi.nlm.nih.gov/nuccore/gb%7CM33228.1%7C) | ATPase 6 |
| 55 | HG531522 | NW_001471534.2 (Dystrophin) | Tbnd3ed | 58% | 148-190 | [gb\|L26251.1\|](http://www.ncbi.nlm.nih.gov/nuccore/gb%7CL26251.1%7C) | NADH dehydrogenase 3: ND3,G5,CR5 |
| 57 | HG531532 | Not determined | TO2604042_L04_ND7BSF02M13R_006.ab1 | 46% | 554-596 | [gb\|M55645.1\|](http://www.ncbi.nlm.nih.gov/nuccore/gb%7CM55645.1%7C) | NADH dehydrogenase subunit 7 |
| 58 | HG531535 | Not determined | TO171203_I19_B9E10M13R_072.ab1 | 60% | 310-360 | [emb\|X02547.1\|](http://www.ncbi.nlm.nih.gov/nuccore/emb%7CX02547.1%7C) | 12S and 9S ribosomal |
| 60 | HG531551 | Not determined | Tbnd3ed | 58% | 443-485 | [gb\|L26251.1\|](http://www.ncbi.nlm.nih.gov/nuccore/gb%7CL26251.1%7C) | NADH dehydrogenase 3: ND3,G5,CR5 |
| 60 | HG531552 | Not determined | TO171203_N19_B10G10M13R_068.ab1 | 46% | 561-491 | [ref\|XM_815605.1\|](http://www.ncbi.nlm.nih.gov/nuccore/ref%7CXM_815605.1%7C) | hypothetical protein |
| 60 | HG531553 | NW_003763661.1 (integrin alpha-8 precursor) | Tbnd3ed | 58% | 571-613 | [gb\|L26251.1\|](http://www.ncbi.nlm.nih.gov/nuccore/gb%7CL26251.1%7C) | NADH dehydrogenase 3: ND3,G5,CR5 |
| 61 | HG531557 | NW_001471639.1 (Similar to KIAA0222) | Tbrps12ed | 57% | 126-186 | [gb\|M77751.1\|](http://www.ncbi.nlm.nih.gov/nuccore/gb%7CM77751.1%7C) | ORF1 and ORF2 (CR6) |
| 61 | HG531558 | NW_001471698.1 (UDP-N-acetyl-alpha-D-galactosamine:poly peptide N-acetyl galactosaminyltransferase-like 4) | Tbrps12ed | 59% | 126-186 | [gb\|M77751.1\|](http://www.ncbi.nlm.nih.gov/nuccore/gb%7CM77751.1%7C) | ORF1 and ORF2 (CR6) |
| 62 | HG531559 | Not determined | TOCo3001_N16_TbOCo3001aG08.g1_052.ab1 | 43% | 132-193 | [gb\|AY169988.1\|](http://www.ncbi.nlm.nih.gov/nuccore/gb%7CAY169988.1%7C) | _____ |
| 62 | HG531561 | Not determined | TO270442_ND8BSA12M13R_B23.ab1 | 56% | 244-294 | [gb\|M63820.1\|](http://www.ncbi.nlm.nih.gov/nuccore/gb%7CM63820.1%7C) | NADH dehydrogenase subunit 8 (ND8) |
| 62 | HG531562 | Not determined | TO270442_ND8BSA12M13R_B23.ab1 | 56% | 306-356 | [gb\|M63820.1\|](http://www.ncbi.nlm.nih.gov/nuccore/gb%7CM63820.1%7C) | NADH dehydrogenase subunit 8 (ND8) |
| 62 | HG531563 | Not determined | TO270442_ND8BSA12M13R_B23.ab1 | 56% | 304-354 | [gb\|M63820.1\|](http://www.ncbi.nlm.nih.gov/nuccore/gb%7CM63820.1%7C) | NADH dehydrogenase subunit 8 (ND8) |
| 66 | HG531564 | Not determined | TOCo3001_C12_TOCo3001B06.g1_046.ab1 | 51% | 100-155 | [gb\|S73851.1\|](http://www.ncbi.nlm.nih.gov/nuccore/gb%7CS73851.1%7C) | cytochrome oxidase subunit III transcript |
| 66 | HG531565 | Not determined | TO2604042_N10_ND7BSG05M13R_035.ab1 | 53% | 144-206 | [gb\|M94286.1\|](http://www.ncbi.nlm.nih.gov/nuccore/gb%7CM94286.1%7C) | NADH dehydrogenase subunit 7 |
| 66 | HG531566 | Not determined | TO161203_A09_B5A05M13R_047.ab1 | 48% | 173-219 | [gb\|S73851.1\|](http://www.ncbi.nlm.nih.gov/nuccore/gb%7CS73851.1%7C) | cytochrome oxidase subunit III transcript |
| 66 | HG531566 | Not determined | Tbnd3ed | 58% | 148-190 | [gb\|L26251.1\|](http://www.ncbi.nlm.nih.gov/nuccore/gb%7CL26251.1%7C) | NADH dehydrogenase 3: ND3,G5,CR5 |
| 66 | HG531568 | Not determined | TO270442_ND8BSA12M13R_B23.ab1 | 56% | 164-214 | [gb\|M63820.1\|](http://www.ncbi.nlm.nih.gov/nuccore/gb%7CM63820.1%7C) | NADH dehydrogenase subunit 8 (ND8) |
| 66 | HG531569 | Not determined | TO151203_F04_B4C02m13r_012.ab1 | 62% | 157-118 | [gb\|L22643.1\|](http://www.ncbi.nlm.nih.gov/nuccore/gb%7CL22643.1%7C) | cytochrome oxidase subunit II (COII) |
| 66 | HG531570 | NW_001471639.1 (Similar to KIAA0222) | TO151203_F04_B4C02m13r_012.ab1 | 62% | 157-118 | [gb\|L22643.1\|](http://www.ncbi.nlm.nih.gov/nuccore/gb%7CL22643.1%7C) | cytochrome oxidase subunit II (COII) |
| 67 | HG531575 | NW_003763740.1 (Collagen alpha-1(XXV) chain) | Tbnd3ed | 51% | 131-173 | [gb\|L26251.1\|](http://www.ncbi.nlm.nih.gov/nuccore/gb%7CL26251.1%7C) | NADH dehydrogenase 3: ND3,G5,CR5 |
| 67 | HG531576 | Not determined | Tbnd3ed | 53% | 147-189 | [gb\|L26251.1\|](http://www.ncbi.nlm.nih.gov/nuccore/gb%7CL26251.1%7C) | NADH dehydrogenase 3: ND3,G5,CR5 |
| 68 | HG531577 | Not determined | TO270442_ND8PCB05M13R_C09.ab1 | 53% | 122-164 | [gb\|M14820.1\|](http://www.ncbi.nlm.nih.gov/nuccore/gb%7CM14820.1%7C) | cytochrome c oxidase subunit I (COI), and NADH dehydrogenase subunits 4 and 5 |
| 68 | HG531579 | Not determined | TO2604042_J10_ND7BSE05M13R_039.ab1 | 57% | 144-193 | [gb\|M55645.1\|](http://www.ncbi.nlm.nih.gov/nuccore/gb%7CM55645.1%7C) | NADH dehydrogenase subunit 7 |
| 68 | HG531580 | NW_003763650.1 (NADP-dependent malic enzyme, mitochondrial) | TO151203_F21_B2C11m13r_091.ab1 | 53% | 172-121 | [emb\|X02547.1\|](http://www.ncbi.nlm.nih.gov/nuccore/emb%7CX02547.1%7C) | 12S and 9S ribosomal |
| 68 | HG531581 | Not determined | Tbnd3ed | 47% | 149-190 | [gb\|L26251.1\|](http://www.ncbi.nlm.nih.gov/nuccore/gb%7CL26251.1%7C) | NADH dehydrogenase 3: ND3,G5,CR5 |
| 74 | HG531585 | NW_003763892.1 (Dedicator of cytokinesis protein 3) | Tba6ed | 69% | 242-200 | [gb\|M33228.1\|](http://www.ncbi.nlm.nih.gov/nuccore/gb%7CM33228.1%7C) | ATPase 6 |
| 76 | HG531586 | NW_003764078.1 (Uncharacterized protein LOC100858297) | Tba6ed | 69% | 544-502 | [gb\|M33228.1\|](http://www.ncbi.nlm.nih.gov/nuccore/gb%7CM33228.1%7C) | ATPase 6 |
| 76 | HG531587 | NW_003763854.1 (Immunoglobulin superfamily DCC subclass member 4-like) | Tba6ed | 69% | 351-309 | [gb\|M33228.1\|](http://www.ncbi.nlm.nih.gov/nuccore/gb%7CM33228.1%7C) | ATPase 6 |
| 76 | HG531588 | NW_003763931.1 (Ubiquitin carboxyl-terminal hydrolase 22-A) | Tba6ed | 69% | 555-513 | [gb\|M33228.1\|](http://www.ncbi.nlm.nih.gov/nuccore/gb%7CM33228.1%7C) | ATPase 6 |
| 76 | HG531589 | Not determined | Tba6ed | 69% | 694-652 | [gb\|M33228.1\|](http://www.ncbi.nlm.nih.gov/nuccore/gb%7CM33228.1%7C) | ATPase 6 |
| 76 | HG531590 | Not determined | Tba6ed | 69% | 549-507 | [gb\|M33228.1\|](http://www.ncbi.nlm.nih.gov/nuccore/gb%7CM33228.1%7C) | ATPase 6 |
| 1 | HG531592 | NW_001471534.2 (Dystrophin) | Tbnd3ed | 58% | 148-190 | [gb\|L26251.1\|](http://www.ncbi.nlm.nih.gov/nuccore/gb%7CL26251.1%7C) | NADH dehydrogenase 3: ND3,G5,CR5 |
| 1 | HG531593 | NW_003763785.1 (Transcription Factor SOX-6) | TO2604042_N10_ND7BSG05M13R_035.ab1 | 53% | 601-663 | [gb\|M94286.1\|](http://www.ncbi.nlm.nih.gov/nuccore/gb%7CM94286.1%7C) | NADH dehydrogenase subunit 7 |
| 1 | HG531594 | NW_003763668.1 (Protein PTHB1) | TO2604042_N10_ND7BSG05M13R_035.ab1 | 53% | 328-390 | [gb\|M94286.1\|](http://www.ncbi.nlm.nih.gov/nuccore/gb%7CM94286.1%7C) | NADH dehydrogenase subunit 7 |
| 1 | HG531595 | NW_003763668.1 (Protein PTHB1) | Tbnd9ed | 67% | 430-373 | [gb\|L05586.1\|](http://www.ncbi.nlm.nih.gov/nuccore/gb%7CL05586.1%7C) | IsTaR 1 serodeme NADH dehydrogenase (nd9) |
| 1 | HG531600 | NW_003763650.1 (NADP-dependent malic enzyme, mitochondrial) | Tbnd3ed | 58% | 148-190 | [gb\|L26251.1\|](http://www.ncbi.nlm.nih.gov/nuccore/gb%7CL26251.1%7C) | NADH dehydrogenase 3: ND3,G5,CR5 |
| 2 | HG531602 | Not determined | TO171203_P03_B10H02M13R_002.ab1 | 37% | 106-158 | [gb\|M14820.1\|](http://www.ncbi.nlm.nih.gov/nuccore/gb%7CM14820.1%7C) | cytochrome c oxidase subunit I (COI), and NADH dehydrogenase subunits 4 and 5 |
| 3 | HG531613 | NW_003763650.1 (NADP-dependent malic enzyme, mitochondrial) | Tbnd3ed | 58% | 148-190 | [gb\|L26251.1\|](http://www.ncbi.nlm.nih.gov/nuccore/gb%7CL26251.1%7C) | NADH dehydrogenase 3: ND3,G5,CR5 |
| 5 | HG531619 | NW_001471534.2 (Dystrophin) | Tbnd3ed | 58% | 148-190 | [gb\|L26251.1\|](http://www.ncbi.nlm.nih.gov/nuccore/gb%7CL26251.1%7C) | NADH dehydrogenase 3: ND3,G5,CR5 |
| 11 | HG531625 | Not determined | Tba6ed | 72% | 432-390 | [gb\|M33228.1\|](http://www.ncbi.nlm.nih.gov/nuccore/gb%7CM33228.1%7C) | ATPase 6 |
| 11 | HG531626 | NW_003763812.1 (Similar to atractin 1, isoform 2) | Tba6ed | 72% | 320-278 | [gb\|M33228.1\|](http://www.ncbi.nlm.nih.gov/nuccore/gb%7CM33228.1%7C) | ATPase 6 |
| 11 | HG531627 | Not determined | Tba6ed | 72% | 226-184 | [gb\|M33228.1\|](http://www.ncbi.nlm.nih.gov/nuccore/gb%7CM33228.1%7C) | ATPase 6 |
| 11 | HG531628 | Not determined | Tba6ed | 72% | 206-164 | [gb\|M33228.1\|](http://www.ncbi.nlm.nih.gov/nuccore/gb%7CM33228.1%7C) | ATPase 6 |
| 11 | HG531629 | Not determined | Tba6ed | 72% | 476-434 | [gb\|M33228.1\|](http://www.ncbi.nlm.nih.gov/nuccore/gb%7CM33228.1%7C) | ATPase 6 |
| 14 | HG531643 | Not determined | TO2604042_N10_ND7BSG05M13R_035.ab1 | 53% | 258-320 | [gb\|M94286.1\|](http://www.ncbi.nlm.nih.gov/nuccore/gb%7CM94286.1%7C) | NADH dehydrogenase subunit 7 |
| 14 | HG531644 | Not determined | TO2604042_N10_ND7BSG05M13R_035.ab1 | 53% | 275-337 | [gb\|M94286.1\|](http://www.ncbi.nlm.nih.gov/nuccore/gb%7CM94286.1%7C) | NADH dehydrogenase subunit 7 |
| 14 | HG531645 | Not determined | TO270442_ND8PCB05M13R_C09.ab1 | 53% | 520-562 | [gb\|M14820.1\|](http://www.ncbi.nlm.nih.gov/nuccore/gb%7CM14820.1%7C) | cytochrome c oxidase subunit I (COI), and NADH dehydrogenase subunits 4 and 5 |
| 17 | HG531647 | Not determined | TO171203_P03_B10H02M13R_002.ab1 | 41% | 146-198 | [gb\|M14820.1\|](http://www.ncbi.nlm.nih.gov/nuccore/gb%7CM14820.1%7C) | cytochrome c oxidase subunit I (COI), and NADH dehydrogenase subunits 4 and 5 |
| 18 | HG531653 | Not determined | TO270442_ND9BSA08M13R_B16.ab1 | 51% | 155-199 | [ref\|XM_812410.1\|](http://www.ncbi.nlm.nih.gov/nuccore/ref%7CXM_812410.1%7C) | hypothetical protein |
| 18 | HG531654 | Not determined | TO151203_B10_B4A05m13r_047.ab1 | 48% | 177-252 | [gb\|M33228.1\|](http://www.ncbi.nlm.nih.gov/nuccore/gb%7CM33228.1%7C) | ATPase 6 |
| 18 | HG531655 | Not determined | TO151203_B10_B4A05m13r_047.ab1 | 48% | 402-477 | [gb\|M33228.1\|](http://www.ncbi.nlm.nih.gov/nuccore/gb%7CM33228.1%7C) | ATPase 6 |
| 18 | HG531656 | Not determined | TO151203_B10_B4A05m13r_047.ab1 | 48% | 139-214 | [gb\|M33228.1\|](http://www.ncbi.nlm.nih.gov/nuccore/gb%7CM33228.1%7C) | ATPase 6 |
| 18 | HG531657 | Not determined | Tbrps12ed | 57% | 147-205 | [gb\|M77751.1\|](http://www.ncbi.nlm.nih.gov/nuccore/gb%7CM77751.1%7C) | ORF1 and ORF2 (CR6) |
| 28 | HG531683 | Not determined | Tba6ed | 69% | 268-226 | [gb\|M33228.1\|](http://www.ncbi.nlm.nih.gov/nuccore/gb%7CM33228.1%7C) | ATPase 6 |
| 28 | HG531685 | Not determined | Tba6ed | 69% | 431-389 | [gb\|M33228.1\|](http://www.ncbi.nlm.nih.gov/nuccore/gb%7CM33228.1%7C) | ATPase 6 |
| 28 | HG531686 | Not determined | Tba6ed | 69% | 432-390 | [gb\|M33228.1\|](http://www.ncbi.nlm.nih.gov/nuccore/gb%7CM33228.1%7C) | ATPase 6 |
| 28 | HG531687 | Not determined | Tba6ed | 69% | 229-187 | [gb\|M33228.1\|](http://www.ncbi.nlm.nih.gov/nuccore/gb%7CM33228.1%7C) | ATPase 6 |
| 28 | HG531688 | Not determined | Tba6ed | 69% | 529-487 | [gb\|M33228.1\|](http://www.ncbi.nlm.nih.gov/nuccore/gb%7CM33228.1%7C) | ATPase 6 |
| 28 | HG531689 | Not determined | Tba6ed | 69% | 219-277 | [gb\|M33228.1\|](http://www.ncbi.nlm.nih.gov/nuccore/gb%7CM33228.1%7C) | ATPase 6 |
| 36 | HG531691 | Not determined | Tba6ed | 72% | 293-251 | [gb\|M33228.1\|](http://www.ncbi.nlm.nih.gov/nuccore/gb%7CM33228.1%7C) | ATPase 6 |
| 36 | HG531692 | Not determined | Tba6ed | 69% | 402-360 | [gb\|M33228.1\|](http://www.ncbi.nlm.nih.gov/nuccore/gb%7CM33228.1%7C) | ATPase 6 |
| 36 | HG531693 | Not determined | Tba6ed | 69% | 549-507 | [gb\|M33228.1\|](http://www.ncbi.nlm.nih.gov/nuccore/gb%7CM33228.1%7C) | ATPase 6 |
| 36 | HG531694 | Not determined | Tba6ed | 69% | 566-524 | [gb\|M33228.1\|](http://www.ncbi.nlm.nih.gov/nuccore/gb%7CM33228.1%7C) | ATPase 6 |
| 36 | HG531695 | Not determined | Tba6ed | 67% | 435-393 | [gb\|M33228.1\|](http://www.ncbi.nlm.nih.gov/nuccore/gb%7CM33228.1%7C) | ATPase 6 |
| 36 | HG531697 | Not determined | Tba6ed | 69% | 787-745 | [gb\|M33228.1\|](http://www.ncbi.nlm.nih.gov/nuccore/gb%7CM33228.1%7C) | ATPase 6 |
| 36 | HG531698 | Not determined | Tba6ed | 69% | 571-529 | [gb\|M33228.1\|](http://www.ncbi.nlm.nih.gov/nuccore/gb%7CM33228.1%7C) | ATPase 6 |
| 36 | HG531699 | Not determined | Tba6ed | 69% | 323-281 | [gb\|M33228.1\|](http://www.ncbi.nlm.nih.gov/nuccore/gb%7CM33228.1%7C) | ATPase 6 |
| 36 | HG531700 | Not determined | Tba6ed | 69% | 431-389 | [gb\|M33228.1\|](http://www.ncbi.nlm.nih.gov/nuccore/gb%7CM33228.1%7C) | ATPase 6 |
| 42 | HG531716 | Not determined | TO151203_M02_B3G01m13r_003.ab1 | 67% | 344-281 | [emb\|X02547.1\|](http://www.ncbi.nlm.nih.gov/nuccore/emb%7CX02547.1%7C) | 12S and 9S ribosomal |
| 49 | HG531724 | Not determined | Tbnd3ed | 55% | 148-190 | [gb\|L26251.1\|](http://www.ncbi.nlm.nih.gov/nuccore/gb%7CL26251.1%7C) | NADH dehydrogenase 3: ND3,G5,CR5 |
| 49 | HG531725 | Not determined | TO151203_E13_B1C07m13r_059.ab1 | 56% | 717-661 | [ref\|XM_806453.1\|](http://www.ncbi.nlm.nih.gov/nuccore/ref%7CXM_806453.1%7C) | hypothetical protein |
| 55 | HG531735 | Not determined | Tbnd3ed | 58% | 148-190 | [gb\|L26251.1\|](http://www.ncbi.nlm.nih.gov/nuccore/gb%7CL26251.1%7C) | NADH dehydrogenase 3: ND3,G5,CR5 |
| 67 | HG531739 | Not determined | TO0505041_D17_A6BSB09M13F_077.ab1 | 54% | 165-115 | gb\|AY552588.1\| | ____ |

* kDNA hypervariable region fragment. ** Truncated/recombined kDNA. Id, Identity. Seq, Sequence
