# Supplementary material for: Inhibition of Autoimmune Chagas-Like Heart Disease by Bone Marrow Transplantation
Source: PLoS Negl Trop Dis. 2014 Dec 18;8(12):e3384. doi: 10.1371/journal.pntd.0003384 (PMC4270743; doi:10.1371/journal.pntd.0003384)
Supplement: S4 Table — ORF-translated chimera protein sequences from the kDNA+ Gallus gallus somatic cell genome. (DOCX) [file pntd.0003384.s005.docx]

**Table S4.** ORFs translated chimera protein sequences from kDNA+ *Gallus gallus* somatic cells genome.

| **Ave** | **Accession #** | **ORFs translated putative proteins** | **Locus** | **Blastp** | **Score** | ***E-value*** | **Identity** |
| --- | --- | --- | --- | --- | --- | --- | --- |
| **01** | HG531392 | MGAVHRRVSGLRSTFGVEIHASPPYIIIQDLWDTYKV | gi\|242381001\|emb\|  CAR63130.1\| | Hypothetical protein [Homo sapiens] | 113 | 1,09E^-06^ | 24/27 (88.89%) |
| **01** | HG531393 | 1)MADLRGLFSGPPLPKPNFVIFRYHILRAITPYTTTIYHTSPTTNAIILIR | -- | No significant similarity | -- | -- | -- |
|  |  | 2)MWYLKITKFGFGRGGPENNPLKSAIHRSDRTFNVNYEV | -- | No significant similarity | -- | -- | -- |
| **03** | HG531399 | 1)MAENGGCLREARFDWGWCMCIRACYWSIQRFNFFLVWFWEGSSPLLFNFSIKQVCHCDQYYFVRNAVTKCFVSTVKLL | gi\|242380969\|emb\|CAR63115.1\| | Hypothetical protein [Homo sapiens] | 184 | 1,36E^-16^ | 34/36 (94.44%) |
|  |  | 2)MCGWVPKSIGSQFASHPYIIWPKMGVVCGRRGSIGVGVCVSEPVIGLFRDSTSSWFGFGRVLVRSCSILVLSKFAIVTNTTLSEML | gi\|242381063\|emb\|CAR63156.1\| | Hypothetical protein [Homo sapiens] | 80 | 0,145982 | 15/22 (68.18%) |
|  |  | 3)MANLLNTKIEQERTRTLPKPNQEEVESLNRPITGSDTHTPTPIEPRLPQTTPIFGHIMYG | gi\|242380916\|emb\|CAR63092.1\| | Hypothetical protein [Homo sapiens] | 135 | 1,78E^-09^ | 27/29 (93.1%) |
|  |  | 4)MLAPTLHHPK?PCVVGYQKALAPSLHLTRTLYGRKWGLFAGGEVRLGLVYVYQSLLLVYSEIQLLPGLVLGGF | -- | No significant similarity | -- | -- | -- |
| **03** | HG531400 | 1)MYGGDANWEPMLFGTQPHMDSLGDEEWEPTFWVPNHTWDHWVTQSGSQCFLYTNPNGTKTKTRKPNQNEVHVYTSKNKQAKAIFFSHNNFTVLTKHLVTAFLTK | gi\|390438233\|ref\|ZP_10226720.1\| | hypothetical protein MICAI_ 1440007 [Microcystis sp. T1-4] | 72 | 4,24719 | 18/51 (35.29%) |
|  |  | 2)MQTGSQCFLVPNHTWILWVMKSGSQHFGYPTTPGITG | -- | No significant similarity | -- | -- | -- |
| **03** | HG531401 | MAVGAGGADPDRLILFSSVYLFLPPPLRFNPVSWEIPVEIYSLLKYFFSLALRMPSPVRLGLV | -- | No significant similarity | -- | -- | -- |
| **03** | HG531402 | 1)MGLYDCVCVMV?EYEVWKFVKYWFWEGGSTTSLGNLFQCLTTLTVQNFFLISSLNLP | -- | No significant similarity | -- | -- | -- |
|  |  | 2)MEIREILVLGGGVHNLSGQPVPVSHHPHSTEFLPNI | -- | No significant similarity | -- | -- | -- |
| **03** | HG531403 | MHLTRTLWFWEGGSHEPCSRAICWVLLGASSFQRLFWEL | gi\|242380892\|emb\|CAR63085.1\| | Hypothetical protein [Homo sapiens] | 68 | 2,84906 | 13/14 (92.86%) |
| **03** | HG531405 | 1)MGLVSTFGVEIH?SHPYIMVLGGAVKFGPENSCISPVHYFI | -- | No significant similarity | -- | -- | -- |
|  |  | 2)M?LTRTLWFWEGRSNLGRKIHASPPYIILYKYSVYKSPRARRSFSVSFNYSSL?YSVQT | -- | No significant similarity | -- | -- | -- |
| **03** | HG531406 | 1)MQLEAIPSSPITSYTSKEADRPSQNHNVRVRCMNFPAQI | -- | No significant similarity | -- | -- | -- |
|  |  | 2)MVLGGAVKFGPENSCISPVHYGFGRGGRPLCLCN | gi\|242380993\|emb\|CAR63124.1\| | Hypothetical protein [Homo sapiens] | 138 | 8,36E^-10^ | 26/27 (96.3%) |
| **04** | HG531407 | 1)MKEFLYSPTGKFYGLNNAWPFQVLKNFEFSFNEVNAVIMYGWDA | -- | No significant similarity | -- | -- | -- |
|  |  | 2)MGAVDRRVSGLRSTFGVEIHASHPYIITALTSLKENSKFLST | gi\|242381001\|emb\|CAR63130.1\| | hypothetical protein [Homo sapiens] | 136 | 7,21E^-10^ | 26/27 (96.3%) |
| **04** | HG531413 | MKEFLYSPTGKFYGLNNAWPFQVLKNFEFSFNEVNAVIMYG | -- | No significant similarity | -- | -- | -- |
| **04** | HG531415 | 1)MLIVQRTGCTDADGSQSLYSYEGKLHCSRNSGVFSGLLQIAALLPDKNFRLSQLNMHLHLCNILFLLNAPPKTKPGRG | gi\|15139869\|emb\|CAC50076.1\| | NADH dehydrogenase subunit 2 [Isoetes durieui] | 76 | 3,42981 | 20/47 (42.55%) |
|  |  | 2)MCIRACYWSIQRFNLFLVWFWEGRSEEIIYYKDADAYLAVIT | gi\|242380970\|emb\|CAR63116.1\| | Hypothetical protein [Homo sapiens] | 94 | 0,0008309 | 17/18 (94.44%) |
| **04** | HG531416 | 1)MQFPAAFGKSIRRHAVQNFCVNLYPGEPPSQNHNVRVRCMNFPAQV | -- | No significant similarity | -- | -- | -- |
|  |  | 2)MKRRVRLGLVCVYQSLLLVYSEIQLLPGLVLGGAFRFMAEKSCISPVHYGFGRGVQVWAGKFMHLTRTLWFWEGRSSLGRKIHASHPYIMVLGGAFKLGPENSCISPVHYGFGRGVHLDKG | gi\|242380891\|emb\|CAR63084.1\| | Hypothetical protein [Homo sapiens] | 239 | 1,84E^-23^ | 45/55 (81.82%) |
|  |  | 3)MHLTRTLWFWEGRSSLGRKIHASHPYIMVLGGAFKFGPENSCISPVHYGFGRGLQTWAGKFMHLTRTLWFWEGGSPG | gi\|242380993\|emb\|CAR63124.1\| | Hypothetical protein [Homo sapiens] | 198 | 7,16E^-18^ | 35/39 (89.74%) |
|  |  | 4)MVLGGAFKFGPENSCISPVHYGFGRGVQVWAGKFMHLTRTLWFWEGPSNLGRKIHASHPYIMVLGGGFTWIKVNAEVLYCVASY | gi\|242380947\|emb\|CAR63104.2\| | Hypothetical protein [Homo sapiens] | 239 | 1,68E^-24^ | 44/48 (91.67%) |
| **04** | HG531417 | MVLGGAFKFGPENSCISPVHYGFGRGVQVWAGKFMHLTRTLWFWEGRSSLGRKIHASHPYIMVLGGAVILCGESVIF | gi\|242380947\|emb\|CAR63104.2\| | Hypothetical protein [Homo sapiens] | 233 | 1,33E^-23^ | 43/48 (89.58%) |
| **04** | HG531418 | 1)MCIRACYWSIQRFNLFLVWFWEGRSNLGRKIHASHPYIMVLGGAFKFGPENSCISPVHYGFGRGVQTWAGKSMHLTRTLWFWEGRSSLGRKIHASPPYIMVLGREEVRIMLANHINASKAYCE | gi\|242380947\|emb\|CAR63104.2\| | Hypothetical protein [Homo sapiens] | 246 | 6,27E^-25^ | 47/54 (87.04%) |
|  |  | 2)MHLTRTLWFWEGRSSLGRKIHASPPYIMVLGGAFKLGPENPCISPVHYGFGRGVQVWAGKFMHLPRTLWFWEGRK | gi\|242380947\|emb\|CAR63104.2\| | Hypothetical protein [Homo sapiens] | 254 | 7,64E^-27^ | 47/52 (90.38%) |
|  |  | 3)MHLPRTLWFWEGRSNLGRKIHASHPYIMVLGGAFKFGPENSCISPVHYGFGKGGSENNVS | gi\|242380993\|emb\|CAR63124.1\| | Hypothetical protein [Homo sapiens] | 198 | 3,39E^-18^ | 35/40 (87.5%) |
| **04** | HG531419 | MHKQLLTNPQSNSQLTPQAAEERDDPPSQNQISAIS | gi\|383810585\|ref\|ZP_09966079.1\| | Hypothetical protein HMPREF9969_ 1202 [Prevotella sp. oral taxon 306 str. F0472] | 70 | 4,61072 | 11/29 (37.93%) |
| **04** | HG531420 | 1)MHKQLLTNPQSNSQLTPQAAEERDERPSQNQYFTNFHTSYSVTITQTQSYNPIDQIYKL | gi\|198278233\|dbj\|BAG70803.1\| | PreS/S protein [Hepatitis B virus] | 72 | 8,01042 | 16/47 (34.04%) |
|  |  | 2)MGLYDCVCVMVTEYEVWKFVKYWFWEGRSSLSSAA | gi\|242380904\|emb\|CAR63089.1\| | Hypothetical protein [Homo sapiens] | 101 | 5,44E^-05^ | 19/20 (95%) |
| **04** | HG531421 | MCIGACYWSIQRFNLFLVWFWEGRSGCLVLPFQA | gi\|242380949\|emb\|CAR63105.1\| | Hypothetical protein [Homo sapiens] | 83 | 0,0228223 | 16/18 (88.89%) |
| **05** | HG531422 | MVLGGGFRLDIRNNFCPERVVRHCNRLPREVAESLSL | gi\|118086255\|ref\|XP_001236146.1\| | PREDICTED: uncharacterized protein LOC776634 [Gallus gallus] | 99 | 0,00029696 | 22/33 (66.67%) |
| **05** | HG531424 | 1)MAEKSCISLVHYMPENGVCLREVVVRLGLVYVYQSLLLVYSEIQLLPGLVLGGAFRKSQKAEIVFGCF | gi\|242380982\|emb\|CAR63119.1\| | Hypothetical protein [Homo sapiens] | 90 | 0,00723847 | 18/21 (85.71%) |
|  |  | 2)MHLTRTLYARKWGVFTGGGGSIGVGVCVSEPVIGLFRDSTSSWFGFGRGVQEESKSRNCIWMLLKTERLFASVAL | gi\|7959748\|gb\|AAF71046.1\|AF116721_23 | PRO1094 [Homo sapiens] | 83 | 0,0815274 | 17/20 (85%) |
| **05** | HG531425 | 1)MVLGGAFKFGPENSCISPVHYGFGRGVQVWAGKFMHLTRTLWFWEGRSSLGRKIHASHPYIMVLGGAFIC | gi\|242380947\|emb\|CAR63104.2\| | Hypothetical protein [Homo sapiens] | 231 | 1,54E^-23^ | 43/48 (89.58%) |
|  |  | 2)MHLTRTLWFWEGRSSLGRKIHASHPYIMVLGGAFKFGPENSCISPVHYGFGRGVHLLRGRIWGV | gi\|242380993\|emb\|CAR63124.1\| | Hypothetical protein [Homo sapiens] | 195 | 1,05E^-17^ | 35/39 (89.74%) |
|  |  | 3)MVLGGAFKFGPENSCISPVHYGFGRGVQVWAGKFMHLTRTLWFWEGRSFVERADLGSVADLI | gi\|242380993\|emb\|CAR63124.1\| | Hypothetical protein [Homo sapiens] | 144 | 3,05E^-10^ | 26/26 (100%) |
| **05** | HG531426 | MGLYDCVCVMVTEYEVWKFVKYWFWEGRSKICTKRETLFFNINPMSYREKKRDGNIVSPEEIMV | gi\|242380904\|emb\|CAR63089.1\| | Hypothetical protein [Homo sapiens] | 104 | 6,37E^-05^ | 21/30 (70%) |
| **05** | HG531427 | 1)MHLTRTLWFWE?GSEIWFWEGRSS??RKIHASHPYIMVLGGAFKFGPENSC?SPVHYGFGRGVQVWAGKFMHLTRTLWFWEGRSSLGRKIHASHPYIINPYQMMR | -- | No significant similarity | -- | -- | -- |
|  |  | 2)MVLG?GVRDMVLGGAFK??PENSCISPVHYGFGRGVQVWAGKFMH?T?TLWFWEGRSSLGRKIHASHPYIMVLGGAFKFGPENSCISPVHYQSLSDDEIK?EISPSSSCTISKKENKYF?HFVMQTDMNSKSRGREKKS | -- | No significant similarity | -- | -- | -- |
|  |  | 3)MHLTRTLWFWEGRSSLGRKIHA?H?YIMVLGGAFKFGPENSCISPVHYGFGRGVQVWAGKFMHLTRTLSIPIR | -- | No significant similarity | -- | -- | -- |
| **05** | HG531428 | 1)MCIRACYWSIQRFNFFLVWFWEGRSSLGRKIHASHPYIMVLGGVFKFGPENSCISPVHYGFGRGVQVWAGKFMHLTRTLWFWEGRK | gi\|242380947\|emb\|CAR63104.2\| | Hypothetical protein [Homo sapiens] | 247 | 1,14E^-25^ | 46/52 (88.46%) |
|  |  | 2)MHLTRTLWFWEGCSSLGRKIHASHPYIMVLGGAFKFGPENSCISPVHYGFGKGGSER | gi\|242380993\|emb\|CAR63124.1\| | Hypothetical protein [Homo sapiens] | 198 | 2,99E^-18^ | 35/40 (87.5%) |
|  |  | 3)MHLTRTLWFWEGRSSLGRKIHASHPYIMVLGREEVRGDLCTDWLRLLWVLWDGEGV | gi\|242380992\|emb\|CAR63123.1\| | Hypothetical protein [Homo sapiens] | 136 | 9,30E^-10^ | 29/31 (93.55%) |
| **05** | HG531429 | 1)MCIRAC??SIQRFNFFLVWFWEGRSNLWPKIHASHPYIICPKMGCVYGRWWFDWGWCMCIRACYWSIQRFNFFLVWFWEGRSNLWPKNHASHPYIMVLGGAVTL | -- | No significant similarity | -- | -- | -- |
|  |  | 2)MAENSCISPVHYMPENGVCLREVVVRLGLVYVYQSLLLVYSEIQLLPGLVLGGAFKFMAEKSCISPVHYGFGRGRHFVRLCFSGALVETKAALCFYRKATSDCRSQQVSK | gi\|242380982\|emb\|CAR63119.1\| | Hypothetical protein [Homo sapiens] | 129 | 1,06E^-07^ | 26/29 (89.66%) |
| **05** | HG531430 | MHYSFQCNSLSENFFPNFQAEPPSQNHNVRVRCMNFPAQI | -- | No significant similarity | -- | -- | -- |
| **05** | HG531431 | 1)MHLTRTLWFWEGRSSLGRKIHASPPYIMVLGGGVSLFTVCSNAQQHTPTHEGVLS | gi\|242380992\|emb\|CAR63123.1\| | Hypothetical protein [Homo sapiens] | 146 | 2,70E^-11^ | 31/34 (91.18%) |
|  |  | 2)MVLGGAFKFGPENSCISPVHYGFGRGGQFIYCVQ | gi\|242380993\|emb\|CAR63124.1\| | Hypothetical protein [Homo sapiens] | 153 | 5,99E^-12^ | 27/27 (100%) |
|  |  | 3)MHLPRTLWFWEGGSVYLLCAVMPSSTPRPMKVCCHEESAQWSFS | gi\|242380947\|emb\|CAR63104.2\| | Hypothetical protein [Homo sapiens] | 70 | 1,57854 | 12/12 (100%) |
| **06** | HG531432 | MTENSCISPVHYVPLMPTKTQQPRV?LTWAVSC | -- | No significant similarity | -- | -- | -- |
| **06** | HG531433 | MDSVLASDLFC?KVSAFLQNGHLVSGIFIKG?RLLTWTAPPKTANSQNFIPLILATTPTTTHNTPIPIPYHYTFYNILIINIHTLLTTFTIQTFHLYQCTHNTF?YYHTIT | -- | No significant similarity | -- | -- | -- |
| **07** | HG531436 | 1)MKEFLYSPTGK?YGLNNAWPFQVLKNFEFSFNEVN?VIMYG | -- | No significant similarity | -- | -- | -- |
|  |  | 2)MVLGGAVKFGPENSCISPVHYGFGRGR?IWAGKFMHLTRTLSQH | -- | No significant similarity | -- | -- | -- |
|  |  | 3)MHLTRTLWFWEGASNLGRKIHASHPYIIT?LTSLKENSKFLST | -- | No significant similarity | -- | -- | -- |
|  |  | 4)MVLGGGV?FGPENSCISPVHYH?INLIERKFKVFEHLKWPSIIQTI?FTRRRI | -- | No significant similarity | -- | -- | -- |
| **15** | HG531439 | MGSPSLKVLQKCGDVTLREWSVGMVWHGGFDWGWCNIDRGGFMDLIL | gi\|358253606\|dbj\|GAA53490.1\| | Dipeptidyl peptidase 4 [Clonorchis sinensis] | 78 | 1,40468 | 15/37 (40.54%) |
| **18** | HG531442 | MGSPSLKVLQKCGDVTLREWSVGMVWHGGFDWGWCNIDRGCYDNLFLWFRECGYGMYRVE | gi\|358253606\|dbj\|GAA53490.1\| | Dipeptidyl peptidase 4 [Clonorchis sinensis] | 84 | 0,268587 | 20/50 (40%) |
| **18** | HG531443 | MGSPSLKVLQKCGDVTLREWSVGMVWHGGFDWGWCNIVLVW | gi\|358253606\|dbj\|GAA53490.1\| | Dipeptidyl peptidase 4 [Clonorchis sinensis] | 71 | 8,35065 | 14/35 (40%) |
| **18** | HG531444 | MGSPSLKVLQKCGDVTLRVWSVGMVWHGEFDWGWCNIGAEAECL | -- | No significant similarity | -- | -- | -- |
| **18** | HG531446 | MVRHRNRLPIEVVDAPSQNQSSRILNTSFYPPLPTTPTNIL | -- | No significant similarity | -- | -- | -- |
| **20** | HG531449 | 1)MCIRACYWSIQRFNFFLVWFWEGRLNLWPKNHASHPYIIWPKMGVVYGRRGSIGVGVCVSEPAIGLFRDSTSSWFGFGRGVHLSVAQFTN | gi\|242380949\|emb\|CAR63105.1\| | Hypothetical protein [Homo sapiens] | 99 | 0,0005473 | 18/18 (100%) |
|  |  | 2)MAEKSCISPVHYMAENGGCLREARFDWGWCMCIRACYWSIQRFNFFLVWFWEGRSFVSGTIH | gi\|242380969\|emb\|CAR63115.1\| | Hypothetical protein [Homo sapiens] | 184 | 9,34E^-17^ | 34/36 (94.44%) |
| **20** | HG531450 | 1)MCIRACYWSIQRFNFFLVWFWEGRLNLWPKNHASHPYIICPKMGVVYGRRGSIGVGVCVSEPVIGLFRDSTSSWFGFGRGVPFRF | gi\|242380949\|emb\|CAR63105.1\| | Hypothetical protein [Homo sapiens] | 99 | 0,00062284 | 18/18 (100%) |
|  |  | 2)MAEKSCISPVHYMPENGGCLREARFDWGWCMCIRACYWSIQRFNFFLVWFWEGRSL | gi\|242380969\|emb\|CAR63115.1\| | Hypothetical protein [Homo sapiens] | 179 | 3,95E^-16^ | 33/36 (91.67%) |
| **20** | HG531451 | MGVAYGRWWFDWGWCMCIRACYWSIQRFNFFLVWFWEGPSNLWPKNHASPPYVICPKMGCVYGRWWFDWGWCMCIRACYWSIQRFNFFLVWFWEGASTARQSFRSF | gi\|242380969\|emb\|CAR63115.1\| | Hypothetical protein [Homo sapiens] | 123 | 2,30E^-07^ | 23/24 (95.83%) |
| **22** | HG531456 | 1)MDGALSNPLYREVSLPIAGRLEIDNFEGPSQNHNVRVRCMNFPAQI | -- | No significant similarity | -- | -- | -- |
|  |  | 2)MCIRACYWSIQRFNFFLVWFWEGRSNLGRKIHASHPYIMVLGGAFKFGPENSCISPVHYGFGRGLQNYLFPTSLL | gi\|242380993\|emb\|CAR63124.1\| | Hypothetical protein [Homo sapiens] | 196 | 1,30E^-17^ | 35/39 (89.74%) |
|  |  | 3)MHLTRTLWFWEGRSNLGRKIHASHPYIMVLGGAFKIIYFQPPCYRQGHLPVERVAQSPIHPGLEHLPEGGIHNLTGQPVPVSHHPHSKAFFPNI | gi\|242380992\|emb\|CAR63123.1\| | Hypothetical protein [Homo sapiens] | 144 | 1,76E^-10^ | 29/33 (87.88%) |
| **22** | HG531457 | 1)MYQSLLLVYSEIRLLSGLVLGGGFKLGPEKSCISPVHYGFGRGVQIWAGKFMHLTRTLFIYAKK | gi\|242380982\|emb\|CAR63119.1\| | Hypothetical protein [Homo sapiens] | 161 | 7,83E^-13^ | 32/38 (84.21%) |
|  |  | 2)MVLGGAFKFGPENSCISPVHYSFMQRSDQRLYQL | gi\|242380993\|emb\|CAR63124.1\| | Hypothetical protein [Homo sapiens] | 125 | 4,75E^-08^ | 22/23 (95.65%) |
| **22** | HG531458 | MHLTRTLWFWEGRSNLGRKIHASHLYIMVLGGAFKFGPENSCISPVHYGFGRGVQVGY | gi\|242380993\|emb\|CAR63124.1\| | Hypothetical protein [Homo sapiens] | 188 | 1,21E^-16^ | 34/39 (87.18%) |
| **22** | HG531459 | 1)MCIRACYWSIQRFNFFLVWFWEGPSNLWPKNHASPPYIMVLGGAFKFGPENSCISPVHYGFGRGVHVKLIWNTK | gi\|242380993\|emb\|CAR63124.1\| | Hypothetical protein [Homo sapiens] | 172 | 3,71E^-14^ | 32/33 (96.97%) |
|  |  | 2)MAEKSCISPVHYGFGRGVQIWAGKFMHLTRTLWFWEGRSCEANMEYQMNASKSRVLQSCICQCGDGNLPFHLSLMP | gi\|242380947\|emb\|CAR63104.2\| | Hypothetical protein [Homo sapiens] | 180 | 8,75E^-16^ | 33/37 (89.19%) |
|  |  | 3)MHLPRTLWFWEGRSNLGRKIHASHPYIMVLGGAFM | gi\|242380891\|emb\|CAR63084.1\| | Hypothetical protein [Homo sapiens] | 155 | 1,96E^-12^ | 29/34 (85.29%) |
| **22** | HG531460 | MAEKSCISPVHYMPENGVCLREVVVRLGLVYVYQSLLLVYSEIQLLPGLVLGGAFKFMAEKSCISPVHYMPENGVCLREVVVRLGLVYVYQSLLLVYSEIQLLPGLVLGGGVILCGESVIF | gi\|242380982\|emb\|CAR63119.1\| | Hypothetical protein [Homo sapiens] | 101 | 0,00070086 | 19/21 (90.48%) |
| **22** | HG531462 | 1)MCIRACYWSIQRFNFFLVWFWEGPSNLWPKNHASHPYIICPKMGCVYGRWWFDWGWCMCIRACYWSIQRFNFFLVWFWEGRK | gi\|242380969\|emb\|CAR63115.1\| | Hypothetical protein [Homo sapiens] | 123 | 1,43E^-07^ | 23/24 (95.83%) |
|  |  | 2)MAEKSCISPVHYMPENGVCLREVVVRLGLVYVYQSLLLVYSEIQLLPGLVLGREEVRGDLRADWLRLLWVLWDGIRV | gi\|242380982\|emb\|CAR63119.1\| | Hypothetical protein [Homo sapiens] | 101 | 0,00036747 | 19/21 (90.48%) |
|  |  | 3)MHLTRTLYARKWGVFTGGGGSIGVGVCVSEPVIGLFRDSTSSWFGFGKGGSER | gi\|7959748\|gb\|AAF71046.1\|AF116721_23 | PRO1094 [Homo sapiens] | 80 | 0,10242 | 17/25 (68%) |
| **22** | HG531464 | MCIRACYWSIQRFNFFLVWFWEGRSNLGRKIHASHLYIMVLGGAFKFGPENSCISPVHYSFNMYEF | gi\|242380993\|emb\|CAR63124.1\| | Hypothetical protein [Homo sapiens] | 167 | 1,43E^-13^ | 30/37 (81.08%) |
| **22** | HG531465 | 1)MCIRACYWSIQRFNFFLVWFWEGRSNLWPKNHASHPYIICPKMGCVYGRRWFDWGWCMCIRACYWSIQRFNFFLVWFWEGGSGWILGKTSQEW | gi\|242380969\|emb\|CAR63115.1\| | Hypothetical protein [Homo sapiens] | 87 | 0,0181568 | 17/18 (94.44%) |
|  |  | 2)MHLTRTLYARKWGVFTGGGGSIGVGVCVSEPVIGLFRDSTSSWFGFGRGVQVGY | gi\|7959748\|gb\|AAF71046.1\|AF116721_23 | PRO1094 [Homo sapiens] | 83 | 0,0475236 | 17/20 (85%) |
| **23** | HG531469 | MLGIKEAPSQNHNFSIFRTQNLITNVLSTTKHQPNVTPTTPTYTNFIHIIIQTINYNN | -- | No significant similarity | -- | -- | -- |
| **23** | HG531471 | MQEK?PTPPPKTTNSQNFIPLILATTLTTTHNTPIPIPHHYTFYNILIINIHTLLTTFTIQTSHLYQ?TH?TFV?YHTIT | -- | No significant similarity | -- | -- | -- |
| **24** | HG531474 | MVLGGAFKFGPE?HASHPYIMVLGGAVSLEKKRPRGDLIALYYNCLKGGCNEVEVSLFSHVTSDRTRWNDLKLHQGRFRLDIRKNFSKRVVRLWNELPTEVVESPFL | -- | No significant similarity | -- | -- | -- |
| **24** | HG531475 | MVLGGAFKFGPENSCISPVHYGFGKGVQVWAGKFMHLTRTLWFWGGRSSLGRKIHASHPYIMVLGGAFKFGPENSCISPVHYGFGRGLQVGY | gi\|242380947\|emb\|CAR63104.2\| | Hypothetical protein [Homo sapiens] | 219 | 2,43E^-21^ | 41/48 (85.42%) |
| **24** | HG531476 | 1)MVLGGAVKFGPENSCISPVHYGFGRGVQVWAGKFMHLTRTLWFWEGRSLDSWTLWSD | gi\|242380993\|emb\|CAR63124.1\| | Hypothetical protein [Homo sapiens] | 138 | 2,01E^-09^ | 25/26 (96.15%) |
|  |  | 2)MHLTRTLWFWEGRSSLGRKIHASHPYIMVLGGAVTRLMDTLVRLKFWLL | gi\|242380992\|emb\|CAR63123.1\| | Hypothetical protein [Homo sapiens] | 151 | 4,71E^-12^ | 32/36 (88.89%) |
| **24** | HG531477 | 1)MCDSLILCLYYFILHDGPSQNHNVRVRYMNFPAQI | -- | No significant similarity | -- | -- | -- |
|  |  | 2)MHLTRTLWFWEGRSSLGRKIHASPPYIMVLGGAVKFGPENSCISPVHYGFGRGRHGV | gi\|242380993\|emb\|CAR63124.1\| | Hypothetical protein [Homo sapiens] | 177 | 4,51E^-15^ | 33/39 (84.62%) |
|  |  | 3)MVLGGAFKFGPENSCISPVHYGFGRGGQIWAGKFMYLTRTLWFWEGPSWSMK | gi\|242380993\|emb\|CAR63124.1\| | Hypothetical protein [Homo sapiens] | 147 | 6,69E^-11^ | 27/27 (100%) |
|  |  | 4)MHLPRTLWFWEGRSNLGRKIHVSHPYIMVLGGAVMEYEIVKAQN | gi\|242380891\|emb\|CAR63084.1\| | Hypothetical protein [Homo sapiens] | 149 | 2,83E^-11^ | 27/33 (81.82%) |
| **24** | HG531478 | 1)MHLTRTLWFWEGRSSLGRKIHASHPYIMVLGGAFKFGPENSCISPVHYGFGRGLQGA | gi\|242380993\|emb\|CAR63124.1\| | Hypothetical protein [Homo sapiens] | 196 | 5,66E^-18^ | 35/39 (89.74%) |
|  |  | 2)MVLGGAFKFGPENSCISPVHYGFGRGVQVWAGKFMHLTRTLWFWEGASGCLVLPFQA | gi\|242380993\|emb\|CAR63124.1\| | Hypothetical protein [Homo sapiens] | 142 | 4,32E^-10^ | 26/26 (100%) |
|  |  | 3)MHLTRTLWFWEGRSSLGRKIHASHPYIMVLGGGFRVLSSTFPSINKTYEEGKIWSTPNCTTASLLISFFLRKKFPFELAIFSYHSPVHLNLPCTAY | gi\|242380992\|emb\|CAR63123.1\| | Hypothetical protein [Homo sapiens] | 152 | 1,27E^-11^ | 31/33 (93.94%) |
| **24** | HG531479 | 1)MHKQLLTNPQSNSQLTPQAAEERDEPPSQNHNVRVRCMNFPAQT | gi\|381211712\|ref\|ZP_09918783.1\| | Hypothetical protein LGrbi_17433 [Lentibacillus sp. Grbi] | 67 | 6,59154 | 15/31 (48.39%) |
|  |  | 2)MHLTRTLWFWEGRSSLGRKIHASHPYVMVLGGGFISLFCCLRG | gi\|242380992\|emb\|CAR63123.1\| | Hypothetical protein [Homo sapiens] | 148 | 1,09E^-11^ | 30/33 (90.91%) |
|  |  | 3)MVLGGAFKFGPENSCISPVRYGFGRGVHLSLLLLEGLVGSLIGGW | gi\|242380993\|emb\|CAR63124.1\| | Hypothetical protein [Homo sapiens] | 117 | 8,49E^-07^ | 22/23 (95.65%) |
| **24** | HG531480 | MHLTRTLWFWEGRSSLGRKIHASPPYIMVLGGAVILCGESVIF | gi\|242380992\|emb\|CAR63123.1\| | Hypothetical protein [Homo sapiens] | 140 | 1,37E^-10^ | 30/35 (85.71%) |
| **24** | HG531481 | 1)MHLTRTLWFWEGRSSLGRKIHASHPYIMVLGGAFKFGPENSCISPVHYGFGRGGHFVRLCFSGALVETKAALCFYRKATSDCRSQQVSK | gi\|242380993\|emb\|CAR63124.1\| | Hypothetical protein [Homo sapiens] | 204 | 1,44E^-18^ | 36/40 (90%) |
|  |  | 2)MHLTRTLWFWEGRSSLGRKIHASPPYIMVLGGAVTL | gi\|242380992\|emb\|CAR63123.1\| | Hypothetical protein [Homo sapiens] | 139 | 1,35E^-10^ | 30/34 (88.24%) |
| **24** | HG531482 | 1)MHLTRTLWFWEGRSSLGRKIHASPPYIMVLGGAFKFGPENSCISPVHYGFGKGGSENNVS | gi\|242380993\|emb\|CAR63124.1\| | Hypothetical protein [Homo sapiens] | 189 | 8,09E^-17^ | 34/40 (85%) |
|  |  | 2)MVLGGAFKFGPENSCISPVHYGFGRGVQVWAGKFMHLTRTLWFWEGRK | gi\|242380993\|emb\|CAR63124.1\| | Hypothetical protein [Homo sapiens] | 142 | 3,02E^-10^ | 26/26 (100%) |
|  |  | 3)MHLPRTLWFWEGRSSLGRKIHASHPYIMVLGREEVRIMLANHINASKAYCE | gi\|242380992\|emb\|CAR63123.1\| | Hypothetical protein [Homo sapiens] | 135 | 1,06E^-09^ | 28/31 (90.32%) |
| **24** | HG531483 | MHLTRTLWFWEGRSNLGRKIHASPPYIMVLGGAFKFGPGNSCVSPVHYGFGRGVQVWAGKFMHLPRTLWFWEGASWIRAFHFPLIFVS | gi\|242380947\|emb\|CAR63104.2\| | Hypothetical protein [Homo sapiens] | 258 | 3,16E^-27^ | 48/54 (88.89%) |
| **25** | HG531485 | 1)MCIRACYWSIQRLNFFLVWFWEGPSNLWPKNHASPPYIIIQDLWDTYKV | gi\|242380950\|emb\|CAR63106.1\| | Hypothetical protein [Homo sapiens] | 94 | 0,00089636 | 18/18 (100%) |
|  |  | 2)MAEKSCISPVHYNTRSMGYLQGLNIKEKEILLYTEYC | gi\|328876072\|gb\|EGG24436.1\| | RhoGAP domain-containing protein [Dictyostelium fasciculatum] | 70 | 9,70799 | 11/27 (40.74%) |
| **25** | HG531486 | MCIRACYWSIQRLNFFLVWFGFGRGVQIWAGKFMHLTRTLSQFRGAVLVYQTYHYAHTSSFYLWLVLRL | gi\|242380874\|emb\|CAR63079.1\| | Hypothetical protein [Homo sapiens] | 97 | 0,0007909 | 17/18 (94.44%) |
| **25** | HG531487 | 1)MHLTCTLWFWEGGSYHHTPSMQPFIQLVLLLPTSQSSTGKTEEQLPVLPIPSALPKRYKAFLNSLQFCSCGLL | gi\|388546541\|ref\|ZP_10149815.1\| | Patatin [Pseudomonas sp. M47T1] | 77 | 2,11059 | 15/37 (40.54%) |
|  |  | 2)MVLGGRFIPSHTIYAAIHSIGPASAHLSVFNWKD | -- | No significant similarity | -- | -- | -- |
| **25** | HG531488 | 1)MLHMRANGTRSYSDINNVHFSISGCVERPSQTIFHEFPYLILRDHNTNQSYTPLTKYINCI | gi\|255568418\|ref\|XP_002525183.1\| | DNA replication licensing factor MCM8, putative [Ricinus communis] | 74 | 4,31055 | 15/30 (50%) |
|  |  | 2)MVTEYEVWKFVKYGLGGAFDTSRNAEMNVVDVTVAPCSVCSHVQHTSVLQKKVGIKEKTVVIS | gi\|242380904\|emb\|CAR63089.1\| | Hypothetical protein [Homo sapiens] | 69 | 3,36168 | 13/13 (100%) |
| **25** | HG531489 | 1)MEIREILILGGAFKFMAEKSCISPVHYGFGRGVQIWAGKFMHLTRTLY | gi\|242380993\|emb\|CAR63124.1\| | Hypothetical protein [Homo sapiens] | 124 | 1,33E^-07^ | 22/27 (81.48%) |
|  |  | 2)MHLPRTLWFWEGRSNLGRKIHASHPYIILKDLWKWHHIKGKGKCRV | gi\|242380877\|emb\|CAR63080.1\| | Hypothetical protein [Homo sapiens] | 117 | 5,43E^-07^ | 21/27 (77.78%) |
| **26** | HG531490 | MSLFTEFPKDVLNYSQFSDEAPSQNQHFLKFPNPFYYPTYTQTTNKTLL | -- | No significant similarity | -- | -- | -- |
| **26** | HG531491 | 1)MVGLDDPGGLFQSQPSDDSVASPPYIICPKMGCVYGRWGSIGVGVI | gi\|242381091\|emb\|CAR63172.1\| | Hypothetical protein [Homo sapiens] | 69 | 2,16899 | 14/18 (77.78%) |
|  |  | 2)MILEVFSNHNHLMILWHLPRTLYARKWGVFTGGGVRLGLV | gi\|242380968\|emb\|CAR63114.1\| | Hypothetical protein [Homo sapiens] | 109 | 7,06E^-06^ | 21/24 (87.5%) |
| **26** | HG531492 | MGLHLTRTLYAENGVCLRGVGFDWGWCNIRGWELKTGECLT | gi\|242380982\|emb\|CAR63119.1\| | Hypothetical protein [Homo sapiens] | 86 | 0,0139569 | 17/21 (80.95%) |
| **26** | HG531493 | MHKFPPQKLTSTPTLSGLQLPSPYITPTPIEPLFFQVEGT | gi\|242381090\|emb\|CAR63171.1\| | Hypothetical protein [Homo sapiens] | 149 | 5,84E^-12^ | 31/32 (96.88%) |
| **26** | HG531494 | MVVRLGLVYSEIQLLPGLVLGGAFKFGPEKSCISPVHYGFGRGGRPLLLHN | gi\|242380988\|emb\|CAR63121.1\| | Hypothetical protein [Homo sapiens] | 138 | 3,81E^-10^ | 28/31 (90.32%) |
| **26** | HG531496 | 1)MAVRLGLVYSEIQLLPGLVLGGAFKFGPENSCISPVHYGFGRGGRPLFLCNKQ | gi\|242380993\|emb\|CAR63124.1\| | Hypothetical protein [Homo sapiens] | 144 | 1,92E^-10^ | 26/27 (96.3%) |
|  |  | 2)MVLGGGVDLFSYATSNRVRGNGLKLHQGMFRILGKNSYHK | gi\|363729350\|ref\|XP_003640634.1\| | PREDICTED: uncharacterized protein LOC100857505 [Gallus gallus] | 97 | 0,00190102 | 20/34 (58.82%) |
| **27** | HG531497 | MCITVICWSLFVGRWYVWFDWGWCNIACVVGVILCSIIVCV | -- | No significant similarity | -- | -- | -- |
| **27** | HG531499 | MCITVICWSLFVGRWYVWFDWGWCNIGLVCWRFKNLLSNVMYVIGFCDI | -- | No significant similarity | -- | -- | -- |
| **27** | HG531505 | MGSPSLKVLQKCGDVTLREWSVGMVWHGGFDWGWCNIGCVDLGYI | gi\|242380614\|emb\|CAR63070.1\| | Hypothetical protein [Homo sapiens] | 92 | 0,00126041 | 17/17 (100%) |
| **28** | HG531509 | MLLYCVILCLCKLYYKMWLEIVETLVLGGAFNILEVSSDKC | -- | No significant similarity | -- | -- | -- |
| **36** | HG531510 | MGSPSLK?LQKCGDVTLREWSVGMVWHGGFDWGWCNIGLGC | -- | No significant similarity | -- | -- | -- |
| **36** | HG531511 | MQEFSAKKLNAPPKTTNSQNFIPLILATTPTTTHNTPIPIPYHYTFYNILIINIHTLLTTFTIQTFHLYQCTHNTFIYYHTIT | gi\|40716547\|gb\|AAR88804.1\| | Putative chimeric protein 173-56 [Trypanosoma cruzi] | 70 | 3,41751 | 15/19 (78.95%) |
| **45** | HG531518 | 1)MVRHRNRLPIEVVEPPSQN??FTNFHTSYSVTITQTQSYNPINQIYKL | -- | No significant similarity | -- | -- | -- |
|  |  | 2)MGLYDCVCVMVTEYEVWKFVK?WFWEGGSTTSMGNL?RCLTILTLKNFFLELDLV | -- | No significant similarity | -- | -- | -- |
| **54** | HG531521 | MHKQLLTNPQSNSQLTPQAAEERDERPSQNRTFGKFSTLLTP | -- | No significant similarity | -- | -- | -- |
| **55** | HG531523 | 1)MANLLNTKIEQERTRRPLPKPIFHESPYLILRNHNTNTIIQPH | -- | No significant similarity | -- | -- | -- |
|  |  | 2)MHDFSGPNLNAPPKTRKPNQNKVHVYTSKNKQTKAIFFSHNNFTVLTKHLVTAFLTK | gi\|242380935\|emb\|CAR63099.1\| | Hypothetical protein [Homo sapiens] | 86 | 0,016547 | 15/16 (93.75%) |
|  |  | 3)MGLYDCVCVMVTEYEVWRFVKYWFWEGPSSPLLFNFSIKQVCHCDQYYFVRNAVTKCFVSTVKLL | gi\|242380904\|emb\|CAR63089.1\| | Hypothetical protein [Homo sapiens] | 108 | 1,42E^-05^ | 19/20 (95%) |
| **55** | HG531524 | MHEFPPQKLTPTPTLSGLRLPSPYMTPTPIEPRPKPENQTKTKFTSILQKTNKQKLFFSLTITSQCLQNI | gi\|242381021\|emb\|CAR63138.1\| | Hypothetical protein [Homo sapiens] | 151 | 1,30E^-11^ | 30/32 (93.75%) |
| **57** | HG531525 | 1)MKEFLYSPTGKFYGLNNAWPFQVLKNFEFSFNEVNAVIMYG | -- | No significant similarity | -- | -- | -- |
|  |  | 2)MCIRARYWSIQRFNFFLVWFWEGRSNLGPKNHASHPYIITALTSLKENSKFLST | gi\|242380949\|emb\|CAR63105.1\| | Hypothetical protein [Homo sapiens] | 91 | 0,00339309 | 17/18 (94.44%) |
| **57** | HG531526 | MHLPRTLYARKWGVFTGGGGSIGVGVCVSEPVIGLFRDSTSSWFGFGRGVHTRWK | gi\|7959748\|gb\|AAF71046.1\|AF116721_23 | PRO1094 [Homo sapiens] | 84 | 0,0334751 | 18/25 (72%) |
| **57** | HG531527 | 1)MCI??CYWSIQRFN?FLVWFWEGRSNL?PKNHASHPYIICPKMGCVYGRRWFDWGWCMCIRACYWSIQRFNFFLVWFWEGGSRSFPNVFLGKHKRSI | -- | No significant similarity | -- | -- | -- |
|  |  | 2)M?EKSCISPVHYMPENGVCLREAVVRLGLVYVYQSLLLVYSEIQLLPGLVLGGGFKVLS | -- | No significant similarity | -- | -- | -- |
|  |  | 3)MHLTRTLYARKWGVFTGGGGSIGVGVCVSEPVIGLFRDSTSSWFGFGRGVQGPFLMCF | gi\|7959748\|gb\|AAF71046.1\|AF116721_23 | PRO1094 [Homo sapiens] | 83 | 0,0487778 | 17/20 (85%) |
| **57** | HG531528 | 1)MKTTTMHSTFENYILTIFSKELVESKALIHIHQPQSNHHLP | -- | No significant similarity | -- | -- | -- |
|  |  | 2)MCIRACYWSIQRFNFFLVWFWEGPSNLWPKNHASPPYVICPKMGCVYGRWWFDWGWCMCIRALLSTSSLEKIVRM | gi\|242380949\|emb\|CAR63105.1\| | Hypothetical protein [Homo sapiens] | 98 | 0,00060227 | 18/18 (100%) |
|  |  | 3)MAEKSCISPVRYMPENGVCLREVVVRLGLVYVYQSLALYQLLGKDSQNVIFKSTVHCSCFHSSVYLYFCFLLVIKPFSLLFWYGLCEHLMPAV | gi\|242380630\|emb\|CAR63076.1\| | Hypothetical protein [Homo sapiens] | 98 | 0,00091204 | 19/26 (73.08%) |
|  |  | 4)MHLPRTLYARKWGVFTGGGGSIGVGVCVSEPCSLPAPWKR | -- | No significant similarity | -- | -- | -- |
| **57** | HG531529 | 1)MYIRACYWSIQRFNFFLVWFWEGGSNLWPKNHASPPYVICPKMGCVYGRWWFDWGWCMYIRACYWSIQRFNFFLVWFWEGGSGWILGKISSPKEWSGTGTGCPRSW | gi\|242380969\|emb\|CAR63115.1\| | Hypothetical protein [Homo sapiens] | 117 | 1,80E^-06^ | 22/24 (91.67%) |
|  |  | 2)MHLPRTLYARKWGVFTGGGGSIGVGVCISEPVIGLFRDSTSSWFGFGRGVQVGY | gi\|7959748\|gb\|AAF71046.1\|AF116721_23 | PRO1094 [Homo sapiens] | 87 | 0,0120707 | 18/21 (85.71%) |
| **57** | HG531530 | MCIRACYWS?QRFNFFLVWFWRGVQIYGRKIMHLTRTLLHVVLWEKG | -- | No significant similarity | -- | -- | -- |
| **57** | HG531531 | 1)MCIRACYWSIQRFNFFLVWFWEGRSNLWPKNHASHPYIICPKMGCVYGRRWFDWGWCMYIRACYWSIQRFNFFLVWFWEGGGFKFM?EKSCISPVHYMPENGVCLREVVVRLGLVYVYQ?LLLVYSEIQLLPGLVLGGGFK?LS | -- | No significant similarity | -- | -- | -- |
|  |  | 2)MAEKSCISPVHYMPENGVCLREAVVRLGLVYVYQSLLLVYSEIQLLPGLVLGGRGVQIY?RKIMHLTRTLYARKWGVFTGGGGSIGVGVCVS?PVIGLFRDSTSSWFGFGRGLQ?PFLMCF | -- | No significant similarity | -- | -- | -- |
|  |  | 3)MHLTRTLYARKWGVFTGGGGSIGVGVCISEPVIGLFRDSTSSWFGFGREGGSNL?PKNHASHPYIICPKMGCVYGRWWFDWGWCMCIR?CYWSIQRFNFFLVWFWEGAS?SFPNVFLGKHKRSI | -- | No significant similarity | -- | -- | -- |
| **58** | HG531533 | 1)MKEFLYSPTGKFYGLNNAWPFQVLKNFEFSFNEVNAVIMYG | -- | No significant similarity | -- | -- | -- |
|  |  | 2)MAEKSCISPVRYMPENGVCLREVVVRLGLVYVYQNLLLVYSEIQLLPGLVLGGAFKFMAEKSCISPVHYHSINLIERKFKVFEHLKWPSIIQTIEFTRRRI | gi\|242380982\|emb\|CAR63119.1\| | Hypothetical protein [Homo sapiens] | 94 | 0,00497523 | 18/21 (85.71%) |
|  |  | 3)MHLPRTLYARKWGVFTGGGGSIGVGVCVSEPVIGLFRDSTSSWFGFGRGLQIYGRKIMHLTRTLSQH | gi\|7959748\|gb\|AAF71046.1\|AF116721_23 | PRO1094 [Homo sapiens] | 84 | 0,050035 | 17/20 (85%) |
|  |  | 4)MGCVYGRWWFDWGWCMCIRTCYWSIQRFNFFLVWFWEGPSNLWPKNHASHPYIITALTSLKENSKFLST | gi\|242380969\|emb\|CAR63115.1\| | Hypothetical protein [Homo sapiens] | 124 | 7,83E^-08^ | 22/24 (91.67%) |
| **58** | HG531534 | 1)MKEFLYSPTGKFYGLNNAWPFQVLKNFEFSFNEVNAVIMYG | -- | No significant similarity | -- | -- | -- |
|  |  | 2)MAEKSCISPVRYMPENGVCLREVVVRLGLVYVYQSLLLVYSEIQLLPGLVLGGAFKFMAEKSCISPVHYTRASHPYIIVLGGGAKFGPENSCISPVHYHSINLIERKFKVFEHLKWPSIIQTIEFTRRRI | gi\|242380982\|emb\|CAR63119.1\| | Hypothetical protein [Homo sapiens] | 126 | 3,46E^-07^ | 26/29 (89.66%) |
|  |  | 3)MHLPRTLYARKWGVFTGGGGSIGVGVCVSEPVIGLFRDSTSSWFGFGRGVQIYGRKIMHLTRTLYKGISPVHYSFGRGGQIWAGKFMHLTRTLSQH | gi\|242380947\|emb\|CAR63104.2\| | Hypothetical protein [Homo sapiens] | 127 | 9,12E^-08^ | 24/26 (92.31%) |
| **58** | HG531535 | 1)MLSNDLKKKKKDKTQQNG?LYKSRG?HRPFP?AERHRPPLPKPQILKIL?? | -- | No significant similarity | -- | -- | -- |
|  |  | 2)MV?CTNPEE?TDPSLPPSGTDPPSQNHKFSKFY??NFSNNTYYHTQ??NSD?TPLYIL | -- | No significant similarity | -- | -- | -- |
| **58** | HG531537 | MSAKHLRGSTRSESYKGHILCMFLVSVILAQSDPPSQNHKFSKFYTTNFSNNTYYHTQHTNSDTTPLYIL | gi\|260948314\|ref\|XP_002618454.1\| | Hypothetical protein CLUG_01913 [Clavispora lusitaniae ATCC 42720] | 75 | 3,68887 | 14/32 (43.75%) |
| **58** | HG531539 | 1)MNAKGAYGKSVVQFQPPTEGKHFENIKTPPVLPAEKCPLQGKKMRNVSGHPHLLRCPRLISWERPSQNQYFTNFHTSYSVTITQTQSYNPIDQIYKL | -- | No significant similarity | -- | -- | -- |
|  |  | 2)MGLYDCVCVMVTEYEVWKFVKYWFWEGRSHEMSLGQRRR | gi\|242380904\|emb\|CAR63089.1\| | Hypothetical protein [Homo sapiens] | 103 | 3,17E^-05^ | 22/28 (78.57%) |
| **58** | HG531540 | MKEFLYSPTGKFYGLNNAWPFQVLKNFEFSFNEV?AVIMYGGDA | -- | No significant similarity | -- | -- | -- |
| **58** | HG531541 | MHLTRTLYKGISPVHYSFGRGGQIWAGKFMHLPRTL | gi\|242380947\|emb\|CAR63104.2\| | Hypothetical protein [Homo sapiens] | 133 | 1,55E^-09^ | 25/26 (96.15%) |
| **59** | HG531544 | 1)MKEFLYSPTGKFYGLNNAWPFQVLKNFEFSFNEVNAVIMYG | -- | No significant similarity | -- | -- | -- |
|  |  | 2)MEIREILILGGAFKFMAEKSCISPVHYHSINLIERKFKVFEHLKWPSIIQTIEFTRRRI | gi\|242380993\|emb\|CAR63124.1\| | Hypothetical protein [Homo sapiens] | 99 | 0,0004731 | 17/22 (77.27%) |
| **59** | HG531545 | 1)MKEFLYSPTGKFYGLNNAWPFQVLKNFEFSFNEVNAVIMYG | -- | No significant similarity | -- | -- | -- |
|  |  | 2)MPENGSCRPENVVLGGAFKFGPENSCISPVHYGFGRGVQIWAGKFMHLTRTLWFWEGRSNLGRKIHASHPYIITALTSLKENSKFLST | gi\|242380993\|emb\|CAR63124.1\| | Hypothetical protein [Homo sapiens] | 142 | 1,37E^-09^ | 26/29 (89.66%) |
|  |  | 3)MGAVDRRTWFWEGRSSLGRKIHASPPYIMVLGGAFKFGPENSCISPVHYGFGRGVQIWAGKFMHLTRTLSQH | gi\|242380947\|emb\|CAR63104.2\| | Hypothetical protein [Homo sapiens] | 229 | 3,26E^-23^ | 44/47 (93.62%) |
|  |  | 4)MHLPRTLWFWEGRSNLGRKIHASHPYIMVLGGAFKFGPENSCISPVHYHSINLIERKFKVFEHLKWPSIIQTIEFTRRRI | gi\|242380993\|emb\|CAR63124.1\| | Hypothetical protein [Homo sapiens] | 170 | 1,13E^-13^ | 30/34 (88.24%) |
| **59** | HG531546 | 1)MHFGKGECVLLNCLKRRTYMKEFLYSPTGKFYGLNNAWPFQVLKNFEFSFNEVNAVIMYG | gi\|347524893\|ref\|YP_004831641.1\| | FIST domain containing protein [Lactobacillus ruminis ATCC 27782] | 79 | 0,977875 | 19/50 (38%) |
|  |  | 2)MPGNGSCRPENVVLGGAVKFGPENSCISPVHYHSINLIERKFKVFEHLKWPSIIQTIEFTRRRI | gi\|242380993\|emb\|CAR63124.1\| | Hypothetical protein [Homo sapiens] | 107 | 4,50E^-05^ | 20/24 (83.33%) |
|  |  | 3)MGAVDRRTWFWEGQSNLGRKIHASHPYIITALTSLKENSKFLST | gi\|242380877\|emb\|CAR63080.1\| | Hypothetical protein [Homo sapiens] | 157 | 6,52E^-13^ | 27/30 (90%) |
| **60** | HG531550 | MLGQQDMQSAERPSQNQLFGIFNLLFIGITPQQLSPIP | -- | No significant similarity | -- | -- | -- |
| **60** | HG531551 | MDEPPSQNQYFTNFHTSYSVTITQTQSYNPINQIYKL | gi\|137277\|sp\|P06724\|V30K_HCMVE | 30 kDa major early protein | 73 | 2,31834 | 16/34 (47.06%) |
| **60** | HG531552 | MLLILLVAHEPPSQNQLFGIFNLLFIGITPQQLSPIP | -- | No significant similarity | -- | -- | -- |
| **60** | HG531553 | 1)MVLPIKCMLREFISLLSLTPPPKTNISRISIPHTP | -- | No significant similarity | -- | -- | -- |
|  |  | 2)MGLYDCVCVMATEYEVWKFVKYWFWEGGSKKAN | gi\|242380904\|emb\|CAR63089.1\| | Hypothetical protein [Homo sapiens] | 97 | 0,00025061 | 18/20 (90%) |
|  |  | 3)MEIREILVLGGGVKESKLMNSLSMHLIGNTILLTVSSLKCARLWWTQRETSINQEGQYYCSGTELCLQEIAALGGKFHCNMQLAIETAPAFQVGQLHWTASNQKTIFPRS | gi\|33862812\|ref\|NP_894372.1\| | Phosphoribosylformylglycinamidine synthase I [Prochlorococcus marinus str. MIT 9313] | 75 | 7,5392 | 24/72 (33.33%) |
| **60** | HG531556 | 1)MRHTRTLWFWEGRSNLGRKIHASHPYIMNRGLQICTHILTIPLSASR | gi\|242380892\|emb\|CAR63085.1\| | Hypothetical protein [Homo sapiens] | 119 | 3,89E^-07^ | 22/28 (78.57%) |
|  |  | 2)MVLGGAFKFGPENSCISPVHYESRTSDLYPYFNYSIVCLSLRI | gi\|242380993\|emb\|CAR63124.1\| | Hypothetical protein [Homo sapiens] | 118 | 7,30E^-07^ | 21/21 (100%) |
| **62** | HG531559 | MGSPSLKVLQKCGDVTLREWSVG??WHGGF?WGWCNIG?VD?GYI | -- | No significant similarity | -- | -- | -- |
| **62** | HG531560 | MGSPSLKVLQKCGDVTLREWSVGMVWHGGFDWGWCNIALVIG | -- | No significant similarity | -- | -- | -- |
| **62** | HG531563 | 1)MQEKTPPPKTIISEISYPRLCSNISTKRYHYTPPTTKLTMQTYKIYNIY | -- | No significant similarity | -- | -- | -- |
|  |  | 2)MVLGGGVFSCINSKRTRGNGLKLCQGRFRLDI?NNFFSKIVVRH | -- | No significant similarity | -- | -- | -- |
| **66** | HG531565 | MGSPSLKVLQKCGDVTLREWSVGMVWHGGFDWGWCNIDRGGFMDLIL | gi\|358253606\|dbj\|GAA53490.1\| | Dipeptidyl peptidase 4 [Clonorchis sinensis] | 78 | 1,40468 | 15/37 (40.54%) |
| **66** | HG531566 | MGSPSLKVLQKCGDVTLREWSVGMVWHGGFDWGWCNIGLVCWRFKNLLSNVMYVIGFCDI | gi\|358253606\|dbj\|GAA53490.1\| | Dipeptidyl peptidase 4 [Clonorchis sinensis] | 73 | 6,10756 | 14/35 (40%) |
| **66** | HG531567 | MGSPSLKVLQKCGDVTLREWSVGMVWHGGFDWGWCNIRRLVD | gi\|242380955\|emb\|CAR63108.1\| | Hypothetical protein [Homo sapiens] | 80 | 0,0698678 | 14/14 (100%) |
| **67** | HG531573 | 1)MLLFGSSEGQGCWDPFLFKCCLGSIGVGVCVSELVTGLFRDSNFFLV | gi\|168701252\|ref\|ZP_02733529.1\| | Hypothetical protein GobsU_17136 [Gemmata obscuriglobus UQM 2246] | 73 | 3,37759 | 11/23 (47.83%) |
|  |  | 2)MLSGFDWGWCMCIRACYWSIQRFKLLPGLV?GGCMCIRACYWSIQRLNFLLV | -- | No significant similarity | -- | -- | -- |
| **67** | HG531576 | MRRGEKWGAWRTSVIPFSSPQQISCVTVDESFSISPVHYMPENGVCLREVGFDWGWCNIWRLVD | gi\|242380982\|emb\|CAR63119.1\| | Hypothetical protein [Homo sapiens] | 128 | 4,95E^-08^ | 24/30 (80%) |
| **68** | HG531580 | 1)MYISHTSHIQIHTIHTNQTINTHSIFHLISIHSNKPYITPTPIEPTNQPKKPNKNQTKPNHNNKKNPSKV | -- | No significant similarity | -- | -- | -- |
|  |  | 2)MVSGLSFHTFDGFFLLLWFGLVWFLFGFFGWLVGSIGVGVI | -- | No significant similarity | -- | -- | -- |
| **68** | HG531581 | MGSPSLKVLQKCGDVTLREWSVGMVWHGGFDWGWCNIGFWLVVWLVYIMGLYIF | gi\|297616374\|ref\|YP_003701533.1\| | Hypothetical protein Slip_0168 [Syntrophothermus lipocalidus DSM 12680] | 75 | 3,28645 | 13/35 (37.14%) |
| **71** | HG531582 | MGSPSLKVLQKCGDVTLREWSVGMV??GGFDWGWCKV | -- | No significant similarity | -- | -- | -- |
| **73** | HG531584 | MHLTRTLWFWEGRSNLGRKIHASHLYIMVLGGAFKFGPENSCISPVHYGFGRGVQVGY | gi\|242380993\|emb\|CAR63124.1\| | Hypothetical protein [Homo sapiens] | 188 | 1,21E^-16^ | 34/39 (87.18%) |
| **74** | HG531586 | MKAVLYGRAGLAGSKWHQEAKRLNPPPKTTLSGNSQPYSHPKYTK | -- | No significant similarity | -- | -- | -- |
| **74** | HG531587 | MSLLKSFFCNFTFLLNPPPKTTLSGNSQPYSHPKYTK | -- | No significant similarity | -- | -- | -- |
